# Assessing dengue knowledge, attitudes and preventive practices in France and Italy: A cross-sectional study using participatory surveillance cohorts

**DOI:** 10.1371/journal.pgph.0007162

**Published:** 2026-09-23

**Authors:** Lucille Calmon, Alessandro De Gaetano, Mattia Mazzoli, Nicolò Gozzi, Marion Debin, Clément Turbelin, Romain Marmorat, Nicola Perra, Alain Barrat, Vittoria Colizza, Daniela Paolotti

**Affiliations:** 1 Sorbonne Université, INSERM, Pierre Louis Institute of Epidemiology and Public Health, Paris, France; 2 ISI Foundation, Turin, Italy; 3 Aix Marseille Univ, Université de Toulon, CNRS, CPT, Marseille, France; 4 School of Mathematical Sciences, Queen Mary University of London, London, United Kingdom; 5 The Alan Turing Institute, London, United Kingdom; University of Oslo Faculty of Medicine: Universitetet i Oslo Det medisinske fakultet, NORWAY

## Abstract

The dengue virus, transmitted by *Aedes* mosquitoes, causes an estimated 100–390 million infections annually worldwide. With no treatment available, mitigation and prevention involve reducing mosquito breeding and limiting feeding opportunities. Due to changing climate conditions, *Aedes* mosquitoes are increasingly established in Europe causing more local cases. Thus, understanding population knowledge, attitudes, and practices in areas where dengue is an emerging threat is necessary for effective prevention. We designed a cross-sectional survey collecting knowledge, attitudes, and beliefs about dengue in the general population, leveraging the French and Italian 2024 cohorts of the participatory surveillance network InfluenzaNet. We analyzed pooled responses from 2,500 participants in mainland France and 404 in Italy. Via bias-adjusted (age, sex and education) logistic and ordinal regression, we examined determinants of awareness of dengue, knowledge of its characteristics, adoption of preventive measures, and vaccination willingness. Over 91% of included participants were aware of dengue as a disease, and 14.6% had sought information. Seeking information and concern levels were significant determinants of dengue knowledge, preventive measures adoption, and vaccination willingness. Better knowledge of dengue characteristics, vaccination status, residence in areas with detected autochthonous cases, and presence of comorbidities were associated with greater adoption of preventive measures. Living in rural areas was associated with lower knowledge and vaccination willingness but higher adoption of preventive measures. Finally, 0.4% of participants had received a dengue vaccine (not recommended to the general population), and 54.6% would accept a vaccine if offered. Low-risk perception was the most common reason for refusal. Our study highlights the interplay between knowledge, attitudes, behaviours, socio-demographic, individual and contextual characteristics of the participants related to dengue in a non-endemic context. Targeted communication strategies accounting for these factors are essential to enhance preparedness and outbreak prevention in regions where dengue represents an emerging threat.

## 1. Introduction

The virus dengue [1], transmitted to humans via the *Aedes aegypti* and *Aedes albopictus* mosquitoes, causes an estimated 100–390 million infections, and more than 40 thousand deaths worldwide each year [2–4]. While a vaccine granting moderate protection is recommended in regions with high dengue transmission [5,6], there is currently no medical cure for dengue. Consequently, strategies to prevent and manage outbreaks heavily rely on vector control measures limiting mosquito proliferation and breeding, as well as feeding opportunities [7–10]. Community involvement in these strategies is paramount to maintain engagement with prevention programs [11,12], which can present a challenge in newly affected regions where surveillance programs were only recently established [13].

Dengue transmission has been historically concentrated in the tropics [14], but regions previously spared are increasingly at risk due to changing climatic conditions [15–17]. Models accounting for different climate scenarios predict the future expansion of *Aedes* mosquitoes on the European continent [18,19], possibly leading to sustained dengue transmissions as climatic conditions become better suited to the spread of the disease [20]. In Europe, autochthonous cases of dengue have been increasing since 2010 [21,22], especially in Italy, France, Spain and Greece. In 2024, an outbreak of at least 199 autochthonous cases was observed in Italy [23], while 11 outbreaks totalling 83 autochthonous cases were detected in France [24]. In 2025, guidance from the ECDC on the assessment and mitigation of *Aedes*-borne diseases anticipated an increase in the risk of local outbreaks in Europe [25]. This risk is particularly high for France, which reports large numbers of imported dengue cases into mainland France from its overseas departments where dengue is endemic, including La Réunion and Les Antilles (Martinique and Guadeloupe). In the first quarter of 2024 for example, 82% of the 1679 imported dengue cases in mainland France originated from Les Antilles [26]. In this context, the dengue vaccine Qdenga was approved for use in Europe in 2022, but vaccinated individuals may be at a higher risk of developing a severe form of dengue if vaccination occurred in immune naive individuals. As a result, Qdenga is only recommended in specific cases: in the French overseas departments where dengue is circulating, it is recommended to children who can demonstrate a previous infection, and vulnerable adults. In mainland France, the vaccine was not recommended at the time of the study given the low levels of circulation of dengue and the possible risk of severe forms of dengue associated with infection following vaccination of immune-naive individuals. Recommendations were updated in 2025 to allow vaccination of travellers on a case by case basis. In Italy, it is recommended to travellers to endemic areas, for long (at least three weeks) or repeated trips, especially if the traveller has been previously infected with dengue.

Promoting awareness and behavioural change to reduce the proliferation of mosquitoes and the transmission of dengue are pillars of public health strategies [27,28]. According to the health belief model, behavioural adaptations are generally shaped by the interplay of risk perception, perceived benefits of behavioural change, cues to actions and individual socio-demographic and psychological characteristics [29]. To effectively inform the population and encourage the adoption of preventive measures through tailored prevention programs, it is important to understand factors shaping knowledge, attitudes, prevention practices, and their interplay specific to mosquito-borne viruses and in particular dengue [30]. In endemic areas where the population is knowledgeable on mosquito-borne viruses characteristics and commonly adopt preventive measures [31–34], these factors have been extensively investigated. Professional inactivity, lower education level, and older age were shown to be associated with worse knowledge of dengue characteristics in La Réunion [31], while exposure to information on dengue, previous dengue infection and higher concern levels instead were associated with knowledge of dengue symptoms and transmission in Venezuela [32]. Knowledge about dengue itself was shown to drive the adoption of preventive practices in Venezuela [32] and Malaysia [35], but not in La Réunion [31]. The adoption of preventive practices was also more likely in women, individuals with higher education level, and those feeling to be frequently bitten by mosquitoes in La Réunion [31], as well as for individuals reporting higher perceived susceptibility in Venezuela [32] and perceived threat to society in Hong Kong [33].

In non-endemic regions, dengue specific knowledge, attitudes, preventive practices adoption and their drivers have been investigated in a few instances [36]. In Italy, Italians in the Lazio region were less knowledgeable on mosquito-borne viruses, and mosquito life cycles, compared to resident communities originally from the Indian subcontinent in 2016 [37]. Italians also reported fewer concerns for mosquito-borne viruses but higher annoyance with mosquitoes [37]. In French metropolitan departments colonised by *Aedes* mosquitoes, concern levels, annoyance with mosquitoes, perceived exposure to mosquito-borne diseases and perceived efficacy of preventive practices were associated with the adoption of preventive practices in 2016 [38], together with sex, education status, and previous identification of *Aedes albopictus* mosquitoes in 2012 [39,40]. Longitudinal observations between 2012 and 2014 in a randomly selected sample from colonized departments revealed increasing awareness of dengue transmission [39]. However, knowledge of the characteristics of other mosquito-borne viruses remained heterogeneous in both France [40] and Italy [37], while the adoption of preventive measures stayed moderate between 2012 and 2016 [37,39,40]. The majority of studies in non-endemic regions however relied on small samples [36,41], or focused on specific subpopulations, such as amateur gardeners [42], medical professionals [43] or residents in departments colonised by *Aedes* mosquitoes [38–40]. Previously evaluated levels of knowledge, attitudes, and preventive practices in European populations, and their determinants are likely to have significantly evolved with the rapidly evolving establishment of *Aedes* vectors in Europe [44], the increasing detection of locally transmitted dengue cases [21], and following the COVID-19 pandemic which affected population awareness of health threats. It is therefore of high importance to assess current levels of dengue knowledge, attitudes and practices, and identify their drivers in order to inform the design of future prevention campaigns.

In this context, the aims of our study are two-fold: (i) establish an updated comprehensive assessment of levels of knowledge, attitudes and preventive practices in two countries where dengue has recently spread locally; and (ii) identify determinants of knowledge, attitudes and practices in order to inform future policy campaigns aimed at raising awareness and encouraging preventive measure adoption, in regions where the threat posed by dengue is projected to increase.

Participatory surveillance platforms [45] like the European InfluenzaNet cohort network [46] provide an efficient and low cost tool for these purposes, by leveraging volunteer participants continuously engaged with the platform. In parallel to the longitudinal respiratory syndromic surveillance conducted weekly, additional cross-sectional surveys can be rapidly deployed, as needed, e.g., to understand vaccination attitudes [47,48] and behavioural changes [49]. This is of particular relevance when facing emerging threats where rapid insight is necessary, for example to capture risk perception at the start of the COVID-19 pandemic [50].

In this study, we conducted a cross-sectional survey of the participatory cohorts of InfluenzaNet [46] to evaluate knowledge, attitudes and practices in mainland France (including Corsica) and Italy, two territories increasingly exposed to dengue. The survey included questions on knowledge of dengue (i.e., its existence and main characteristics), information sources, perceived level of information, concern levels related to a set of consequences of a dengue outbreak, perceived efficacy of a set of preventive measures, adoption of these measures, attitudes towards vaccination, including reasons for refusal. We also enquired about possible recent dengue symptoms, and knowledge of a dengue case among relatives and acquaintances. A detailed profile for each participant including socio-demographic and health characteristics was also obtained from the InfluenzaNet platform. Using the gathered data, we first described the levels of knowledge of dengue characteristics, information seeking behaviours, measures adoption and perception of efficacy, as well as fears related to the disease, and attitudes towards vaccination. Second, we conducted a statistical analysis to characterise factors associated with awareness of the existence of dengue, knowledge of its main characteristics, adoption of preventive measures and attitudes towards vaccination, in the scenario of a vaccine being offered. The findings of this study provide essential insights on knowledge, attitudes and practices in two European contexts, paving the way to the targeted design of interventions to raise awareness and encourage the adoption of preventive practices.

## 2. Methods

### 2.1. Ethics statement

This study was conducted in agreement with country-specific regulations on privacy and data collection and treatment. It involved exclusively adult participants. Informed written consent was obtained from all participants enabling the collection, storage, and treatment of data, and their publication in anonymized, processed, and aggregated forms for scientific purposes. In addition, approvals by Ethical Review Boards or Committees were obtained, where needed according to country-specific regulations. The authors coordinated the data collection via the Grippenet/Covidnet and Influweb platforms. Data collection occurred between May 13, 2024, and October 27, 2024, in Italy and between July 29, 2024, and August 26, 2024, in France. The authors accessed the pseudonymized dataset for analysis during this same period.

In France, the Grippenet/Covidnet study was approved by the Comité consultatif sur le traitement de l’information en matière de recherche (CCTIRS, Advisory committee on information processing for research, authorization 11.565) and by the Commission Nationale de l’Informatique et des Libertés (CNIL, French Data Protection Authority, authorization DR-2012–024). This authorization included the possibility to add non-identifying questions or questionnaires, in line with the research objective (surveillance of diseases in the general population). The present study falls under the scope of this authorisation.

For influweb.it the research was conducted in accordance with Italian Data Protection Authority (Garante per la protezione dei dati personali) regulations on privacy, data collection and treatment, reported here https://www.garanteprivacy.it/web/garante-privacy-en. The Italian Data Protection Authority adheres to the norms set by the European Union’s General Data Protection Regulation (GDPR). Influweb’s data collection process was reviewed and approved by the institutional review board of the ISI Foundation which waived the need for ethics approval as the following applies: all use takes place in compliance with the rules contained in the GDPR; informed consent was obtained online from all participants of the platform at enrollment according to regulations, enabling the collection, storage, and treatment of data, and their publication in anonymized, processed, and aggregated forms for scientific purposes; the website (https://influweb.org/) has a “Privacy Statement” section in which the users who decide to enroll in the study can find all the information on who is responsible for the data acquisition and processing in each country. To ensure privacy and data security, all collected data is pseudonymized—participants’ personal identifiers (e.g., email addresses) are stored separately and are inaccessible to researchers. Survey responses are linked only via an anonymized ID, preserving full privacy. Influweb operates on Google Cloud Platform (GCP) infrastructure in Europe, maintaining GDPR compliance, with continuous backups. Additionally, security measures to protect participant data include hashed passwords, two-factor authentication (2FA) and protection of data in transit by Transport Layer Security v1.3 (“TLSv1.3”). The additional questions that were added to the survey for the purpose of this study are fully consistent with the purposes previously communicated to the participants. Consequently, it was not deemed necessary to issue a new privacy notice and/or obtain additional consent from the participants nor obtain further ethical approval. We emphasize that responding to the new questions is also optional, and the publication of the survey results does not include personal data, but only aggregated and therefore anonymous data*.*

### 2.2. InfluenzaNet data collection

We collected cross-sectional data on knowledge, attitudes, and beliefs related to dengue fever among the French and Italian cohorts of InfluenzaNet, a survey network monitoring influenza-like illnesses across Europe [46]. Recruitment to the platforms Grippenet/Covidnet and Influweb is driven by several processes including word of mouth, communication campaigns, newspaper and radio interviews as well as social-media promotion. Following an intake survey covering socio-demographic and health-related characteristics, enrolled participants are sent out weekly questionnaires on their influenza-like symptoms. Participation in weekly questionnaires is voluntary. For this study, participants were invited to complete a dengue-specific questionnaire to assess their recent history of symptoms possibly linked to dengue, awareness of the disease, knowledge of key characteristics, concern levels, perception and adoption of preventive measures, and vaccination willingness, including reasons for refusal. All questions were mandatory, with some questions only asked to a relevant subset of the participants (i.e., knowledge of dengue characteristics was only asked to the participants who declared having heard of dengue before). The resulting variables were only analysed for the subset of the participants to whom the questions were asked, such that no missing data was handled. The full survey text is available in S1 Appendix.

### 2.3. Study period and inclusion criteria

Data were collected between May and October 2024. The invitation process depended on the platforms. In Italy, all registered participants to the platforms were invited by email to complete the questionnaire monthly from May 13, 2024, to October 27, 2024. These longitudinal data were intended to capture the impact of a potential summer outbreak, which will be the subject of a future study. For the present analysis, to avoid bias due to previous exposure to the questions, we included only the first complete submission of a questionnaire for each participant in Italy. In France, the questionnaire was available to all registered users from July 29, 2024, and registered participants to the platform's newsletter were invited to participate by email once, on the 29th of July 2024. We included responses submitted until August 6, 2024, after which the daily numbers of responses decreased significantly. Participants were able to submit multiple responses within this single survey wave, as long as the questionnaire remained open. French responses were post processed to associate each participant with a single response (see S1 Appendix). In both countries, we included participants who reported their sex, age and education level, provided that these met the age/sex/education census stratifications available for each country (see section *Post-Stratification*). We included residents of mainland France (including Corsica) and Italy. Included participants from the two countries were pooled for the analysis.

### 2.4. Post-stratification

The InfluenzaNet French and Italian samples differ from the general population in each country, with older age groups, and higher education typically over-represented [51]. To correct for this representativeness bias, we post-stratified the sample by age, sex, and education level. Each response was weighted to recover the national population characteristics, obtained through the French National Institute of Statistics and Economic Studies (INSEE) [52] and the Italian National Institute of Statistics (ISTAT) [53]. We considered three age groups (20–44, 45–59, and 60+ years), sex (male and female), and two education attainment levels based on the highest qualification achieved: ‘up to high school’ (including primary, secondary, and high school diplomas) and ‘post-high school’ (including any university studies or vocational training undertaken after high school).

### 2.5. Statistical analysis

We investigated the determinants of awareness of dengue as a disease, knowledge of its characteristics, adoption of preventive measures, and vaccination willingness. Additionally, we investigated the factors associated with the most common reasons for refusing vaccination if offered: believing oneself to be at low risk of contracting dengue, and not belonging to any risk group. We identified determinants with bias adjusted multivariate logistic and ordinal regression models [54], depending on the nature of the outcome. In particular, ordinal regression models were used to capture the ordered nature of knowledge levels and preventive measure adoption [55,56]. The post-stratification weights were included in the regression model as case weights, modifying the weight given to each respondent in the estimation of the likelihood (see S1 Appendix). Weighting was accounted for in the estimations of confidence intervals. We assessed the assumption of odds proportionality underlying these models in all instances where they were used [57,58] (see S1 Appendix, Fig C and D). Analyses were conducted on a single pooled dataset including both French and Italian participants. We assessed the homogeneity of the two samples as described in the section *Homogeneity of the two samples.* The statistical analysis was implemented in Python using the statsmodels library, together with the R library MASS, accessed in Python via rpy2.

### 2.6. Explanatory variables

Explanatory variables, summarised in Table 1, were derived from participants’ intake survey, and from responses to the dengue questionnaire. From the former, we obtained their country of residence (variable name: *Country*), sex (*Sex*), age (*Age group*), educational attainment (*Education level*), employment status (*Occupation*) and presence of at least one comorbidity (*Comorbidities*). From the zip code of residence of each participant, we determined the urbanicity of their domicile (*Urbanicity*) using data from ISTAT for Italy [59] and INSEE for France [60]. We also assigned to each participant a binary variable (*Autochthonous cases*) encoding whether autochthonous transmission of dengue had been reported in their department (France) or province (Italy) of residence during the year 2023 [61,62]. Dengue symptom history (*Symptoms*), dengue vaccination status (*Vaccination status*), search of information related to dengue (*Searched information*), knowledge of a case among contacts (*Known case*), level of concern related to dengue among different dimensions (*Worry level*), perceived efficacy of several preventive measures (*Efficacy score*) as well as knowledge of dengue characteristics (*Knowledge score*) and self-evaluated level of information about dengue (*Self-evaluation*) were all obtained from the survey replies.

**Table 1:** Explanatory variables, their levels and reference, as considered in the statistical analysis to explain knowledge, attitudes and practices related to dengue.

Variable	Values (reference in bold)
*Country*	**France**, Italy
*Sex*	**Male**, Female
*Age group*	20-44, 45-64, **65+**
*Education level*	Until high school, **Post high school**
*Occupation*	True, **False**
*Urbanicity*	**Urban**, Rural
*Symptoms*	True, **False**
*Comorbidities*	True, **False**
*Autochthonous case*	True, **False**
*Vaccination status*	True, **False**
*Searched information*	True, **False**
*Known case*	True, **False**
*Worry level*	Continuous variable
*Knowledge score*	Poor, Medium, Good, **Excellent**
*Efficacy score*	Continuous variable
*Self-evaluation*	Continuous variable

In order to capture the impact of a history of symptoms characteristic of dengue on knowledge, attitudes and preventive practices adoption, we defined the binary variable *Symptoms* to describe participants who had experienced at least two of the following symptoms in the past thirty days: “*eye pain; blood in the feces; blood in the vomit; bloody gums; bone pain”*. In order to maximise epidemiological specificity, non-specific symptoms of dengue such as fever, headache and cough were not considered due to the high prevalence of respiratory and gastrointestinal viruses with similar clinical manifestations. For sensitivity, we considered alternative definitions of the variable *Symptoms* based on the experience of at least one and at least three symptoms in the past thirty days. The *Vaccination status* was ascertained through a dedicated question in the survey “*Did you get the vaccine against Dengue?*”. As the dengue vaccine is highly specific and non-routine in France and Italy, individuals who received the vaccine would have received it in a highly specific context, and are likely to recall it accurately.

We measured *Worry level* as the average of the participants’ concern level across five dimensions, each rated on a 5 points Likert scale from 1 (*Not at all worried*) to 5 (*Extremely worried*). Dimensions included i) fear of being infected, ii) fear of having severe symptoms if sick, iii) fear for friends/family, iv) fear for work/economic consequences in case of contagion, v) fear of work/economic consequences in the event of a pandemic. The *Efficacy score* of a participant was instead computed by averaging their rating of the efficacy of the various measures proposed. Finally, *Self-evaluation* refers to each participant’s personal perceived information level, rated on a scale from 1 (*Very poor or none at all*) to 5 (*Very accurate*).

Participants who were aware of dengue were assigned a *knowledge score* based on the number of known characteristics among the eight presented: Poor (0–2), Medium (3–4), Good (5–6), and Excellent (7–8). The dengue characteristics we enquired about were i) “*Dengue is transmitted through bites of infected A. albopictus (tiger mosquito) or A. aegypti mosquitoes”*, ii) “*Mild cases of dengue have symptoms similar to Covid-19 or the Flu”, iii) “Dengue can be asymptomatic”*, iv) “*Symptoms last on average between 2 and 7 days”*, v) “*Severe cases of dengue can lead to hospitalization and death”*, vi) “*There is no specific treatment or medicine against dengue”*, vii) *“If you have already had dengue in the past, you are more at risk of having severe symptoms”*, viii) “*Pregnant women and children are more at risk of having severe symptoms”*. The variable *knowledge score* was also considered as an outcome variable.

Internal consistency for the reflective composite scales was assessed using weighted Cronbach's alpha, yielding coefficients of 0.915 for the *Worry level* and 0.814 for the *Efficacy score*. Although the *Knowledge score* was designed as a formative cumulative index, we also evaluated its internal consistency, which yielded an acceptable weighted alpha of 0.773. These composite variables were only defined for participants who had heard of dengue. All questions used to build the composite scores were mandatory for these participants. These variables entered the analysis as outcome variables and/or explanatory variables in regression models restricted to the sample of participants who had heard of dengue.

### 2.7. Outcome variables

#### 2.7.1. Awareness of dengue as a disease.

Participants were initially asked whether they were “*aware of the existence of an infectious disease called dengue in your country or around the world*”. This outcome was modelled with a bias adjusted logistic regression model, with backward stepwise variable selection (see section *Variable selection*). We included the following variables as predictors prior to variable selection: *Country*, *Sex*, *Age group, Education level*, *Occupation, Urbanicity*, *Symptoms*, *Comorbidities*, and *Autochthonous case*.

#### 2.7.2. Knowledge of dengue.

We modelled the participants’ *knowledge score* (see section *Explanatory variables)* with a bias adjusted ordinal regression model, with backward stepwise variable selection (see section *Variable selection*). We included the following variables as predictors prior to variable selection: *Country, Sex, Age group, Education level, Occupation, Urbanicity, Symptoms, Comorbidities, Vaccination status, Searched information, Known case, Worry level and Autochthonous case.*

#### 2.7.3. Adoption of preventive behaviours.

We enquired about 6 specific preventive behaviours known to reduce the risk of contracting dengue [63]: i) “*Wearing long-sleeved clothing covering arms and legs”*, ii) “*Regularly using mosquito repellent”*, iii) “*Avoiding swampy areas or areas with a high density of mosquitoes”*, iv) “*Avoiding traveling/canceling a trip to areas at risk”*, v) “*Installing mosquito nets in all windows”*, vi) “*Removing all stagnant water from the balcony, garden, and windows”*. We considered the total number of adopted measures as an indicator of participants’ prevention practices regarding dengue, binning it in four categories: “poor adoption” (0 measures), “medium adoption” (1–2 measures), “good adoption” (3–4 measures) and “excellent adoption” (5–6 measures).

This outcome was modelled with a bias adjusted ordinal regression model, with backward stepwise variable selection (see section *Variable selection*). We included the following variables as predictors prior to variable selection: *Country, Sex, Age group, Education level, Occupation, Urbanicity, Comorbidities, Vaccination status, Searched information, Known case, Worry level, Knowledge score, Efficacy score and Autochthonous case*.

#### 2.7.4. Attitudes towards vaccination.

Non-vaccinated participants who had heard of dengue were asked whether they would accept a vaccine if offered. Unwilling participants were asked to specify their reasons for refusal from the following options: *“I don't belong to any risk group; It is better to build your immune defences naturally; I have doubts about the effectiveness of the vaccine; I don't think I'll get dengue; I am concerned that the vaccine is unsafe or may cause illness or side effects; I don't like receiving vaccinations; The vaccine is currently too expensive; My doctor advised me against getting vaccinated; None of the above (Please, specify the reason)”*. We analysed the determinants of vaccination willingness among unvaccinated participants, and the determinants of the two equally most commonly reported reasons for refusing vaccination—“*I don’t think I will get dengue*,” and “*I don't belong to any risk group*”.

These outcomes were modelled with bias adjusted logistic regression models, with backward stepwise variable selection (see section *Variable selection*). We included the following variables as predictors prior to variable selection: *Country, Sex, Age group, Education level, Occupation, Urbanicity, Symptoms, Comorbidities, Searched information, Known case, Worry level, Knowledge score, Self-evaluation and Autochthonous case*.

### 2.8. Variable selection

For each outcome considered, explanatory variables included in the final models were obtained with a stepwise backward selection process [32,64,65]. Each variable was initially tested in a univariate analysis, and those with a p-value below 0.2 were retained for the multivariate analysis. The least significant variable (highest p-value) was iteratively removed until all remaining predictors had a p-value ≤ 0.05. For categorical variables, the selection was based on the smallest p-value among its levels. This approach, based on p-values, allows us to identify the most parsimonious set of predictors. In the final models, the strength and direction of the relationships between predictors and outcomes are instead assessed, and our model interpretations are based on both statistical significance and effect sizes/directionality. For sensitivity, we considered simultaneously all possible multivariate models for each outcome, and compared the variables selected by the approach described above with those included in the model with highest adjusted pseudo R^2^ (see S1 Appendix). Moreover, we assessed the sensitivity of the model results to alternative model specifications, by considering logistic/ordinal regression models including all explanatory variables without any variable selection, and alternative regression models with a Ridge regularisation term (proportional to the L2 norm) for the binary outcomes (see S1 Appendix, Fig A and B, and Table B, C, D, and E).

### 2.9. Homogeneity of the two samples

We conducted the statistical analysis on the pooled dataset merging participants from both France and Italy. While we controlled for country of residence by including it as a predictor, the analysis in the absence of interaction term assumes that the effects of the remaining predictors are the same in both countries. For each predictor we tested whether the country of residence had a modulating effect using simple models including interaction terms. For each outcome, we considered a set of logistic or ordinal regression models with the country of residence, the predictor tested for interaction, and their interaction terms. Where the interaction term was significant, we presented the odds ratios separately for the French and Italian cohorts to characterise the effect of the predictor. Where the interaction term was not significant, we concluded that the country of residence had no impact on the effect of the predictor tested, and the two samples were thus homogeneous. The results of this analysis are shown in the section *Interaction Terms* of the S1 Appendix (Fig E, F, G, H, and I).

## 3. Results

### 3.1. Descriptive analysis

#### 3.1.1. Participants.

All users registered in the Grippenet/Covidnet and InfluWeb platforms at the time of the study could fill the survey (respectively 6252 and 994 users in Grippenet/Covidnet and InfluWeb). Of the users active in the year prior to the study (4856 and 585 users in each platform), a total of 2719 and 436 users responded to the survey in France and Italy respectively, with a response rate of 56% and 69% (based on active users in the year prior to the study). A total of 2500 and 404 responses were considered after data cleaning, and analysed further.

In France, included participants filled in the questionnaire between July 29th and August 6th; with a majority (57%) filled in the first 4 days of data collection (until August 1st). Responses obtained in Italy were instead gathered over a longer timespan, between May and October 2024. The majority of the responses included (85%) were received before the end of June 2024. Socio-demographic characteristics of these participants, before and after bias adjustment, are shown in Table 2. Male individuals were overrepresented in Italy, whereas the French sample was skewed toward females. While more than half of the French participants were above 65 years old (55%), the most common age group among participants from Italy was “45-64” (51.7% participants). The youngest age group “20-44” was the least represented in both countries. Participants from Italy were nearly equally likely to have completed pre- or post-high school education. In France instead, the majority of the participants (72%) had educational attainment beyond high school. In order to correct this bias in sex, age and educational attainment, we applied post-stratification weights to the samples. Adjusted characteristics of the two samples are shown in Table 2, in the form of percentages. While this process improves the representativeness of the samples, it cannot correct for residual selection bias driven by factors not considered in the stratification.

**Table 2:** Characteristics of the participants sampled. We report raw numbers with their percentage referring to participants in the survey. Percentages in the “Adjusted” columns were obtained with post-stratification adjustment weights.

Variable	Level	Italy		France		Total	
		Raw	Adjusted	Raw	Adjusted	Raw	Adjusted
**Sex**	Male	220 (54.5%)	48.1%	1060 (42.4%)	47.5%	1280 (44.1%)	47.6%
	Female	184 (45.5%)	51.9%	1440 (57.6%)	52.5%	1624 (55.9%)	52.4%
**Age group**	20-44	78 (19.3%)	35.1%	230 (9.2%)	37.0%	308 (10.6%)	36.8%
	45-64	209 (51.7%)	37.0%	894 (35.8%)	35.7%	1103 (38.0%)	35.9%
	65+	117 (29.0%)	27.9%	1376 (55.0%)	27.3%	1493 (51.4%)	27.4%
**Education level**	Until high school	196 (48.5%)	83.8%	700 (28.0%)	69.0%	896 (30.9%)	71.1%
	Post high school	208 (51.5%)	16.2%	1800 (72.0%)	31.0%	2008 (69.1%)	28.9%
**Occupation**	Yes	249 (61.6%)	54.6%	963 (38.5%)	54.2%	1212 (41.7%)	54.3%
	No	155 (38.4%)	45.4%	1537 (61.5%)	45.8%	1692 (58.3%)	45.7%
**Urbanicity**	Rural	62 (15.3%)	15.6%	677 (27.1%)	28.2%	739 (25.4%)	26.4%
	Urban	342 (84.7%)	84.4%	1823 (72.9%)	71.8%	2165 (74.6%)	73.6%
**Dengue Awareness**	Yes	356 (88.1%)	84.2%	2353 (94.1%)	92.3%	2709 (93.3%)	91.2%
	No	48 (11.9%)	15.8%	147 (5.9%)	7.7%	195 (6.7%)	8.8%

The remainder of our analysis is conducted on a pooled sample including both sets of participants, unless otherwise specified. All proportions quoted in the rest of this article refer to the adjusted proportions after post-stratification weighting of the samples. We refer to the Table A of the S1 Appendix for a comparison of the sample characteristics to the users active on the platform in the year prior to the study who did not fill in the survey.

#### 3.1.2. Symptom history and dengue prevalence.

The most commonly reported symptom was eye pain (4.93% of the participants who filled the survey), followed by bone pain (4.35%), bloody gums (3.27%), blood in the feces (1.75%) and blood in the vomit (0.19%). Overall, 10.19% of the participants reported experiencing one or more symptoms in the past thirty days, 4.11% reported at least two symptoms, and 0.19% reported experiencing three or more symptoms. No participants were diagnosed with dengue in the past 30 days.

#### 3.1.3. Awareness of dengue, and knowledge of its characteristics.

**Fig 1:**
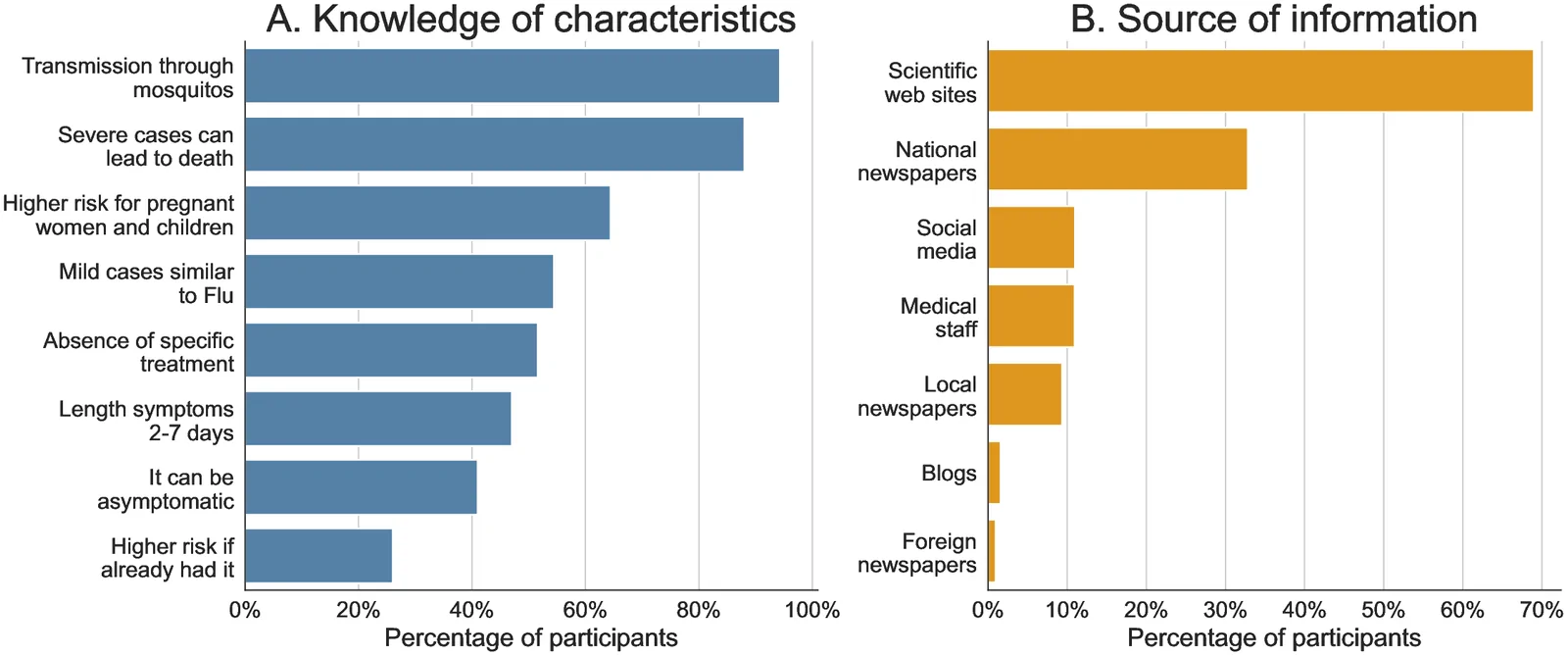
Knowledge among the participants. A) Percentage of participants (in the French and Italian samples) knowing each characteristic of dengue. B) Percentage of participants consulting each source of information. The percentages computed in panel A are with respect to the participants who have heard of dengue (N = 2709). In panel B, the proportions computed are among those participants that have actively sought information on the spread of dengue in their country (N = 419). Percentages are computed using the joint French and Italian samples adjusted with post-stratification weights to recover representative samples in terms of age distribution, sex and education in each country.

The vast majority of respondents (91.2% in the combined sample, after adjustment) had heard of dengue as a disease. This awareness was slightly higher in France (92.3%) than in Italy (84.2%). Knowledge levels however varied widely across different characteristics of dengue (see Fig 1A). While 94.3% of participants recognised that “*dengue is transmitted through the bites of infected A. albopictus (tiger mosquito) or A. aegypti mosquitoes*”, and 88.0% of participants knew that “*Severe cases of dengue can lead to hospitalization and death*”, only 41.0% of respondents were aware that “*dengue can be asymptomatic*”, and just 26.0% knew that “*If you have already had dengue in the past, you are more at risk of developing severe symptoms*”.

A minority (14.6%) of participants had actively looked for information on the spread of dengue in their country, consulting the sources shown in Fig 1B. The most frequently used source was “*Scientific information and dissemination websites*” (69.0% of the participants who had searched for information), followed by “*National newspapers*” (32.8%).

#### 3.1.4. Adoption and perceived efficacy of preventive measures.

**Fig 2:**
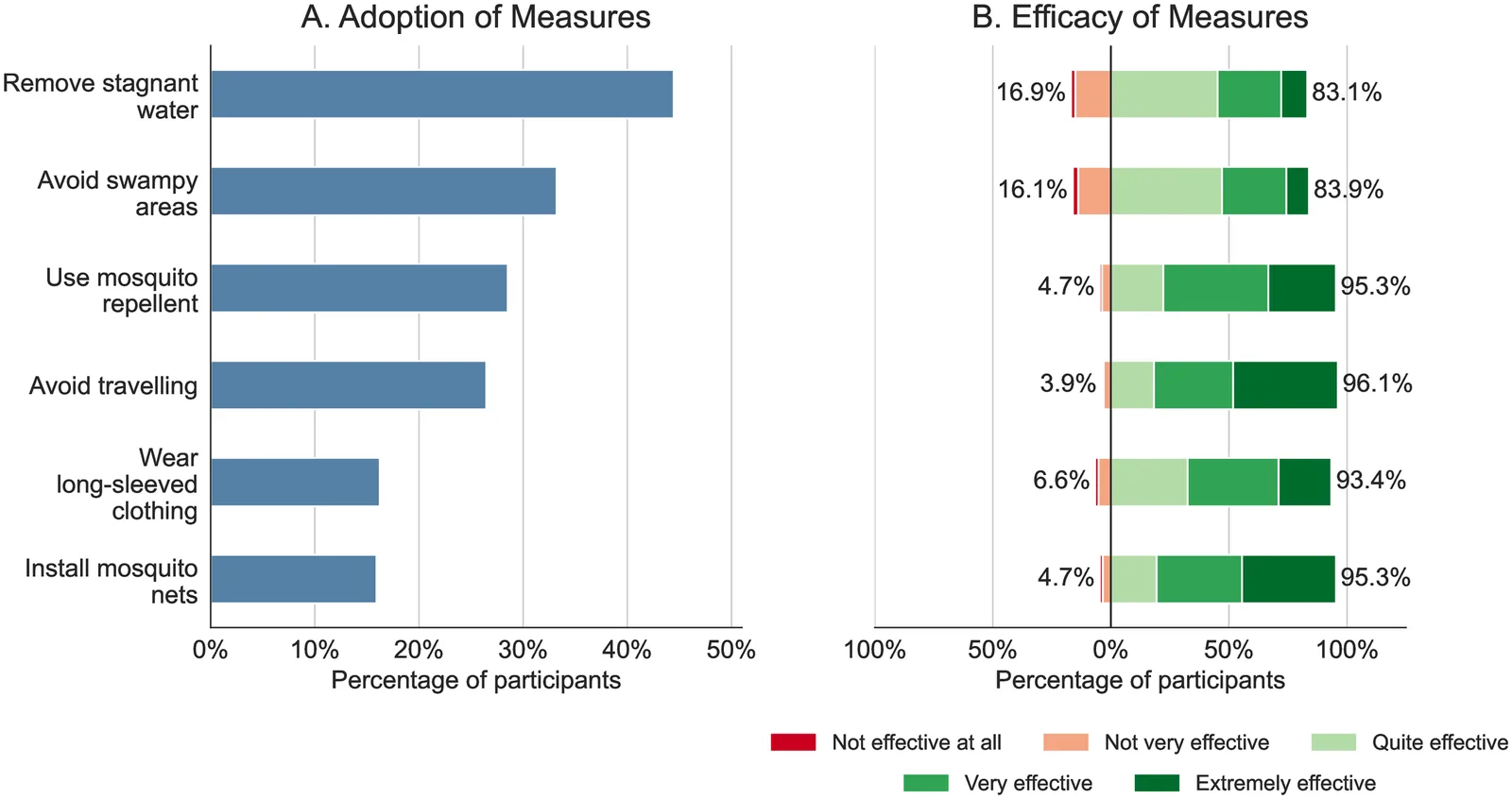
Adoption and perception of preventive measures. A) Percentage of participants adopting each preventive measure. B) Distributions of perceived efficacy of each measure rated by the participants. In both panels, percentages are computed with respect to the participants who have heard of dengue (N = 2709), using the joint French and Italian samples adjusted with post-stratification weights to recover representative samples in terms of age distribution, sex and education in each country.

The most commonly adopted measure was “*Removing stagnant water from balconies, windows, and gardens*” (44.4%), followed by “*Avoiding swampy areas or areas with a high density of mosquitoes*” (33.2%) (see Fig 2A). In contrast, “*Wearing long-sleeved clothing that covers arms and legs*” and “*Installing mosquito nets on windows*” were adopted by only 16.2% and 15.9% of the participants, respectively. Perceived efficacy of each measure is shown in Fig 2B. All six measures were generally considered effective, with over 80% of respondents rating each measure as at least “quite effective.” For four measures, this percentage exceeded 93%. Interestingly, the two most adopted measures were also perceived as the least effective.

#### 3.1.5. Fears and vaccination.

**Fig 3:**
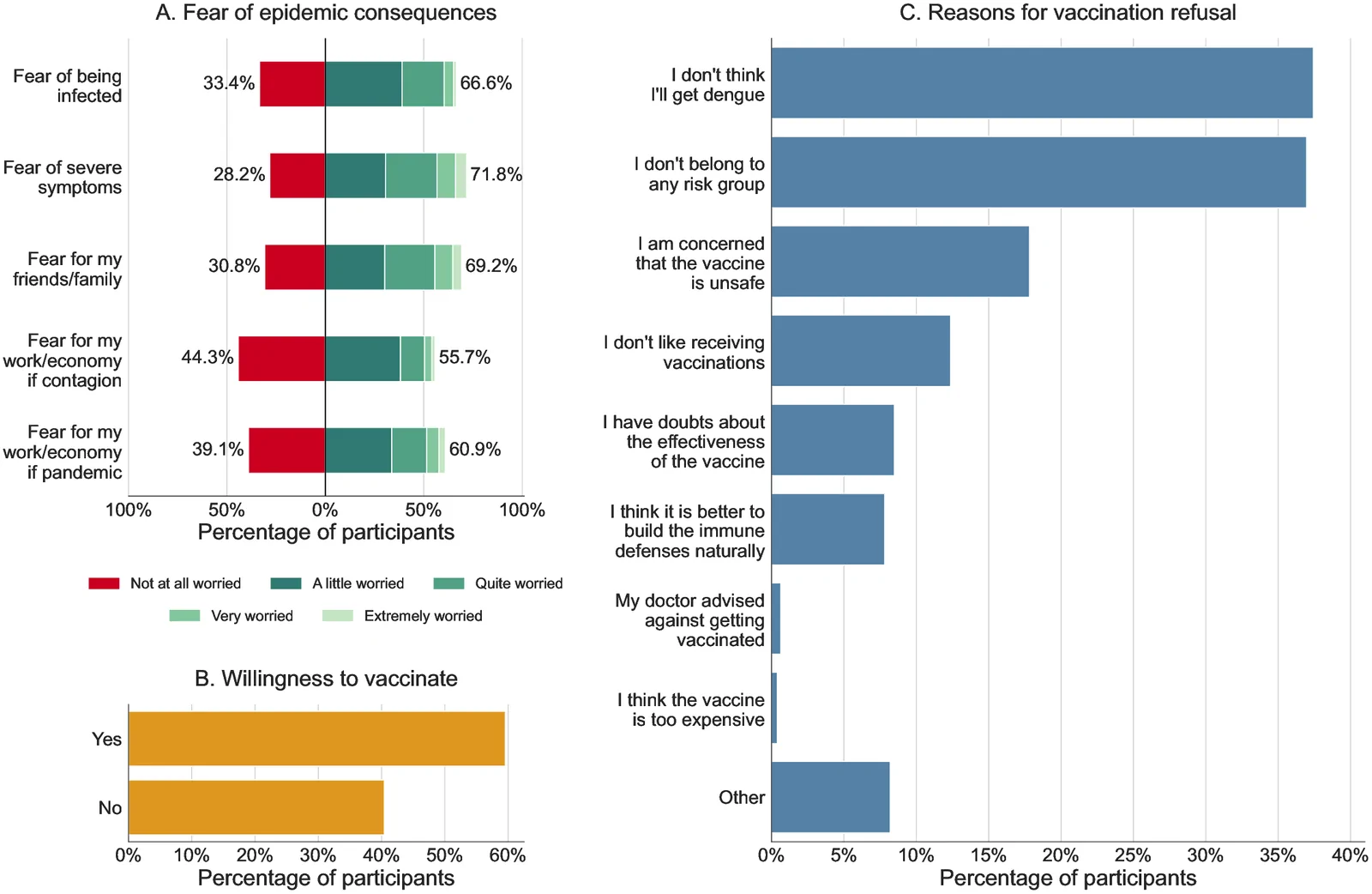
Sentiments towards dengue epidemics and vaccination against dengue. A) Distributions of concern levels for each consequence of a hypothetical dengue epidemic. B) Percentage of participants willing to accept a vaccine if offered. C) Percentage of unwilling participants citing each reason for refusal. In panel A, percentages are computed with respect to the participants who have heard of dengue (N = 2709). In panel B, percentages are computed with respect to the participants who have heard of dengue and are unvaccinated (N = 2698) while instead percentages reported in panel C are computed with respect to the subset of participants who are unwilling to accept a vaccine (N = 1117). All results shown are obtained using the joint French and Italian samples adjusted with post-stratification weights to recover representative samples in terms of age distribution, sex and education in each country.

We observed moderate levels of concerns among the population sampled (see Fig 3A). Concern levels varied slightly depending on consequences of an epidemic. Participants expressed greater concern about severe symptoms and the well-being of friends and family, as indicated by the high percentage of responses in the top three levels of worry. In contrast, work- and economy-related fears —both in the event of infection and in the broader context of an outbreak— showed lower levels of concern. For instance, only 28.2% of participants expressed no concern for “*Fear of having severe symptoms if I get sick*” while 44.3% of participants had no concern for “*Fear of work/economic consequences in case of contagion*.”

Among the 2,709 respondents who had heard of dengue, only 11 had been vaccinated against it. Instead, 59.4% of unvaccinated participants would be willing to receive a vaccine if offered (see Fig 3B). As shown in Fig 3C, the most frequently cited reasons to refuse a vaccine if offered were “*I don’t think I’ll get dengue*”, cited by 37.4% of unwilling participants; and “*I don’t belong to any risk group*”, cited by 37.0%.

### 3.2. Determinants of knowledge, behaviours and attitudes

**Fig 4:**
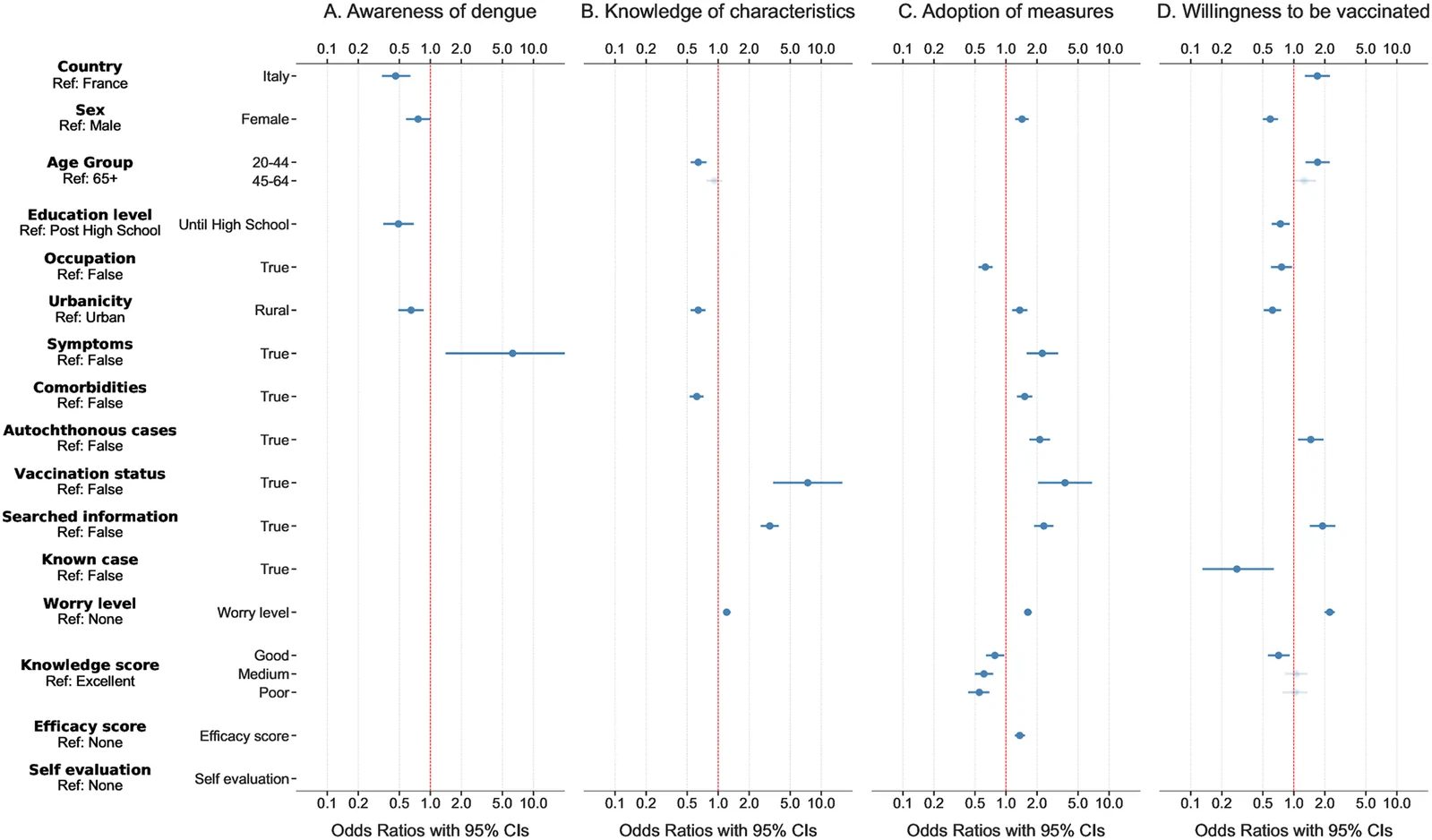
Determinants of knowledge of dengue, its characteristics, adoption of preventive measures, and vaccination willingness. A) Determinants of the awareness of dengue identified with a logistic regression model in the form of odds ratios with 95% confidence intervals. B) Determinants of the knowledge of dengue characteristics, obtained with an ordinal regression model applied to the answers of the participants who have heard of dengue. C) Determinants associated with adoption of preventive numbers (obtained from an ordinal regression model, among participants who have heard of dengue). D) Determinants of willingness to be vaccinated (binary logistic model) among the participants who are unvaccinated. Models are multivariate, with variables selected as described in the Methods section. Variables without an associated OR for a given outcome were either not included as explanatory variables in the initial list of considered determinants, or were excluded during the variable selection process. Faded points represent non-significant levels of included variables. All analyses are conducted on the pooled dataset including French and Italian samples.

Determinants of the awareness of dengue as a disease, knowledge of its characteristics, adoption of preventive measures, and willingness to be vaccinated are shown in Fig 4. We consider adjusted odds ratios (ORs) obtained using multivariate logistic or ordinal regression models fitted to the joint samples, with post-stratification (case weights) adjusting for age, sex and education distributions.

#### 3.2.1. Awareness of dengue.

Our logistic regression model for awareness of “*the existence of an infectious disease called dengue*” identified sex, country of residence, urbanicity, educational attainment, and symptom history as drivers for awareness (see Fig 4A). Being female (OR 0.76), living in Italy (OR 0.46), living in a rural area (OR 0.65) and low educational attainment (OR 0.49) were all characteristics associated with lower awareness. Instead, participants who had experienced at least two of the five considered symptoms possibly linked to dengue in the past thirty days were more likely to have heard of the disease (OR 6.29). We found no associations between awareness of the disease and presence of comorbidities, occupation, age group, or living in a department/province with reported locally acquired dengue cases in the past year. The symptom history variable becomes statistically non-significant when considering alternative definitions based on one or more symptoms, or three or more symptoms (respectively). The remainder of the determinants identified by this model are robust to these changes (see S1 Appendix, Fig J and Fig L).

#### 3.2.2. Knowledge of characteristics.

Drivers associated with better knowledge of dengue characteristics, among the participants who had heard of dengue as a disease (N = 2709), are shown in Fig 4B. Knowledge appeared associated in particular with information seeking behaviour (OR 3.17), concern levels (OR 1.21), and previous vaccination against dengue (OR 7.4). Urbanicity also played a role, with participants in rural areas having lower odds of high knowledge levels (OR 0.64) with respect to urban residents. Knowledge instead was not significantly determined by participants’ country of residence, sex, educational attainment, occupation, symptom history, knowledge of a dengue case or living in an area with autochthonous transmission in 2023. These results are robust to using an alternative definition of the symptom history based on at least three symptoms. Considering a more relaxed definition based on one or more symptoms, we find that the symptom history becomes statistically significant (negative association with knowledge), but the remainder of the determinants remain unchanged (see S1 Appendix, Fig J and L).

#### 3.2.3. Adoption of measures.

Adoption of preventive measures was associated with a combination of behavioural and socio-demographic determinants (see Fig 4C). Perceived efficacy of the measures (OR 1.36), concern levels (OR 1.63), and having actively sought information (OR 2.33) were all associated with higher odds of adopting preventive measures. Individuals with “good”, “medium” and “poor” knowledge all had lower odds of adopting preventive measures (OR = 0.78, OR = 0.61, and OR = 0.55, respectively) compared to individuals with “excellent” knowledge. The local context was also associated with the adoption of preventive measures. In particular, residing in a rural area (OR = 1.36) and living in a province/department with previous local transmission of dengue (OR = 2.13) were both associated with higher odds of adopting preventive measures. Female participants had 1.43 times the odds of adopting a larger number of measures than men. Previous vaccination and dengue symptom history were also positively associated with higher adoption of measures. Being employed had the opposite effect, and was associated with lower odds of adopting a larger number of measures (OR = 0.63). Adoption of measures however did not significantly depend on country of residence, educational attainment, and knowledge of a case of dengue among contacts. The symptom history variable becomes statistically non-significant when considering alternative definitions based on one or more symptoms, or three or more symptoms (respectively). The remainder of the determinants identified by this model are robust to these changes (see S1 Appendix, Fig J and L).

#### 3.2.4. Willingness to vaccinate.

Focusing on unvaccinated participants (N = 2698), we identified determinants associated with willingness to accept a vaccine if offered (see Fig 4D). Socio-demographic and contextual characteristics were especially associated with willingness. Higher willingness was associated with residing in Italy (OR 1.69), the 20–44 age group (OR 1.70), and areas where locally acquired cases of dengue were reported in 2023 (OR 1.46). Instead, female sex (OR 0.59), educational attainment limited to high school (OR 0.74), having an occupation (OR 0.76), and living in rural areas (OR 0.62) were associated with lower willingness. Behaviours and attitudes were also correlated to willingness: information seeking (OR 1.90), and increased concerns (OR 2.23) were associated with higher willingness. Willingness was independent of participants’ own perceived level of information, comorbidities and symptom history. These results are unaffected by considering alternative symptom history defined from one or more symptoms, or three or more symptoms (see S1 Appendix, Fig J and L).

#### 3.2.5. Reasons for vaccination refusal.

**Fig 5:**
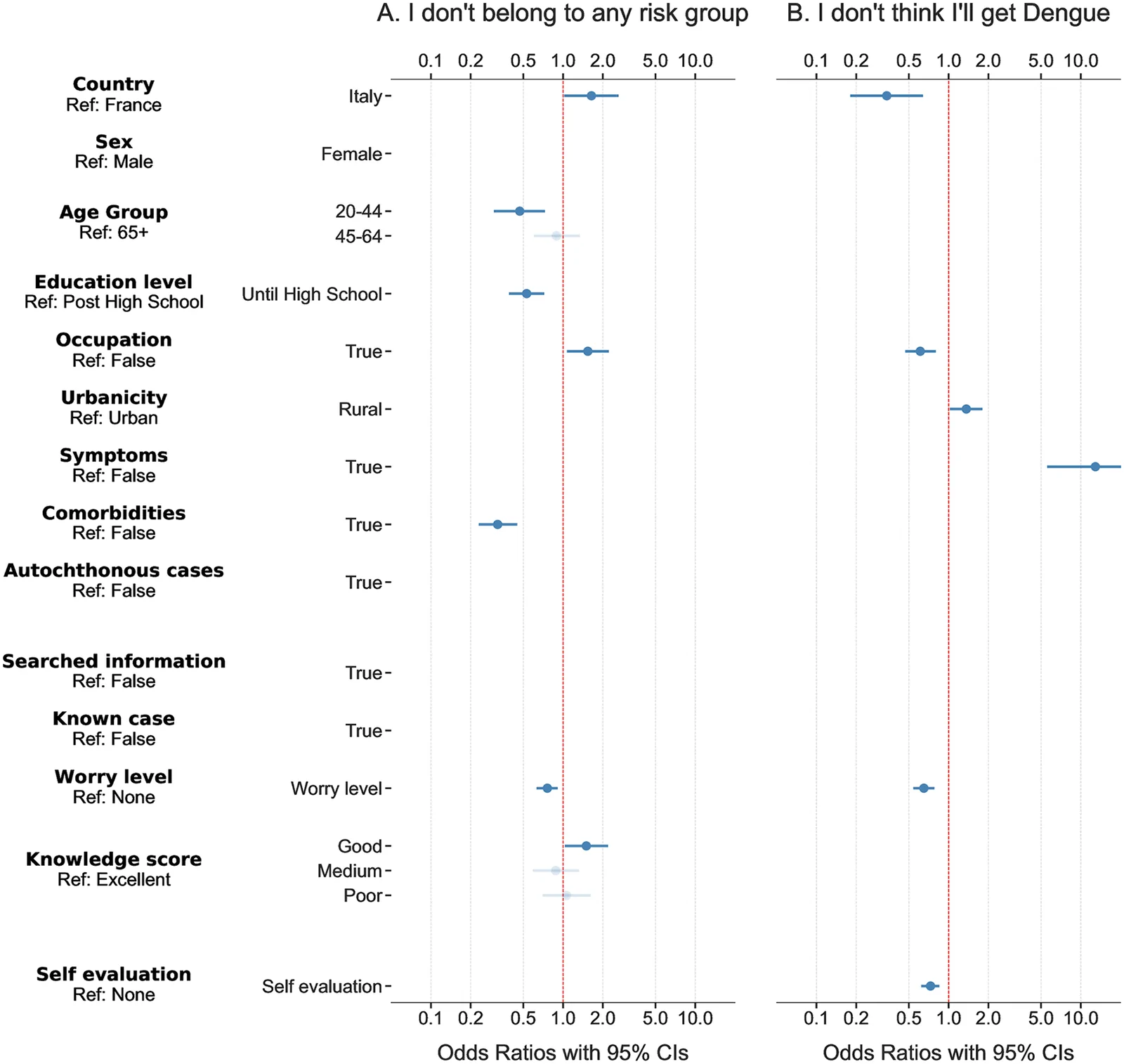
Determinants of the main reasons for vaccine refusal. A) Determinants of citing “I don’t belong to any risk group” in the form of odds ratios with 95% confidence intervals. B) Determinants of citing “I don’t think I’ll get dengue” in the form of odds ratios with 95% confidence intervals. Odds ratios were obtained from a multivariate, bias adjusted logistic regression model applied to the participants who are unwilling to be vaccinated. Variables without an associated OR were excluded during the variable selection process. Faded points represent non-significant levels of included variables. All analyses are conducted on the pooled dataset including French and Italian samples.

We identified determinants explaining the most commonly cited reasons for vaccine refusal among unwilling participants (N = 1117) (see Fig 5). Both *“I don’t think I will get dengue”* and *“I don’t belong to any risk groups”* were less likely to be cited by participants with higher concerns (OR 0.65 and 0.76 respectively). Residing in Italy (OR 1.64), having an occupation (OR 1.54), and having a “good” (compared to “excellent”) knowledge of dengue characteristics (OR 1.50) showed higher odds of refusal with “*I don’t belong to any risk group*”. Instead, the 20–44 age group (OR 0.47), educational attainment limited to high school (OR 0.53), and comorbidities (OR 0.32) were associated with lower odds of citing this reason. Italian residents (OR 0.34), those with an occupation (OR 0.61), and those perceiving themselves more highly informed (OR 0.73) had lower odds to quote *“I don’t think I'll get dengue*”, while residing in urban areas instead increased the odds of citing this reason (OR 1.36). The remaining of the variables represented in Fig 5 without an associated OR were not found to be significant by our analysis. Considering alternative definitions of the symptom history based on one or more symptoms or three or more symptoms, the association of the symptom history with the outcome *“I don’t think I will get dengue”* becomes non-significant. Moreover, the statistical significance of the determinants close to the significance threshold differs for the same outcome when the symptom history definition changes. In particular, the “20-44” class of the variable “*Age group*” (previously non-significant) becomes significant (positive association) when symptom history is based on one or more symptoms. The variable “*Urbanicity*” (previously significant) becomes statistically non-significant when either alternative definitions are considered, and the variable “*Known case*” reaches significance when the symptom history is based on three or more symptoms. No reversal of effects are observed, and the remaining determinants identified by the two models are robust to changes in the definition of the symptom history (see S1 Appendix, Fig K and M).

#### 3.2.6. Interactions between the country of residence and the identified determinants.

We refer to the S1 Appendix (Fig E, F, G, H, and I) for results of the bivariate regression analyses assessing the homogeneity of the French and Italian samples. In the majority of the determinants considered, interaction terms with the country of residence were not significant, indicating that the determinant's effects apply independently of countries. Where interaction terms were statistically significant, the main effect of country of residence (i.e., the Italian dummy variable) often became non-significant. This may be due to the fact that the independent effect of the country was absorbed into the specific interaction terms, indicating that the effect of those predictors was not independent of country but rather country-specific.

Significant interactions were identified in a few cases, highlighting few cross-country differences (see S1 Appendix, Fig E, F, G, H, and I). Among Italian participants, those with an occupation were less likely to know a higher number of characteristics than those without, whereas in France employment status did not correlate with knowledge. While the main analysis shows that urbanicity was negatively associated with adopted measures, this reversed in Italy. A similar effect was measured in the case of the association between areas where autochthonous cases had been reported, and willingness to be vaccinated. The first effect may reflect structural differences in the rural–urban context between the two countries, while the second could be due to the small number of distinct areas with autochthonous cases in Italy in 2023. Finally, while sex was not associated with “*I don’t think I'll get dengue*” in the joint analysis, female participants were less likely than males to report this reason in France, but instead more likely in Italy.

#### 3.2.7. Variance inflation factor.

As detailed in S1 Appendix, Table F and G, all explanatory variables included in the final models were tested for multicollinearity using the Variance Inflation Factor (VIF). All VIF values were below 3, with the exception of three variables (3 < values < 10), indicating that multicollinearity was not a concern in our analyses.

## 4. Discussion

Using French and Italian participatory surveillance cohorts of the European network InfluenzaNet [46], we gathered data on knowledge, attitudes and preventive practices related to dengue in two countries where dengue is an emerging threat. Interestingly, we found high awareness, moderate levels of concerns, and a moderate vaccination willingness. Knowledge and attitudes, socio-economic factors and the context were associated with the adoption of preventive measures, suggesting targeted approaches to increase adoption. Moreover, this study shows how pre-existing participatory surveillance cohorts can be pivoted towards emerging threats to assess knowledge, awareness and attitudes, as well as to evaluate determinants of knowledge, behaviours, and attitudes towards vaccination in order to help design efficient interventions.

Several surveys assessed dengue awareness and knowledge in non-endemic regions since the first detections of autochthonous cases [34,36]. In mainland France, successive survey waves probed the population between 2012 and 2014, finding increased knowledge that dengue can be transmitted by mosquitoes (from 58.5% in 2012 to 76.2% in 2014), but stable perceptions of severity and vulnerability [39,40]. Evidence from the French public health agency (Santé Publique France) however found that only 28.2% of the population surveyed in metropolitan areas with *A. albopictus* presence knew that mosquitoes around them could transmit dengue [38], suggesting that knowledge may not translate to risk and susceptibility awareness. In the Lazio region of Italy, while 49.8% of Italian participants knew that tiger mosquitoes could transmit “some diseases”, only 1% was able to correctly identify which ones in 2016. This reached 28.7% among Lazio residents originally from Kerala (South India) where dengue is endemic, showing wide gaps in knowledge between communities, and the crucial impact of the social and cultural context [37]. Similarly, we noted an association between vaccination status and knowledge in our analysis. As vaccination is not currently recommended to the general population, vaccinated individuals included in the French sample might have received the vaccine while residing in overseas departments where dengue is endemic, which may have shaped their knowledge. Finally, our analysis conducted on a sample collected in 2024 in Italy and France suggests higher awareness levels about the existence of dengue (91.2%) and higher knowledge of its transmission mode (94.3%), possibly due to timings of data collection, and increasing reports of local transmissions since previous surveys were implemented.

Our survey focused on the knowledge of characteristics specific to dengue, while existing research was typically centred on knowledge related to mosquitoes and the diseases they carry [36,37,39–41]. Heterogeneous knowledge of symptoms of mosquito-borne viruses has been previously noted in France in 2012 [40]. Our study found persistent heterogeneity in knowledge of dengue characteristics in 2024: the transmission mode of dengue was known by 94.3%, while only 26.0% of participants were aware of the increased risk associated with reinfection, and 41.0% knew that dengue could be asymptomatic. This shows that, while some features of dengue are highly known in non-endemic regions, some clinical signs of the disease and risk factors remain poorly assessed by the population surveyed. This highlights an urgent need to implement communication campaigns to raise knowledge and awareness of these lesser known characteristics.

In particular, efforts should focus on the younger population, rural communities, and less educated groups, which were less likely to have heard of the disease, or know its characteristics. In the rural setting, where dengue literacy is lower, but preventive measures are more likely to be adopted, targeted information campaigns should leverage local organisations and communication channels to raise awareness and further enhance measure adoption. For example, interventions in local schools could help bridge this gap, by raising knowledge in younger individuals. Similarly, visual information campaigns in frequently visited locations (supermarket, health clinics, post office, public transportation hub) and audio-visual campaigns in local newspapers, radio and TV channels would help achieve an equitable reach across different groups. Moreover, the lower knowledge of characteristics among those with comorbidities suggests that information campaigns in medical establishments, often attended by this subpopulation, could be beneficial. Increasing knowledge in the population is particularly important, as higher knowledge is known to be associated with the adoption of preventive measures [32,35,41]. This is also the case in our sample, confirming that information campaigns raising knowledge could increase preventive measure adoption, and thus lead to better vector control.

Understanding information source preferences can suggest specific communication channels that can be used to raise awareness, improve knowledge and encourage the adoption of preventive practices. Our survey identified sources used by the 14.6% of sampled individuals actively searching for information on the circulation of dengue. Using these identified channels (scientific websites and national newspapers) provides a way to further inform those engaged individuals. However, future information campaigns would have the most impact when targeting individuals who do not look for information proactively, since information search was associated with higher knowledge, adoption of preventive behaviours, and willingness to vaccinate. In Spain and the Netherlands, 93.7% and 81.6% of participants were interested in receiving information on mosquitoes and mosquito-borne viruses [41]. Preferred sources for receiving this information however varied between countries, with Spanish participants preferring health professional information sources, and Dutch participants preferring government sources and websites [41]. Further research must be dedicated to identify targeted communication channels in the European context in order to ensure equitable reach of information campaigns across different socio-demographic groups (e.g., age, education level) and local contexts (e.g., country, urbanicity).

Our findings suggest a higher trend in the adoption of preventive practices compared to previous survey results from the past decade. After the first detection of locally transmitted dengue cases in Europe in 2012, approximately 50% of participants surveyed in France were adopting at least one recommended preventive measure [40]. Similarly, 47% of Italians surveyed in 2016 reported adopting precautions against tiger mosquitoes [37]. More than a decade later, our survey shows that the adoption of at least one preventive measure in France and Italy has increased to 59%. This increasing trend can be especially observed for eliminating standing water which was adopted by 17.7% of participants in 2012 [40], and 44.4% in our study, and repellent use which was adopted by 17.4% of participants in 2012 [40], 20.3% in 2016 [38] and 28.5% in our study in 2024. Use of mosquito nets and long sleeved clothing however remained stable since 2016, with 17.9% of participants reporting using nets often in 2016 [38], close to the 15.9% of the participants in our study. Similarly, long sleeved clothing was used often by 14.1% of the participants in 2016 [38] compared to 16.2% in our study. This increasing trend may also partly result from seasonal effects: our survey was conducted during the peak of vector activity in Europe, similarly to [37], while results presented in [40] were obtained from answers collected over half a year, and the survey from [38] was conducted during winter months. Our sample was collected over a short time span, compared to outbreak development and seasonal variations. The timing of the data collection may have heightened participants’ perceived risk and led to higher adoption of preventive measures, particularly for measures like repellent use or eliminating standing water. Future research and dedicated data collection are needed to explicitly reveal the impact of seasonality and concurrent outbreaks on the adoption of preventive behaviours. We finally note that the most adopted measure was also considered to be the least efficient by the participants in our study. This disconnect between perceived efficacy and adoption holds for few of the measures we considered, and was also reported in La Réunion where dengue is endemic [31]. This could stem from the varying efforts required to adopt each measure, and the fact that the adoption of each measure may depend on participants’ habitat and lifestyle habits.

In line with expectations from the Health Belief Model [29], the adoption of preventive measures in our sample was the result of a complex interplay between knowledge of dengue and attitudes, beliefs, socio-economic and contextual factors. In particular, raised concern levels driven in part by perceived susceptibility and severity, perceived efficacy of measures (akin to perceived benefits), as well as several individual characteristics (being female, not having an occupation, having comorbidities, being vaccinated and living in a rural area) increased the likelihood of adopting a larger number of preventive measures in our study. Moreover, the detection of local cases and having sought information appeared as cues to action which increased the likelihood of measures adoption. These results confirm previous findings highlighting the role of sex, knowledge, concern levels, perceived efficacy and education status in shaping the adoption of preventive measures against dengue and other mosquito-borne diseases [38–41]. In addition, our study also revealed the role of the participants’ information search behaviour, occupation and urbanicity in the adoption of preventive measures.

Individuals who had searched for information actively (most commonly from scientific websites and national newspapers) were also adopting a higher number of measures. The sample size did not allow for the investigation of the role of the specific information sources consulted in shaping measure adoption, an important aspect which should be the focus of future work. Being employed was associated negatively with the adoption of preventive measures, which may stem from differences in time use and opportunities for measure adoptions. Future research in this direction would be needed to disentangle differences in exposure and opportunities between these populations, in order to design targeted campaigns aimed at increasing measure adoption in the most vulnerable subgroups. Urbanicity was negatively associated with the adoption of preventive measures. This is a concerning finding, as *A. albopictus* mosquitoes adapted to thrive in urban environments where they face fewer predators, have access to a high density of human hosts, and many man-made structures where they can breed [66,67]. Future campaigns to encourage the uptake of preventive measures should therefore aim to raise knowledge, efficacy perceptions, inform on dengue severity and susceptibility, and should focus in particular on informing urban residents on the actions they can take to protect themselves against dengue. In contrast, information campaigns in rural areas — associated with higher adoption of preventive measures — would need to focus on raising awareness of dengue, improving knowledge of its characteristics, and promoting vaccination willingness, should a vaccine become available. Still, care must be taken to tailor these interventions to the local context, and current adoption of preventive measures. Indeed, our analysis revealed that the association between urbanicity and measure adoption was country dependent, with rural residence instead being negatively associated with the adoption of preventive measures in Italy. Intervention campaigns should carefully account for these differences, and be tailored to the local context in order to be equitable. This may be achieved by working directly with local organisations and community groups to identify the specific needs of the targeted population and bridge gaps in measure adoption.

Furthermore, the drivers of adoption of preventive measures identified by our study are partially in line with known drivers of preventive behaviours against COVID-19 [68], such as masking, self isolating when symptomatic, social distancing or increased hygiene. The effect of sex especially has been extensively investigated, with women repeatedly found more likely to engage in preventive behaviours during the COVID-19 pandemic [68–70]. Similarly, perceived efficacy of measures, and unemployment are both predictors of adoption of preventive behaviour against dengue in our study, and during the COVID-19 pandemic [69,71,72]. However, older age and higher education levels, which were often associated with the adoption of measures during the COVID-19 pandemic [68,70,72], were not significant factors in the context of dengue in our study. On the other hand, the higher likelihood of adopting preventive behaviours against COVID-19 in urban settings [73] is reversed in the context of dengue. This reversal could stem from the fundamental differences in preventive measures between the two diseases: common preventive measures against COVID-19 such as wearing a mask in public transport, working from home or avoiding restaurants may be more applicable in urban settings while preventive measures against dengue such as avoiding swampy areas, removing stagnant water or using mosquito nets may be more applicable instead in rural settings. This may also explain the fact that rural residence was associated with higher adoption of preventive measures, despite being negatively associated with knowledge, in line with existing evidence of higher health literacy of urban populations [74].

Our survey suggests that concern levels related to dengue fever remain moderate in France and Italy, despite the increasing number of cases reported [21]. Our study further sheds light on the main causes of concerns for individuals sampled. Fear of developing severe symptoms was the leading cause of worries in participants, followed by fears for close ones and relatives and fear of being infected. Participants were less worried about economic consequences due to contagion or a dengue epidemic. In line with findings from several empirical studies, this shows that dengue and more generally mosquito-borne diseases are not perceived as a significant threat in European populations [37,38,40,42], including by health professionals as evidenced by the low risk projections of specialised French medical doctors [43]. This is at odds with the anticipated increased risk of local outbreaks [25] due to the expansion of the vector on the European continent [18,19], and increase in climatic suitability for local spread [20].

Our study investigated attitudes towards vaccination against dengue in a non-endemic context. In line with the restricted eligibility for vaccination in Italy and mainland France, we observed a very low vaccination coverage in our sample, with only 11 vaccinated participants in total. Our survey evidenced a high vaccination willingness in the hypothetical scenario of a vaccine being offered, especially in light of the moderate concerns expressed about the consequences of dengue outbreaks. The willingness in the general French and Italian populations may be different from our estimates coming from participatory cohorts, who typically demonstrate higher vaccination coverage than the general population [51]. Still, our results suggest that, in the future, a potential vaccine may be well received if offered, especially if accompanied by effective communications highlighting the risk of dengue infection and severe outcomes for the population.

Willingness to be vaccinated was however higher in Italy than France, as observed previously before the COVID-19 vaccine roll-out [75]. We also identified a significant sex divide, with women less likely to accept vaccination, in line with numerous evidence on the impact of sex on vaccination willingness to prevent respiratory viruses [76,77]. Interestingly, we found that younger individuals were more likely to accept a vaccine, in contrast with findings related to respiratory viruses with a strong age dependent severity profile [76]. While dengue is also more severe in the elderly [78], this does not translate into higher vaccination willingness in this group. Higher educational attainment and employment were also associated with higher vaccination willingness. The local environment (urbanicity, and the detection of locally transmitted cases previously) also positively influenced the hypothetical uptake together with concern levels. Our findings on the negative association of the knowledge of a case on vaccination willingness is unexpected. This effect deserves further investigation. Instead, reasons to refuse vaccination most often cited were related to risk perception, either through perceived susceptibility (*“I don’t think I will get dengue”*) or perceived severity (*“I don’t belong to any risk group”*). Our analysis suggested the importance of the broader social context in shaping vaccination refusal attitudes through the country of residence. In both cases, individuals who reported higher concerns for dengue consequences in the survey were less likely to cite either reason. Little overlap in determinants were found, suggesting the unique interplay of individual characteristics shaping each refusal reason.

Novel insights, such as those presented here, can be obtained rapidly and at low cost by polling volunteers already engaged in participatory surveillance cohorts. The platforms in the InfluenzaNet network were initially designed for influenza-like illnesses surveillance [46], and rapidly evolved during the pandemic to capture COVID-19 illnesses. This was instrumental to evaluate knowledge, attitudes and practices at the beginning of the first wave of COVID-19 in March 2020 in several European countries [50], and later on, to reassess attitudes towards testing and boosting vaccination as the context evolved [47]. With the present study, we illustrate how participatory surveillance cohorts can also be leveraged to investigate emerging threats, unrelated to respiratory viruses. Participatory surveillance platforms have moreover been integrated in public health surveillance of respiratory viruses, for example in the UK [79] and in the Netherlands [80]. In the future, participatory surveillance platforms could also provide integrated monitoring tools and early warning systems for dengue and other mosquito-borne diseases, but authorities should account for representativeness limitations when using such data for policy decisions. Further research leveraging these platforms could moreover investigate the evolution of knowledge, attitudes and practices regarding dengue, as well as other vector borne diseases over longer timespans. This would provide necessary data to reveal the impact of concurrent outbreaks on knowledge, attitude and behaviours, as well as the role of seasonality in shaping concern levels and preventive behaviours. These findings could also be analysed through additional indicators related for example to information exposure [81]. Finally, future efforts remain necessary to understand the drivers of exposure to dengue, related to individual habitats, lifestyle and behaviours, and their link to knowledge, attitudes, and the adoption of preventive measures.

Our study has several limitations. First, participatory surveillance exhibits an intrinsic selection bias in the samples. As participation is voluntary, the population surveyed may be more likely to engage with health recommendations, protective behaviours, and to consider vaccination positively. In the context of this study, this residual bias may lead to the observation of slightly higher levels of knowledge, concern levels and adoption of preventive measures compared to the general population. This bias could only be corrected by using different costly collection methods such as representative panels or door to door collection. Moreover, participants to InfluenzaNet cohorts participate online, limiting the representation of individuals with limited access to the internet in the sample. Moreover, the sample did not include respondents below 18 years old for ethical reasons, and participants aged 18–19 years old were excluded to align with available census data for post stratification. As a result, these younger age groups were not considered in our study, but may still be at risk of exposure to dengue. All age classes above 20 years old, sex, and education levels were represented in our sample. Despite post-stratifying by age, sex, and educational attainment, the representativeness of health related attitudes and behaviour may be limited, and care must be taken when generalising our findings to the general population, especially in Italy for which our sample size is smaller. For example, influenza vaccination coverage in InfluenzaNet cohorts is slightly higher than in the general population [82], [83] while incidence trends of influenza-like illnesses obtained from InfluenzaNet cohorts are consistent with ECDC trends in terms of timing and magnitude [82]. Individual risk factors for influenza-like illnesses obtained from InfluenzaNet cohorts have also been demonstrated to be consistent with the literature [82]. As a result, vaccination willingness and adoption of preventive behaviours may be lower in the general population compared to our sample; but we expect determinants and associations observed in our analysis to persist. Second, conducting the analysis on pooled French and Italian samples could have obscured some differences between the two countries. The homogeneity of the two samples was assessed by considering possible interactions between each determinant and the country variable. This analysis already demonstrates some country differences, while the majority of the effects were shown to be independent of the country. A more detailed comparison of knowledge, attitudes and behaviours related to dengue in different countries is left for future work on larger sample sizes. Third, answers to some of the questions in our survey may have been affected by recall bias; in particular when it comes to reported vaccination status, presence of dengue cases among contacts, and symptoms history. Vaccination against dengue is generally not offered in mainland France and Italy, with the exception of travellers, on a case by case basis. As a result, respondents who had received the vaccine would have done so in very specific circumstances, and are therefore likely to remember. Similarly, the symptoms proposed to the respondents were specific to dengue, and rarely occur, such that their manifestation would be highly memorable. Moreover, symptom history and the presence of dengue diagnoses among acquaintances were reported for the past 30 days, a period during which we expect the majority of participants to recall such rare and notable events. Fourth, all preventive measures were considered for all participants, regardless of their relevance in each participant’s individual context. For example, individuals in apartments may not have any opportunity to remove stagnant water or avoid swampy areas, while participants who rarely travel may not need to avoid travelling to at-risk areas. In order to mitigate this, we considered the total number of adopted measures as an outcome, instead of each measure individually. Although the questions specifically addressed measures to prevent dengue infection, we also cannot rule out that some participants reported actions motivated instead by a desire to reduce mosquito nuisance [41]. Fifth, the symptom list provided to participants in order to capture recent dengue-like symptom history was designed for epidemiological specificity, and did not include flu-like symptoms of dengue shared with common infections endemic to Europe, in line with our aim to capture the impact of experiencing dengue-specific symptoms on knowledge, attitudes and practices. The variable *Symptoms* was defined by requiring the presence of at least two of five of the proposed symptoms, in order to avoid misclassification from isolated symptoms. For sensitivity, we considered alternative definitions based on the presence of at least one symptom and at least three symptoms. The significance of the association of the symptom history with the outcomes considered were not robust to these changes, but the remainder of the model results were overall robust to this change. Sixth, autochthonous dengue outbreaks occurred in France and Italy during the data collection, and it is possible that these have impacted the levels of knowledge, concerns and adoption of preventive measures reported by participants replying later to the questionnaire. However, more than 85% of the responses considered in the Italian sample were gathered prior to the detection of the first autochthonous outbreaks in Italy in 2024. Moreover, the French sample was collected over a timespan of one week between July 29th and August 6th 2024. It is therefore highly unlikely that the results of this study were significantly affected by the development of autochthonous outbreaks concurrent to the data collection. Similarly, seasonal variations during the data collection are unlikely to affect the results as the majority of the answers were collected over a timespan shorter than typical seasonal variations. But the timing of the data collection may have itself led to higher rates of preventive behaviours and higher concern levels than would have been observed at other timepoints during the year. Future longitudinal data collection would be needed to elucidate this aspect. Finally, our questionnaire did not address participants’ perceptions towards mosquitoes (discomfort felt with bites, perceived biting frequency, perceived *Aedes* mosquito presence), which were shown in 2012 to be associated with the adoption of preventive behaviours [38,40]. This factor, among other unaccounted for characteristics, such as type of habitat, household composition, and cultural background from dengue-endemic regions, may act as confounders in our analysis. Future data collection using longer and more detailed surveys may address these aspects.

## 5. Conclusions

Participatory surveillance cohorts provide a unique opportunity to probe engaged participants on their knowledge, attitudes, behaviours and practices related to dengue and other mosquito borne diseases. We surveyed 2,904 participants of the French and Italian cohorts of the InfluenzaNet network in 2024. Our cross-sectional analysis demonstrates a high awareness of the existence of dengue, but a heterogeneous knowledge of its different characteristics, suggesting an urgent need to raise awareness and educate populations on lesser known aspects. Statistical determinants of awareness, knowledge, adoption of preventive measures and attitudes towards vaccination (willingness, and reasons for refusal) revealed a rich interplay between knowledge levels, concerns, attitudes, behaviours, as well as socio-demographic and contextual characteristics. Notably, active information search and higher concern levels were found to correlate with knowledge, adoption of measures, and willingness to vaccinate. Knowledge in turn was associated with adoption of preventive measures, alongside perceived efficacy, local epidemiological context, occupation of participants, sex, vaccination and symptom history. Targeted, accessible, and locally relevant communication strategies accounting for these factors are essential to enhance preparedness and outbreak prevention in regions where dengue poses an increasing threat.

## Supporting information

S1 Appendix Additional elements pertaining to the study, including survey text, methodological supplementary details and additional results. (PDF)
