# Supplementary material for: Assessing dengue knowledge, attitudes and preventive practices in France and Italy: A cross-sectional study using participatory surveillance cohorts
Source: PLOS Glob Public Health. 2026 Sep 23;6(9):e0007162. doi: 10.1371/journal.pgph.0007162 (PMC13600492; doi:10.1371/journal.pgph.0007162)
Supplement: Supplementary file 1 [file pgph.0007162.s001.pdf]

Supplementary Materials to: **Assessing dengue knowledge, attitudes and preventive practices in France and Italy: a cross-sectional study using participatory surveillance cohorts.**

Lucille Calmon[1,2,*], Alessandro De Gaetano[2,3,*], Mattia Mazzoli[2], Nicolò Gozzi[2], Marion Debin[1], Clément Turbelin[1], Romain Marmorat[1], Nicola Perra[4,5], Alain Barrat[3], Vittoria Colizza[1,†] and Daniela Paolotti[2,†,‡]

[1] Sorbonne Université, INSERM, Pierre Louis Institute of Epidemiology and Public Health, Paris, France

[2] ISI Foundation, Turin, Italy

[3] Aix Marseille Univ, Université de Toulon, CNRS, CPT, Marseille, France

[4] School of Mathematical Sciences, Queen Mary University of London, United Kingdom

[5] The Alan Turing Institute, London, United Kingdom

[*] equal contributions

[†] equal contributions

[‡] corresponding author

Table of contents

Table of contents **2**

- **Survey text** **2**
- **Post processing of the GrippeNet sample** **5**
- **Respondents vs non-respondents** **5**
- **Bias adjustment** **7**
- **Logistic regression models** **7**
- **Ordinal regression models** **7**
- **Sensitivity analysis on the variable selection method and model specification** **8**
- **Proportionality of odds assumption** **13**
- **Variance Inflation Factor** **15**
- **Interaction terms** **17**
- **Sensitivity on the definition of symptom history** **21**
- **Additional tables** **25**

Survey text

dengue.qd2 : Are you aware about the existence of an infectious disease called Dengue in your country or around the world?
1- Yes
2- No

dengue.qd1 : Did you experience one or more of these symptoms in the past month? [multiple choice]
24 - Eye pain
25 - Blood in the feces
26 - Blood in the vomit
27 – Bloody gums
28 - Bone pain
0 - No symptom

> **dengue.qd1b** : (if dengue.qd1!=0) Did you travel abroad, recently?
> 1- Yes
> 0- No
>
> > **dengue.qd1c** : (if dengue.qd1b = yes) To which country did you travel?
> > 0 - text box
> >
> > **dengue.qd1d** : (if dengue.qd1b = yes) Were you abroad when the symptoms started?
> > 1- Yes
> > 0- No
>
> If dengue.qd2 = No ⇒ end of survey.

dengue.qd2b : Have you ever looked for information on the spread of Dengue in your country?
1- Yes
0- No

> **dengue.qd2c** : (if dengue.qd2b=yes) Where did you look for information on the Dengue situation in your country?
> 0 - Scientific information and dissemination web sites
> 1 - Foreign newspapers
> 2 - National newspapers
> 3 - Local newspapers
> 4 - Blogs (Reddit, etc.)
> 5 - Social media (TikTok, Instagram, Facebook, X (Twitter), etc)
> 6 - Your doctor
> 7 - Other (Please, specify where else you looked for information)

dengue.qd3 : How would you rate your level of information about Dengue?
1 - Very poor or none at all
2 - Poor
3 - Average
4 - Accurate
5 - Very accurate

dengue.qd4 : Are you aware of the following characteristics of the Dengue disease?
0 - Dengue is transmitted through bites of infected Aedes Albopictus (tiger mosquito) or Aedes Aegypti mosquitoes (yes/no)
1 - Mild cases of Dengue have symptoms similar to Covid-19 or the Flu (yes/no)
2 - Dengue can be asymptomatic (yes/no)
3 - Symptoms last on average between 2 and 7 days (yes/no)
4 - Severe cases of Dengue can lead to hospitalization and death (yes/no)
5 - There is no specific treatment or medicine against Dengue (yes/no)
6 - If you have already had Dengue in the past, you are more at risk of having severe symptoms (yes/no)
7 - Pregnant women and children are more at risk of having severe symptoms (yes/no)

dengue.qd5 : To what extent are you preoccupied by the following consequences of Dengue ?
QD5_0 - Fear of being infected (from 0 to 4)
QD5_1 - Fear of having severe symptoms if I get sick (from 0 to 4)
QD5_2 - Fear for my friends/family (from 0 to 4)
QD5_3 - Fear for my work/economic consequences in case of contagion (from 0 to 4)
QD5_4 - Fear of my work/economic consequences in the event of a pandemic (from 0 to 4)

def **worry**(de 0 à 4):{
0 - Not at all worried
1 - A little worried
2 - Quite worried

3 - Very worried
4 - Extremely worried
}

dengue.qd5b : (if QD5 != 0) Why are you worried ? [multiple choice]
0 - I live/work in an area with many mosquitos
1 - My friends/family live or work in an area with a lot of mosquitoes
2 - I often travel to areas where Dengue is endemic
3 - My friends/family often travel to areas where Dengue is endemic
4 - I have health problems (e.g. immunosuppression)
5 - My friends/family have health problems (e.g. immunosuppression)
6 - Other (Please, specify why you are worried)

dengue.qd6 : Did you get the vaccine against Dengue?
1- Yes
0- No

dengue.qd6a : (if dengue.qd6 = No) Would you get vaccinated for Dengue if you were offered a vaccine?
1- Yes
0- No

dengue.qd6b : (if dengue.qd6a = No) Why wouldn't you get vaccinated against Dengue ? [multiple choice]
0 - I don't belong to any risk group
1 - It is better to build your immune defences naturally
2 - I have doubts about the effectiveness of the vaccine
3 - I don't think I'll get dengue
4 - I am concerned that the vaccine is unsafe or may cause illness or side effects
5 - I don't like receiving vaccinations
6 – The vaccine is currently too expensive
7 - My doctor advised me against getting vaccinated
8 - None of the above (Please, specify the reason)

dengue.qd7 : How effective do you think the following measures are in reducing the risk of getting Dengue?
QD7_0 - Wear long-sleeved clothing covering your arms and legs (from 0 to 4)
QD7_1 - Use mosquito repellent regularly (from 0 to 4)
QD7_2 - Avoid swampy areas or areas with a high density of mosquitoes (from 0 to 4)
QD7_3 - Avoid travelling to at risk areas (from 0 to 4)
QD7_4 - Install mosquito nets on the windows of your home (from 0 to 4)
QD7_5 - Remove stagnant water from the balcony, windows and garden of your home (from 0 to 4)

def **score**(de 0 à 4):{
0 - Not effective at all
1 - Not very effective

2 - Quite effective
3 - Very effective
4 - Extremely effective
}

dengue.qd8 : Have you been diagnosed with Dengue in the last month?
1 – Yes
0 - No

dengue.qd8b : Have you heard of anyone diagnosed with Dengue in the last month?
1 – Yes
0 - No

dengue.qd8c : (ifi dengue.qd8b=yes) This person is:
0 – In my household
1 - A friend/acquaintance with whom I have frequent contact
2 - A person I don't know directly or rarely see

dengue.qd9 : Have you adopted the following behaviours to reduce the chances of contracting Dengue? [multiple choice]
0 - I wore long-sleeved clothing covering my arms and legs
1 - I regularly used mosquito repellent
2 - I avoided swampy areas or areas with a high density of mosquitoes
3 - I avoided travelling/cancelled a trip to areas at risk
4 - I have installed mosquito nets in all windows
5 - I removed all stagnant water from the balcony, garden and windows
6 – None

Post processing of the GrippeNet sample

The questionnaire sent to the GrippeNet cohort in France remained open, and participants were technically able to submit more than one response. We applied the following cleaning procedure in order to retain one answer for each participant, avoid bias due to previous exposure to the questions, and allow for minor correction.

In case of duplicate replies by a single participant within 24h, we included the latest answers to factual questions (qd1, qd1b, qd1c, qd1d, qd6, qd8b), while keeping initial answers for the rest of the questionnaire. Otherwise, we included the initial reply.

We included replies where answers to qd9 were consistent, i.e. we eliminated participants who had answered “yes” to “None of the measures listed” while listing other measures.

Respondents vs non-respondents

We compare in **Table A** the characteristics of respondents to the questionnaire to non-respondents users of the platforms. Non-respondents users were defined for each

platform as registered users who were active on the platform in the year prior to the study, but who did not participate in this specific study.

**Variable**	**Level**	**Italy**			**France**		
		**Resp.**	**Resp. after cleaning**	**Active non-resp.**	**Resp.**	**Resp. after cleaning**	**Active non-resp.**
**Sex**	Male	53.7%	54.5%	53.1%	41.8%	42.4%	40.0%
	Female	46.3%	45.5%	47.0%	58.2%	57.6%	59.9%
	missing	0%	NA	0%	0%	NA	0.1%
**Age group**	18-19	0.9%	NA	1.7%	0.1%	NA	1.3%
	20-44	21.6%	19.3%	27.7%	9.4%	9.2%	15.4%
	45-64	50.2%	51.7%	45.2%	36.4%	35.8%	37.6%
	65+	22.5%	29.0%	16.3%	53.4%	55.0%	40.9%
	missing	4.8%	NA	9.0%	0.8%	NA	4.9%
**Education level**	Until high school	47.9%	48.5%	44.9%	27.6%	28.0%	27.9%
	Post high school	51.1%	51.5%	55.1%	70.8%	72.0%	67.1%
	missing	0.9%	NA	0%	1.5%	NA	5.1%
**Occupation**	Yes	61.7%	61.6%	63.8%	39.4%	38.5%	45.9%
	No	38.3%	38.4%	36.2%	60.6%	61.5%	54.1%
	missing	0%	NA	0%	0%	NA	0%

Table A: Characteristics of the respondents to the study compared to the non-respondent active users of the platforms. *We report the percentage of users by sex, age group, education level and occupation. For each country, the column "Resp." refers to all respondents to the study (before application of the inclusion criteria). The column "Resp. after cleaning" refers to participants meeting inclusion criteria and considered in the analysis. The column "Active non-resp." finally refers to the users active in the year prior to the study who did not respond to the survey.*

Bias adjustment

We considered three age groups: (20-44), (45-59), and (60+), two genders: male and female, as well as two education groups: up to high-school and higher (post secondary) education.

When calculating the frequency of X (e.g. the frequency of individuals that know about the existence of dengue in their country – **qd2=1**), we compute the adjusted frequency f(X) as:

$f(X = 1) = \frac{\sum_{k|X=1} w_k}{\sum_k w_k}$ where k indexes the participants, w_k is the weight of participant k of age group a, gender g, and education level e given by $w_k = \frac{rp_{age}}{sp_{age}}$ where rp_{age} is the fraction of individuals in age group a, of gender g, and of education level e in the reference population, and sp_{age} is the fraction observed in the survey sample.

Logistic regression models

The probability of a binary outcome Y (i.e. being willing to vaccinate, qd6a = 1) is modelled as

$logit(p(Y = 1)) = z = b_0 + x_1 b_1 + x_2 b_2 + ... + x_p b_p$

The variables $\{x_1, x_2, ...\}_k$ are the features (determinants) of participant k. The parameters $b_0, b_1, b_2 ...$ are evaluated via maximum likelihood estimation.

The unadjusted likelihood is given by $L_i = \prod_{k\ s.t.\ qd2_k=1} \frac{1}{1+e^{-z_k}} \prod_{k\ s.t.\ qd2_k=0} (1 - \frac{1}{1+e^{-z_k}})$ where k indexes the participants. In order to adjust for age, education and gender biases in the sampled population, the likelihood is weighted as follows.

Labelling $p_k = \frac{1}{1+e^{-z_k}}$ the modelled probability of knowledge of dengue for the features of participant k, we maximise the adjusted likelihood $\widehat{L_i} = \prod_k p_k^{w_k X_k} * (1 - p_k)^{w_k(1-X_k)}$ where w_k is the weight of the participant k, calculated as described in the previous section.

The exponential of the coefficients $\{b\}$ provide the odd ratios

$e^b = [P(Y = 1|x = 1)/P(Y = 0|x = 1)]/[P(Y = 1|x = 0)/P(Y = 0|x = 0)]$

Ordinal regression models

We accounted for the fact that the different knowledge levels, and different numbers of adopted preventive measures are ordinal. They cannot be measured as continuous, as the underlying “distance” between each score may not be constant (see Ref(1)).

We used a “proportional odds logistic model”(2), which models the cumulative probability of the response with a logistic regression model. In R, the model is defined as(3)

$$logit(p(Y \leq j)) \ = \theta_{0j} - \ x_1 b_1 - \ x_2 b_2 - ... \ - x_p b_p$$

where Y is the ordinal variable with levels specified by j, θ_{0j} are baseline odds for each level j. These can be interpreted in terms of thresholds between the levels, see Refs(2,4) for this interpretation. As before, $\{x\}$ are the features of the participants. The baseline odds θ_{0j} are also fitted via MLE. The coefficients b_1, b_2... are the same for all scores j, and are fitted via MLE.

The exponential of the coefficients $\{b\}$ provide the odd ratios(3)

$$e^b = [P(Y > j|x = 1)/P(Y \leq j|x = 1)]/[P(Y > j|x = 0)/P(Y \leq j|x = 0)]$$

which can be interpreted as the odds of being in a category higher than j when having attribute x = 1 instead of the reference level 0. This odd ratio is the same across all ordinal categories of the outcomes. This is the proportionality of odds assumption. In other words this means that if an attribute raises by e^b the likelihood of scoring 2 or higher on a Likert scale, it also raises by the same coefficient the likelihood of scoring 3 or higher.

Sensitivity analysis on the variable selection method and model specification

To assess the robustness of our variable selection procedure, we compared for each outcome the selected model via backward stepwise selection to the model with highest adjusted McFadden’s pseudo R^2, obtained with an exhaustive search among all the possible combinations of the explanatory variables.

The results of this analysis confirmed the robustness of our selection method. For the outcome *Awareness of dengue*, the alternative method selected the identical set of variables (pseudo R^2 = 0.034). For most other outcomes, the differences were minor and the model fit was nearly identical. For instance:

- For *Knowledge of characteristics*, the best R^2 model (0.036) only differed by the inclusion of the country variable compared to our main model (0.035).
- For *Adoption of measures*, it added country and age group for a marginal improvement in pseudo R^2 (0.078 vs. 0.077).
- For *Willingness to be vaccinated*, the only difference was the inclusion of comorbidities, with the pseudo R^2 changing only in the fourth decimal place (0.1085 vs. 0.1083).
- For *I don’t belong to any risk group*, the best R^2 model (0.068) merely added the symptoms variable compared to our main model (0.067).

The only outcome with a notable difference was *I don't think I'll get Dengue*. While the best R^2 model included several additional variables (*searched information, knowledge score, known case, age group, education level*) and had a higher pseudo R^2 (0.094 vs. 0.075), most of these added variables were not statistically significant.

Overall, this sensitivity analysis demonstrates that our two-step variable selection method yields robust models that are highly consistent with those from an exhaustive search, strengthening the credibility of our results.

Furthermore, we tested the sensitivity of our results to alternative model specifications. The first alternative approach involved fitting logistic or ordinal regression models (depending on the specific outcome) using the full set of covariates, without applying any variable selection. The resulting odds ratios and their corresponding confidence intervals for each outcome are presented in **Figure A** and **Figure B**.

Overall, the results are robust and the variables that were discarded in our main models in the variable selection process are not statistically significant, with a few exceptions:

- For *Awareness of Dengue*, the variable *sex* is non-significant in the full model, while it is in our main model (with variable selection).
- For *Knowledge of Characteristics*, the variable *Country* is significant in the full model, but is not using our procedure with backward stepwise selection.
- For *I don't belong to any risk group*, the country variable is not significant while in our main model it is (although the significance is close to the threshold).
- For *I don't think I'll get Dengue*, the variables *education level* and the lowest knowledge score (poor) are significant while they weren't in our main model.

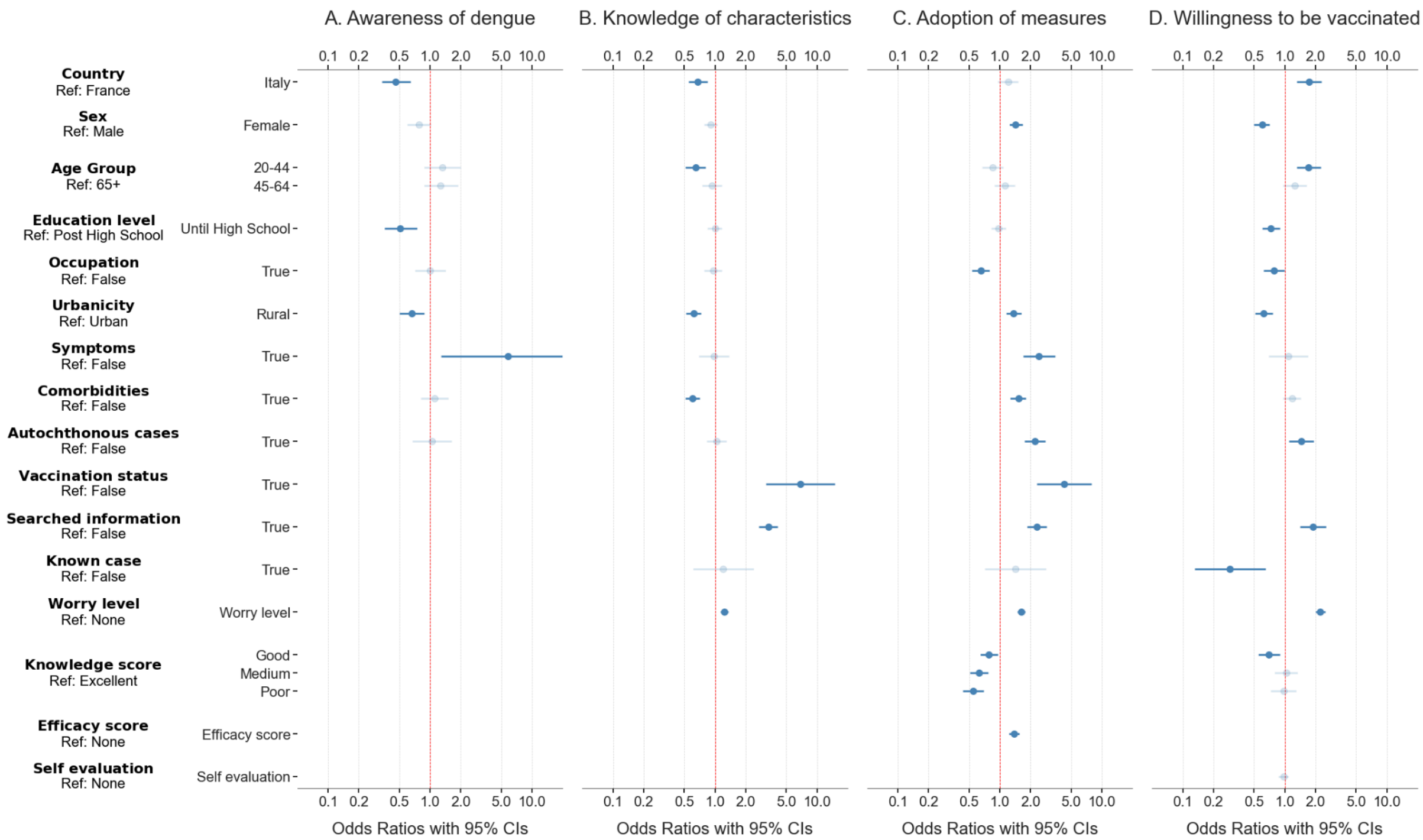

Figure A. Determinants of knowledge of dengue, its characteristics, adoption of preventive measures, and vaccination willingness, full models. A) *Determinants of the awareness of dengue identified with a logistic regression model in the form of odds ratios with 95% confidence intervals.* ***B)*** *Determinants of the knowledge of dengue characteristics, obtained with an ordinal regression model applied to the answers of the participants who have heard of dengue.* ***C)*** *Determinants associated with adoption of preventive numbers (obtained from an ordinal regression model, among participants who have heard of dengue).* ***D)*** *Determinants of willingness to be*

vaccinated (binary logistic model) among the participants who are unvaccinated. Models are multivariate, with all explanatory variables included (no variable selection). Variables without an OR are not entering the analysis for the model they have no OR, and are shown for all models for visualisation purposes. Faded points represent non significant levels of included variables. All analyses are conducted on the pooled dataset including French and Italian samples.

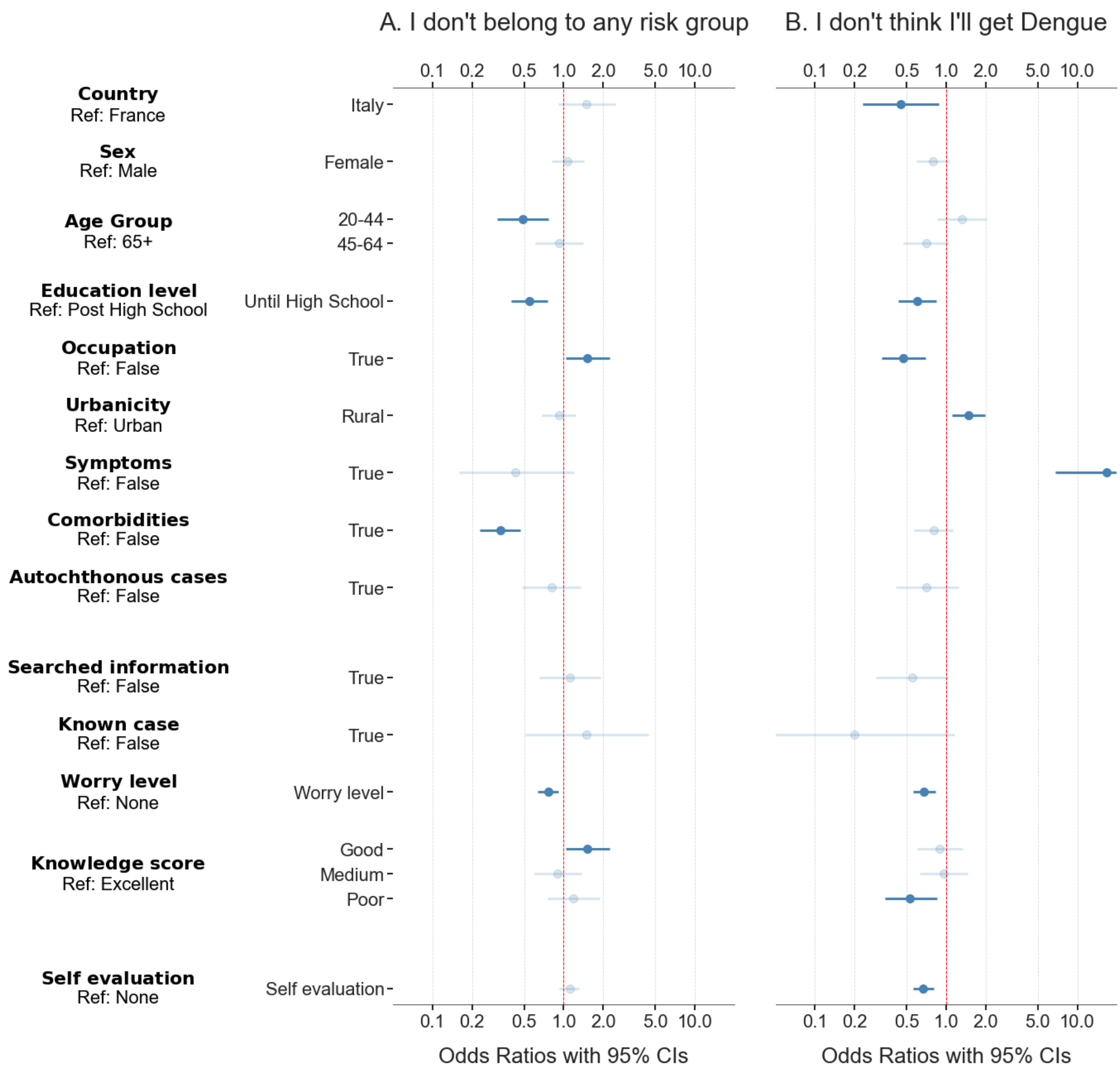

***Figure B. Determinants of the main reasons for vaccine refusal, full models. A)** Determinants of citing "I don't belong to any risk group" in the form of odds ratios with 95% confidence intervals. **B)** Determinants of citing "I don't think I'll get dengue" in the form of odds ratios with 95% confidence intervals. Odds ratios were obtained from a multivariate, bias adjusted logistic regression model applied to the participants who are unwilling to be vaccinated. Models are multivariate, with all explanatory variables included (no variable selection). Faded points represent non significant levels of included variables. All analyses are conducted on the pooled dataset including French and Italian samples.*

Second, for the binary outcomes, we introduced an L2-norm regularisation penalty (Ridge regression) to shrink potentially overestimated effects associated with wide confidence intervals. **Tables B–E** compare these regularised results against our main models. These tables detail the coefficient shrinkage and verify that the direction of the effects remains consistent with our primary findings. In all cases, the direction of the effects is confirmed and remains robust to the choice of the regularisation parameter, C.

Categorical Variable	Variable	Reference	OR ridge model	OR main model	P-value main model	Agreement of direction	Shrinkage
Country	Italy	France	0.55	0.46	<0.001	True	0.84
Sex	Female	Male	0.83	0.76	0.041	True	0.71
Education level	Until High School	Post High School	0.63	0.49	<0.001	True	0.72
Urbanicity	Rural	Urban	0.74	0.65	0.003	True	0.76
Symptoms	True	False	2.59	6.29	0.016	True	0.30

Table B. Ridge regression sensitivity analysis for Awareness of dengue. *Comparison between odds ratios (ORs) from the main weighted logistic regression model (backward stepwise selection) and from a Ridge-penalised logistic regression (L2 penalty, C = 0.01) fitted on the same predictors with survey weights. Predictors were standardised prior to Ridge estimation; coefficients were back-transformed to the original scale for comparability. The Shrinkage column reports the ratio of the Ridge OR minus 1 to the main OR minus 1, indicating how much the regularisation pulls each association toward the null; values close to 1 indicate minimal shrinkage. All associations with direction agreement between the two models support the robustness of the findings reported in the main analysis.*

Categorical Variable	Variable	Reference	OR ridge model	OR main model	P-value main model	Agreement of direction	Shrinkage
Country	Italy	France	1.57	1.69	<0.001	True	0.82
Sex	Female	Male	0.66	0.59	<0.001	True	0.84
Age Group	20-44	65+	1.46	1.70	<0.001	True	0.66
Age Group	45-64	65+	1.10	1.26	0.086	True	0.37
Education level	Until High School	Post High School	0.81	0.74	0.003	True	0.74
Occupation	True	False	0.88	0.76	0.020	True	0.50
Urbanicity	Rural	Urban	0.67	0.62	<0.001	True	0.85
Autochthonous cases	True	False	1.42	1.46	0.009	True	0.90
Searched information	True	False	1.74	1.90	<0.001	True	0.82
Known case	True	False	0.41	0.29	0.002	True	0.83
Worry level	Worry level	None	1.91	2.23	<0.001	True	0.74
Knowledge score	Poor	Excellent	1.01	1.03	0.848	True	0.44
Knowledge score	Medium	Excellent	1.05	1.05	0.682	True	1.00
Knowledge score	Good	Excellent	0.77	0.71	0.006	True	0.79

Table C. Ridge regression sensitivity analysis for Willingness to be vaccinated. *Comparison between odds ratios (ORs) from the main weighted logistic regression model (backward stepwise selection) and from a Ridge-penalised logistic regression (L2 penalty, C = 0.01) fitted on the same predictors with survey weights. Predictors were standardised prior to Ridge estimation; coefficients were back-transformed to the original scale for comparability. The Shrinkage column reports the ratio of the Ridge OR minus 1 to the main OR minus 1,*

indicating how much the regularisation pulls each association toward the null; values close to 1 indicate minimal shrinkage. All associations with direction agreement between the two models support the robustness of the findings reported in the main analysis.

Categorical Variable	Variable	Reference	OR ridge model	OR main model	P-value main model	Agreement of direction	Shrinkage
Country	Italy	France	1.60	1.64	0.040	True	0.94
Age Group	20-44	65+	0.50	0.47	0.001	True	0.94
Age Group	45-64	65+	0.94	0.89	0.583	True	0.61
Education level	Until High School	Post High School	0.54	0.53	0.000	True	0.97
Occupation	True	False	1.48	1.54	0.021	True	0.89
Comorbidities	True	False	0.34	0.32	0.000	True	0.97
Worry level	Worry level	None	0.77	0.76	0.003	True	0.95
Knowledge score	Poor	Excellent	1.04	1.06	0.773	True	0.67
Knowledge score	Medium	Excellent	0.87	0.88	0.537	True	1.07
Knowledge score	Good	Excellent	1.45	1.50	0.033	True	0.90

Table D. Ridge regression sensitivity analysis for I don't belong to any risk group. *Comparison between odds ratios (ORs) from the main weighted logistic regression model (backward stepwise selection) and from a Ridge-penalised logistic regression (L2 penalty, C = 0.1) fitted on the same predictors with survey weights. Predictors were standardised prior to Ridge estimation; coefficients were back-transformed to the original scale for comparability. The Shrinkage column reports the ratio of the Ridge OR minus 1 to the main OR minus 1, indicating how much the regularisation pulls each association toward the null; values close to 1 indicate minimal shrinkage. All associations with direction agreement between the two models support the robustness of the findings reported in the main analysis.*

Categorical Variable	Variable	Reference	OR ridge model	OR main model	P-value main model	Agreement of direction	Shrinkage
Country	Italy	France	0.50	0.34	0.001	True	0.75
Occupation	True	False	0.72	0.61	0.000	True	0.73
Urbanicity	Rural	Urban	1.29	1.36	0.033	True	0.80
Symptoms	True	False	6.35	12.89	0.000	True	0.45
Worry level	Worry level	None	0.73	0.65	0.000	True	0.75
Self evaluation	Self evaluation	None	0.81	0.73	0.000	True	0.69

Table E. Ridge regression sensitivity analysis for I don't think I'll get dengue. *Comparison between odds ratios (ORs) from the main weighted logistic regression model (backward stepwise selection) and from a Ridge-penalised logistic regression (L2 penalty, C = 0.01) fitted on the same predictors with survey weights. Predictors were standardised prior to Ridge estimation; coefficients were back-transformed to the original scale for comparability. The Shrinkage column reports the ratio of the Ridge OR minus 1 to the main OR minus 1,*

indicating how much the regularisation pulls each association toward the null; values close to 1 indicate minimal shrinkage. All associations with direction agreement between the two models support the robustness of the findings reported in the main analysis.

Proportionality of odds assumption

We evaluated the proportionality of odds for the two outcomes that we modelled with an ordinal regression. For an outcome, this consists in stratifying the participants in two classes with different binary thresholds, conducting binary logistic regressions, and comparing the resulting fitted logits for the different thresholds chosen(5,6).

Number of dengue characteristics known

The knowledge score takes four categories: poor (level 1), medium (level 2), good (level 3) and excellent (level 4).

We binarised the data set, considering different thresholds to define three new outcome variables:

- "bks>= 2": Poor (0) – medium and above (1)
- "bks>= 3": Poor and medium (0) – good and excellent (1)
- "bks>= 4": Poor, medium and good (0) – excellent (1)

For each threshold, and for each discrete explanatory variable, we performed univariate binary logistic regressions, and compared the resulting logit fitted.

This resulting logit should not depend on the threshold chosen. The validity of the assumption was assessed by plotting the difference in logits for pairs of thresholds (**Figure C**). We find that the assumption of proportional odds holds for the majority of considered variables, but is less valid for *symptoms*, *searched information*, *known case* and *vaccination status*.

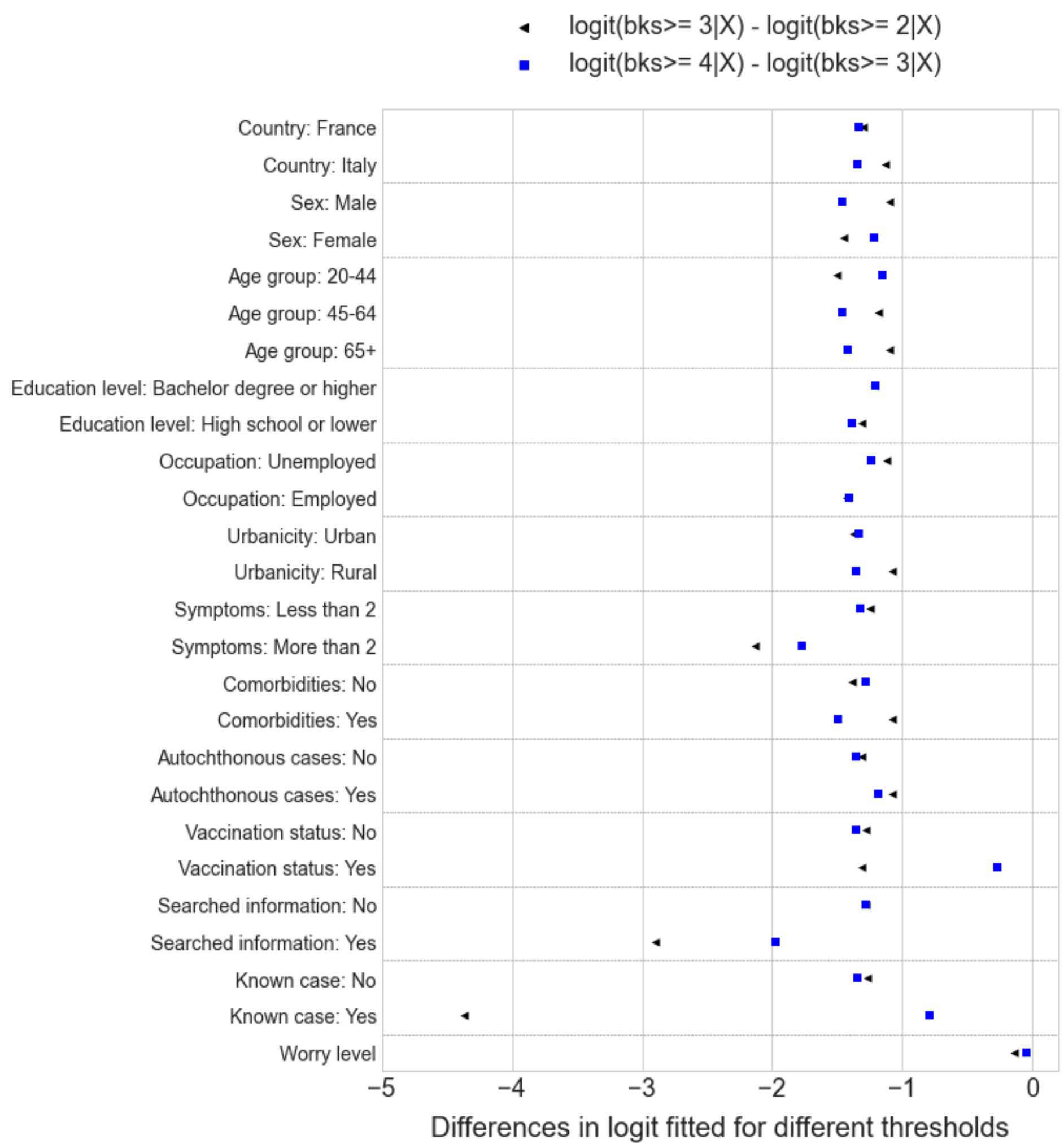

Figure C. Proportionality of odds of the knowledge score. *Differences in fitted logits are shown for each explanatory variable considered. In the legend, X refers to the level considered for each explanatory variable.*

Number of preventive measures taken

The outcome variable for the number of preventive measures taken takes four levels: poor (level 1), medium (level 2), good (level 3) and excellent (level 4).

As for the knowledge score, we binarised the data set, considering different thresholds to define three new outcome variables:

- "bks>= 2": Poor (0) – medium and above (1)
- "bks>= 3": Poor and medium (0) – good and excellent (1)
- "bks>= 4": Poor, medium and good (0) – excellent (1)

For each threshold, and for each discrete explanatory variable, we performed univariate binary logistic regressions, and compared the resulting logit fitted.

This resulting logit should not depend on the threshold chosen. The validity of the assumption was assessed by plotting the difference in logits for pairs of thresholds (**Figure D**). We find that the assumption of proportional odds holds for the majority of considered variables, but is less valid for the *symptoms*, *known case, urbanicity, knowledge score,* and *vaccination status* explanatory variables.

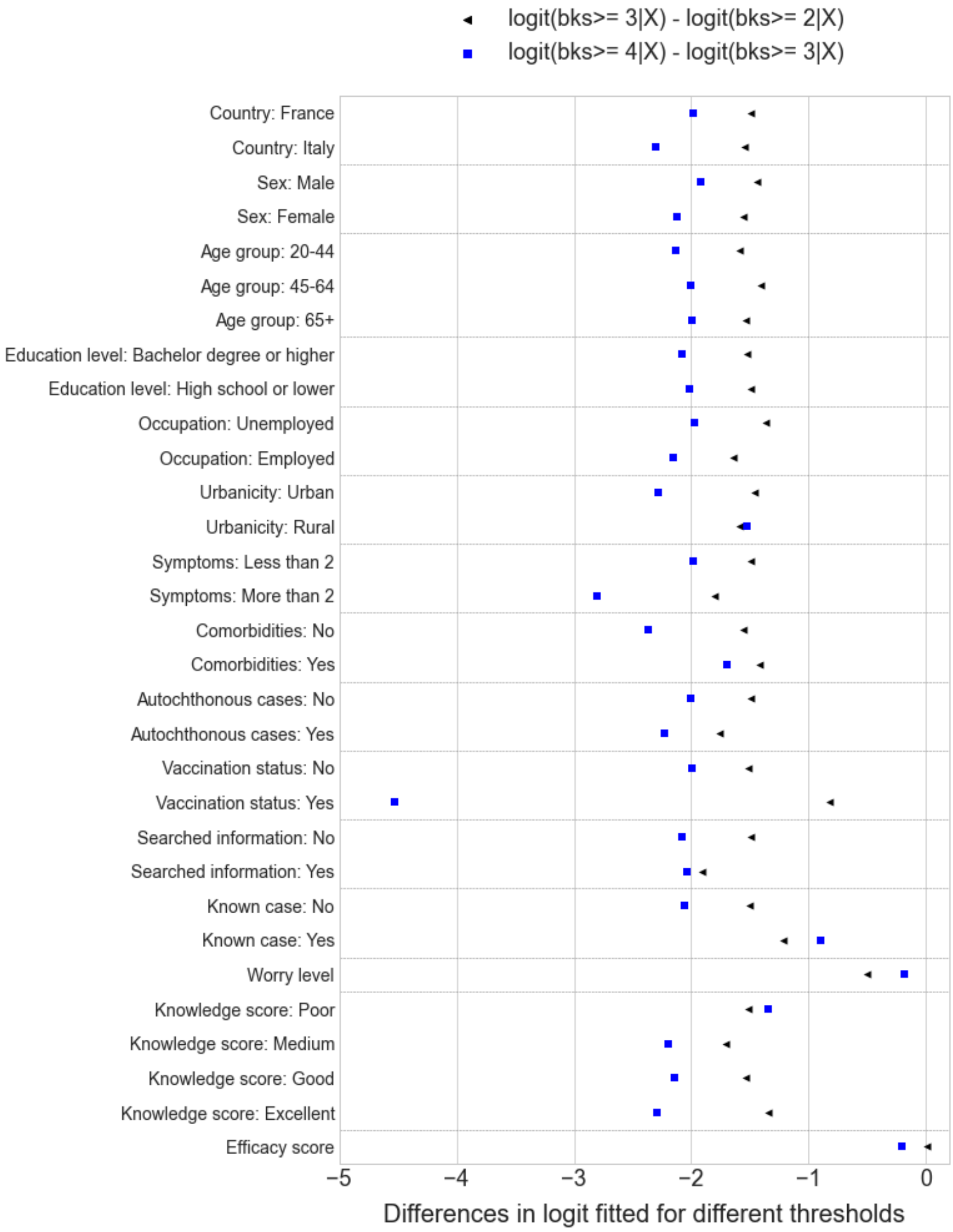

Figure D. Proportionality of odds of the number of measures taken. *Differences in fitted logits are shown for each explanatory variable considered. In the legend, X refers to the level considered for each explanatory variable.*

Variance Inflation Factor

As a check for multicollinearity among the variables in each model we computed the Variance Inflation Factor (VIF). In **Table F** we reported the values of VIF for the main outcomes: *Awareness of Dengue, Knowledge of characteristics, Adoption of measures and Willingness to be vaccinated*. In **Table G** we reported the values of VIF for the other two outcomes referring to the main reasons not to vaccinate: *I don't belong to any risk group* and

I don't think I'll get Dengue. All the values in both tables are lower than 4 with the sole exception of: *efficacy score* for the outcome *Adoption of measures* (VIF = 7.79), *occupation* for the outcomes *Willingness to be vaccinated* and *I don't belong to any risk group* (respectively, VIF = 4.37 and VIF = 4.21). Even those values are lower than 10 and we can conclude that the multicollinearity is not a concern in our analysis.

Categorical variable	Variable	Reference	VIF Awareness of dengue	VIF Knowledge of characteristics	VIF Adoption of measures	VIF Willingness to be vaccinated
Country	Italy	France	1.13	Excluded in VS	Excluded in VS	1.32
Sex	Female	Male	1.33	Excluded in VS	2.33	2.03
Age Group	20-44	65+	Excluded in VS	1.09	Excluded in VS	1.94
Age Group	45-64	65+	Excluded in VS	1.41	Excluded in VS	3.97
Education level	Until High School	Post High School	1.32	Excluded in VS	Excluded in VS	1.47
Occupation	True	False	Excluded in VS	Excluded in VS	1.79	4.37
Urbanicity	Rural	Urban	1.23	1.24	1.36	1.34
Symptoms	True	False	1.02	Excluded in VS	1.04	Excluded in VS
Comorbidities	True	False	Excluded in VS	1.29	1.53	Excluded in VS
Autochthonous cases	True	False	Excluded in VS	Excluded in VS	1.14	1.12
Vaccination status	True	False	NA	1.01	1.01	NA
Searched information	True	False	NA	1.20	1.30	1.29
Known case	True	False	NA	Excluded in VS	Excluded in VS	1.03
Worry level	Worry level	None	NA	1.93	2.69	2.38
Knowledge	Good	Excellent	NA	NA	2.15	1.65
Knowledge	Medium	Excellent	NA	NA	1.91	1.57
Knowledge	Poor	Excellent	NA	NA	1.65	1.39
Efficacy score	Efficacy score	None	NA	NA	7.79	NA
Self evaluation	Self evaluation	None	NA	NA	NA	Excluded in VS

Table F. Variance Inflation Factor of awareness of dengue, its characteristics, adoption of preventive measures, and vaccination willingness. *Variance Inflation factors for each variable of the four main outcomes analysed in the main model. The values refer to the final models after variable selection. NA means that the variable was not included in the initial list of possible explanatory variables, while "Excluded in VS" means that the variable was originally included but then discarded during variable selection.*

Categorical variable	Variable	Reference	VIF<br>I don't belong to any risk group	VIF<br>I don't think I 'll get Dengue
Country	Italy	France	1.16	1.15
Sex	Female	Male	Excluded in VS	Excluded in VS
Age Group	20-44	65+	1.78	Excluded in VS
Age Group	45-64	65+	3.90	Excluded in VS
Education level	Until High School	Post High School	1.49	Excluded in VS
Occupation	True	False	4.21	1.58
Urbanicity	Rural	Urban	Excluded in VS	1.32
Symptoms	True	False	Excluded in VS	1.02
Comorbidities	True	False	1.37	Excluded in VS
Autochthonous cases	True	False	Excluded in VS	Excluded in VS
Searched information	True	False	Excluded in VS	Excluded in VS
Known case	True	False	Excluded in VS	Excluded in VS
Worry level	Worry level	None	1.88	2.06
Knowledge	Good	Excellent	1.58	Excluded in VS
Knowledge	Medium	Excellent	1.48	Excluded in VS
Knowledge	Poor	Excellent	1.33	Excluded in VS
Self evaluation	Self evaluation	None	Excluded in VS	2.58

Table G. Variance Inflation Factor of the main reasons for vaccine refusal. *Variance Inflation factors for each variable of the two main reasons not to be vaccinated, analysed in the main model. The values refer to the final models after variable selection. NA means that the variable was not included in the initial list of possible explanatory variables, while "Excluded in VS" means that the variable was originally included but then discarded during variable selection.*

Interaction terms

In this section, we show the results of the bivariate regression analyses conducted to assess the homogeneity of the Italian and French datasets. In the figures below we report the ORs for the variables of France and Italy separately only if the interaction term among each variable and country is significant. Indeed, if the interaction terms are not significant, there

are no significant differences between the ORs of France and Italy and, thus, the results shown in the manuscript hold for both countries.

In **Figure E**, we show the ORs affected by the interaction terms for the outcome *Awareness of Dengue*. While *sex* and *urbanicity* are significantly associated with the outcome for France, as shown in the manuscript, this association is not significant for Italy. On the other hand, the level 20-44 of the variable *age group* was not found significant in the manuscript, but if we take into account the interaction with the country we observe that the variable is positively associated with the knowledge of dengue in France but not in Italy.

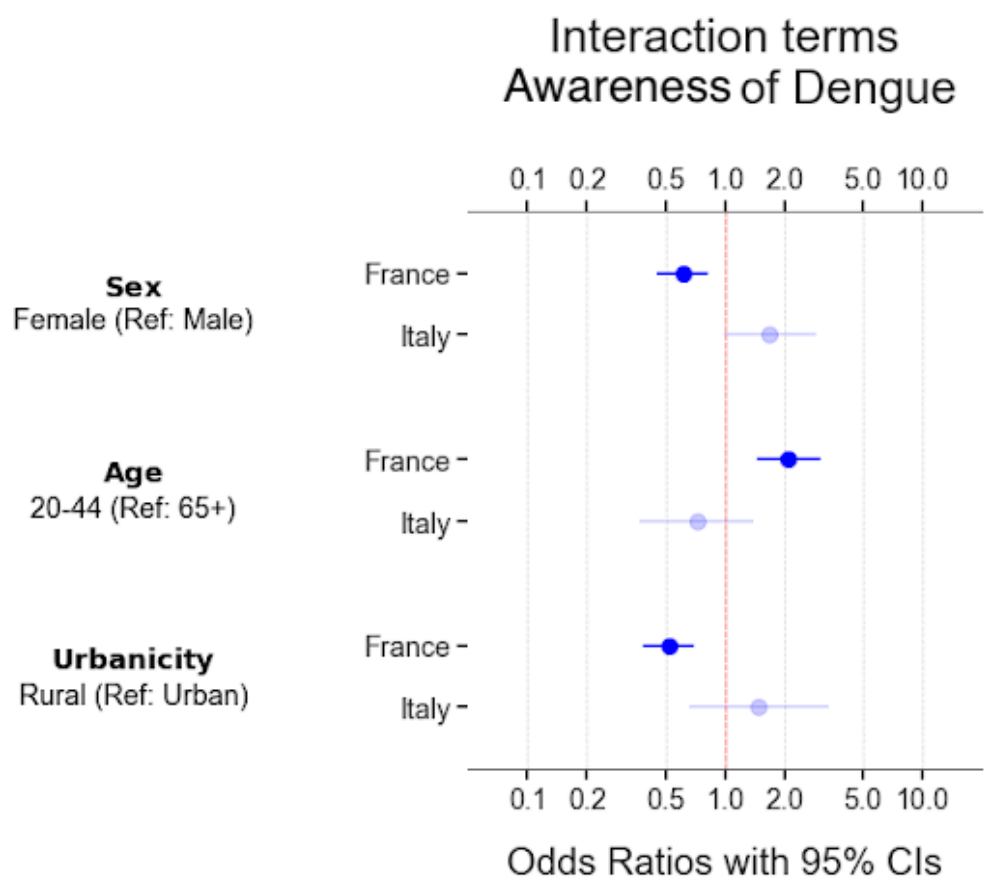

Figure E. Interaction terms of the outcome Awareness of Dengue. *Odds ratios (ORs) with 95% confidence intervals related to the outcome Awareness of Dengue for those variables that have significant interaction terms. Faded points represent non significant levels of included variables.*

In **Figure F**, we show the ORs affected by the interaction terms for the outcome *Knowledge of characteristics*. The significant associations of the variables *sex*, *autochthonous case, known case*, and *worry level* that we observe in the manuscript hold only for France and the variables are not significant for Italy. On the contrary, the variable *occupation* is significant only for Italy and not for France. Finally, the OR for *vaccination status* is not shown for Italy because in the italian dataset only two participants were vaccinated and, thus, it is not possible to properly compute an OR.

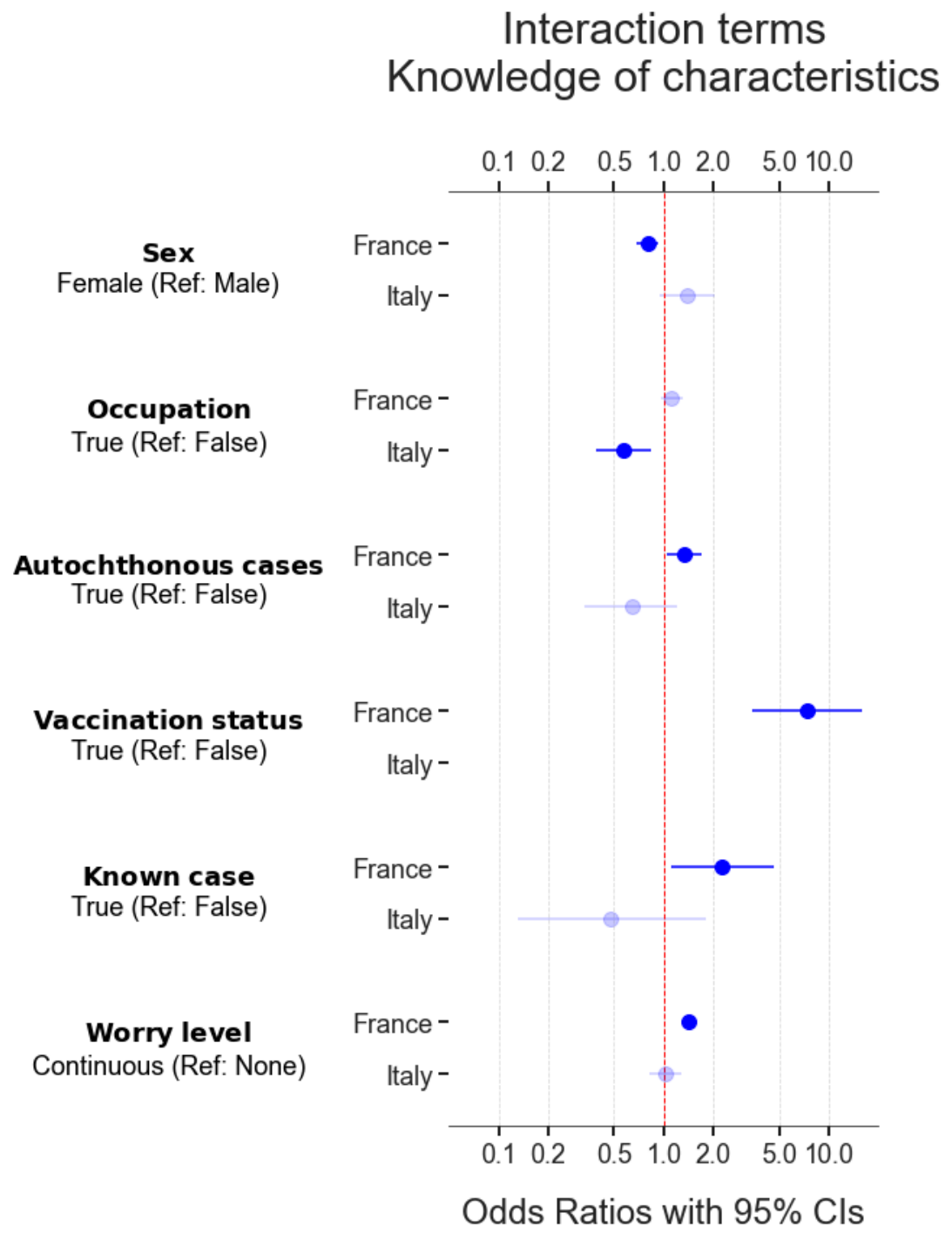

Figure F. Interaction terms of the outcome Knowledge of characteristics. *Odds ratios (ORs) with 95% confidence intervals related to the outcome Knowledge of characteristics for those variables that have significant interaction terms. Faded points represent non significant levels of included variables.*

In **Figure G**, we show the ORs affected by the interaction terms for the outcome *Adoption of measures*. The significant associations of the variables *autochthonous case* and *worry level* that we observe in the manuscript hold only for France and the variables are not significant for Italy. The variable *urbanicity* has two opposite associations in the two countries: the variable is positively associated with the outcome in France and negatively associated with the outcome in Italy. Finally, the ORs for *searched information* are significantly different in France and Italy but the association is positive for both countries, and slightly smaller for Italy.

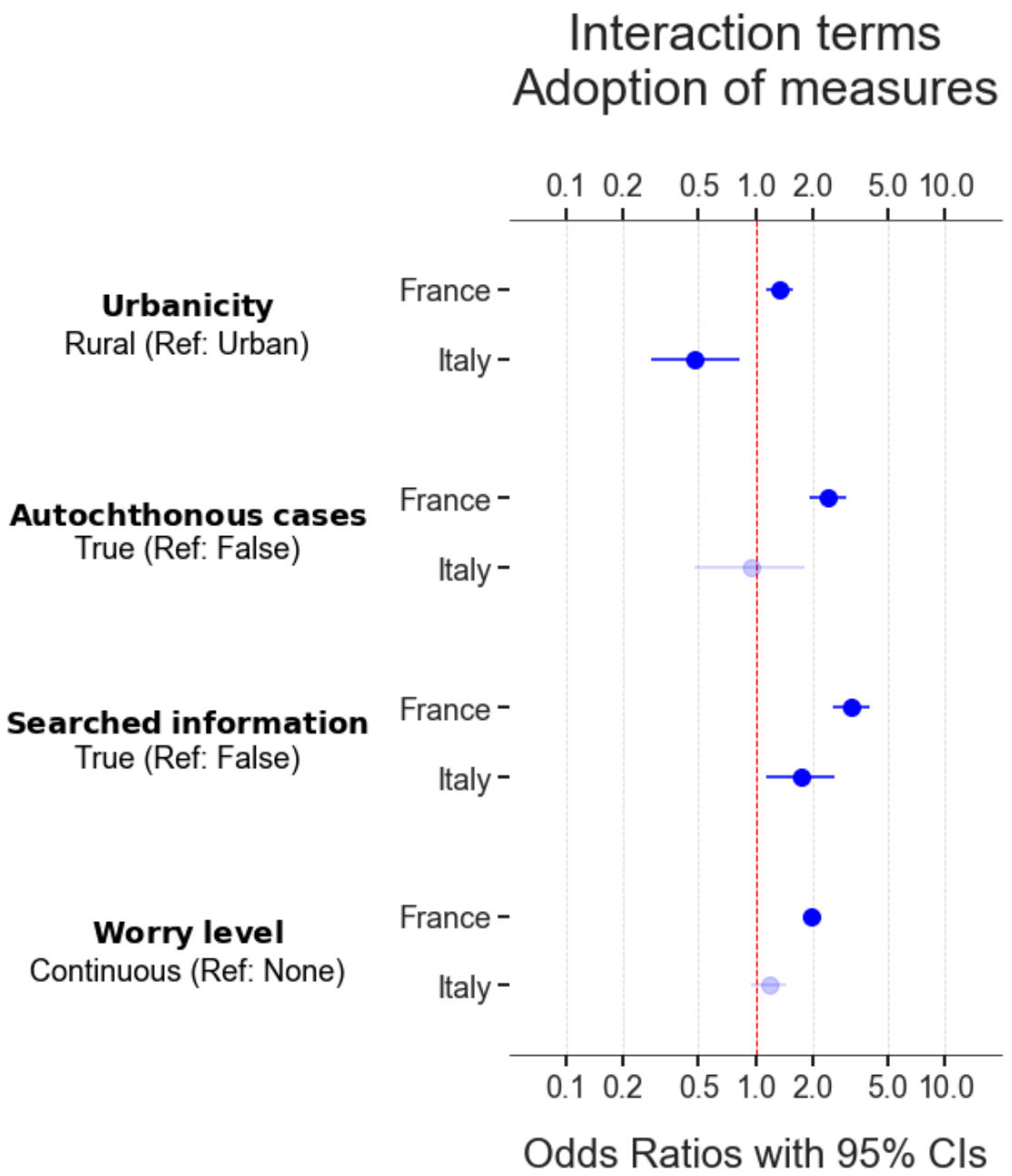

Figure G. Interaction terms of the outcome Adoption of measures. *Odds ratios (ORs) with 95% confidence intervals related to the outcome Adoption of measures for those variables that have significant interaction terms. Faded points represent non significant levels of included variables.*

In **Figure H**, we show the ORs affected by the interaction terms for the outcome *Willingness to be vaccinated*. The significant associations of the variables *sex*, *urbanicity*, and *searched information* that we observe in the manuscript hold only for France and the variables are not significant for Italy. On the contrary, the variable *occupation* is significant only for Italy and not for France. Finally, the "medium" level of the variable *knowledge score* has two opposite associations in the two countries: the variable is positively associated with the outcome in France and negatively associated with the outcome in Italy.

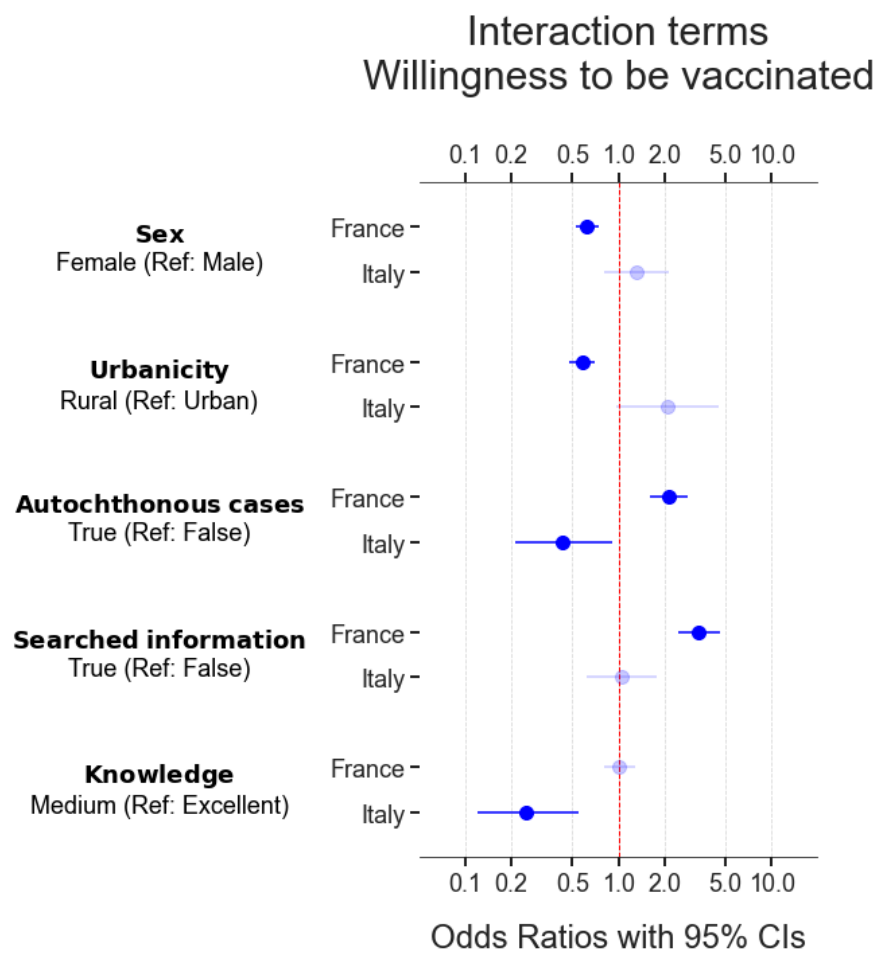

Figure H. Interaction terms of the outcome Willingness to be vaccinated. *Odds ratios (ORs) with 95% confidence intervals related to the outcome willingness to be vaccinated for those variables that have significant interaction terms. Faded points represent non significant levels of included variables.*

In **Figure I**, we show the ORs affected by the interaction terms for the two main reasons not to vaccinate: *I don't belong to any risk group* and *I don't think I'll get Dengue*. For *I don't belong to any risk group*, the significant association of the variable *comorbidity* that we observe in the manuscript holds only for France and the variable is not significant for Italy.

On the contrary, for *I don’t think I'll get Dengue*, we have two opposite associations for the variable *sex*: the variable is negatively associated with the outcome for France and positively associated for Italy.

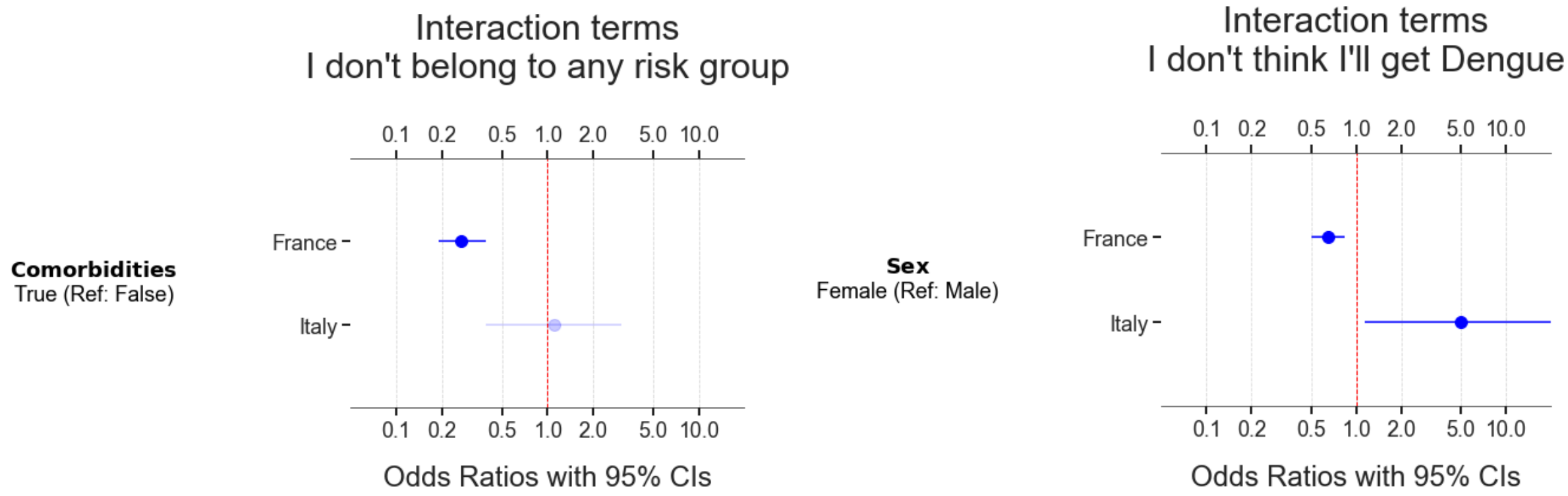

Figure I. Interaction terms of the main reasons for vaccine refusal. *Odds ratios (ORs) with 95% confidence intervals related to the two main reasons for vaccine refusal for those variables that have significant interaction terms. Faded points represent non significant levels of included variables.*

Sensitivity on the definition of symptom history

The variable *Symptoms* is defined in the main analysis as the experience of two or more symptoms from the list “*eye pain; blood in the feces; blood in the vomit; bloody gums; bone pain”* in the past thirty days. For sensitivity, we repeated the analysis considering instead the *Symptoms* variable defined by the experience of one or more symptoms and by three or more symptoms. Results of the statistical analysis are shown in **Figure J** and **Figure K** for the alternative definition based on at least one symptom, and **Figure L** and **Figure M** for the definition based on at least three symptoms.

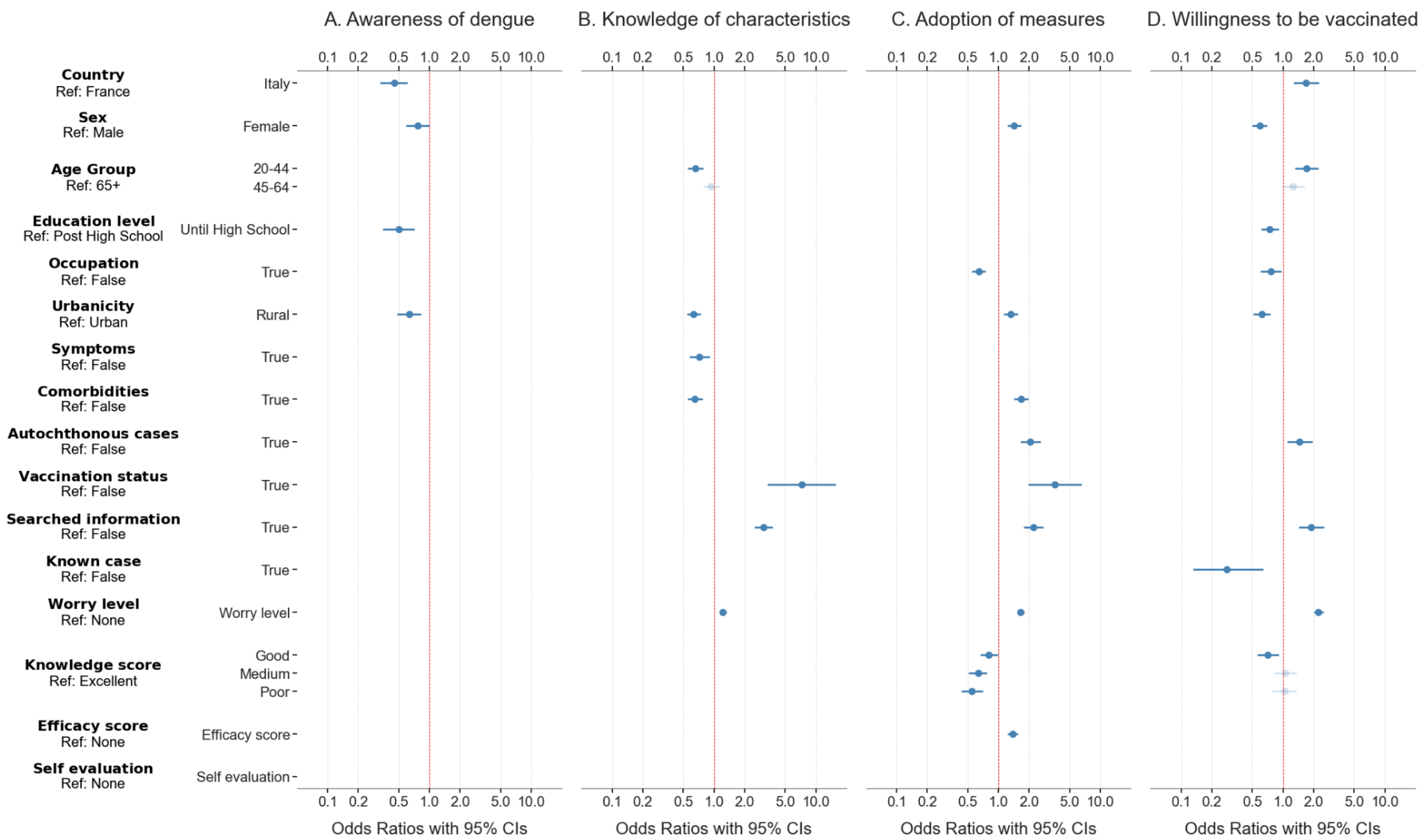

Figure J. Sensitivity on the symptoms variable definition of the determinants of knowledge of dengue, its characteristics, adoption of preventive measures, and vaccination willingness. A) *Determinants of the awareness of dengue identified with a logistic regression model in the form of odds ratios with 95% confidence intervals.* ***B)*** *Determinants of the knowledge of dengue characteristics, obtained with an ordinal regression model applied to the answers of the participants who have heard of dengue.* ***C)*** *Determinants associated with adoption of preventive numbers (obtained from an ordinal regression model, among participants who have heard of dengue).* ***D)*** *Determinants of willingness to be vaccinated (binary logistic model) among the participants who are unvaccinated. Models are multivariate, with variables selected as described in the Methods section. Variables without an associated OR for a given outcome were either not included as explanatory variables in the initial list of considered determinants, or were excluded during the variable selection process. Faded points represent non significant levels of included variables. All analyses are conducted on the pooled dataset including French and Italian samples. The symptoms variable is defined by the experience of at least one symptom.*

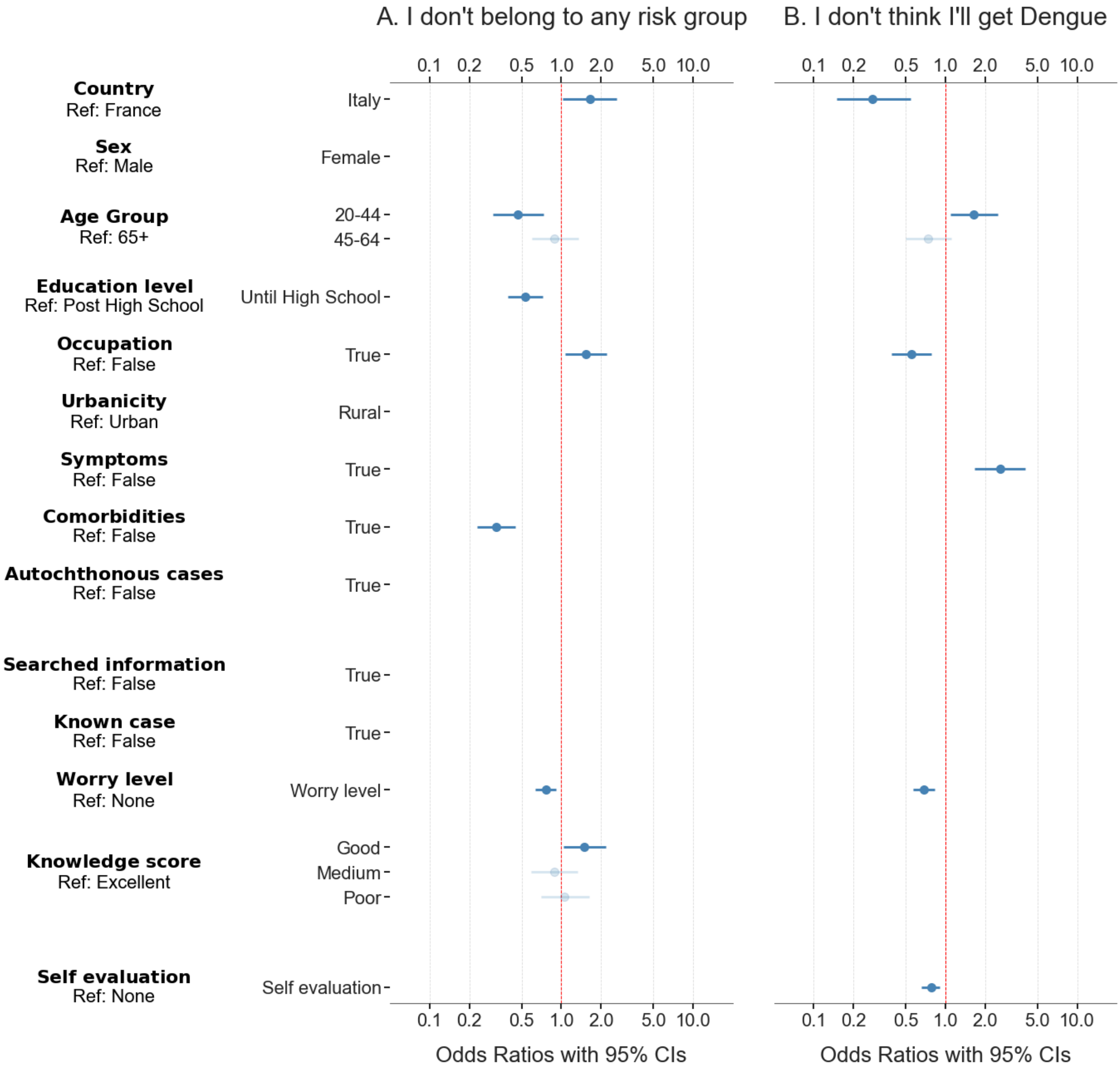

Figure K. Sensitivity on the symptoms variable definition of the determinants of the main reasons for vaccine refusal. A) *Determinants of citing "I don't belong to any risk group" in the form of odds ratios with 95% confidence intervals.* ***B)*** *Determinants of citing "I don't think I'll get dengue" in the form of odds ratios with 95% confidence intervals. Odds ratios were obtained from a multivariate, bias adjusted logistic regression model applied to the participants who are unwilling to be vaccinated. Variables without an associated OR were excluded during the variable selection process. Faded points represent non significant levels of included variables. All analyses are conducted on the pooled dataset including French and Italian samples. The symptoms variable is defined by the experience of at least one symptom.*

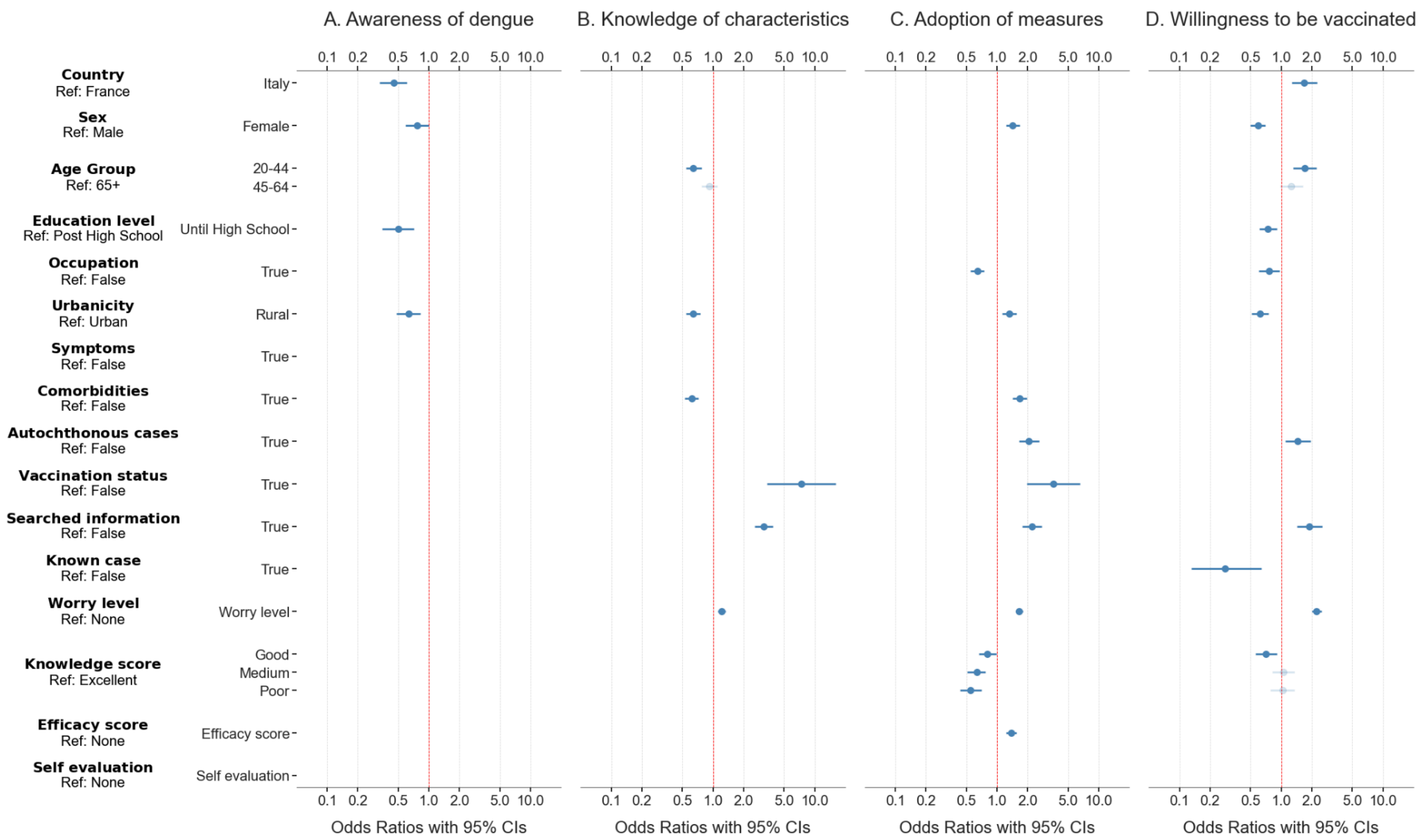

Figure L. Sensitivity on the symptoms variable definition of the determinants of knowledge of dengue, its characteristics, adoption of preventive measures, and vaccination willingness. A) *Determinants of the awareness of dengue identified with a logistic regression model in the form of odds ratios with 95% confidence intervals.* ***B)*** *Determinants of the knowledge of dengue characteristics, obtained with an ordinal regression model applied to the answers of the participants who have heard of dengue.* ***C)*** *Determinants associated with adoption of preventive numbers (obtained from an ordinal regression model, among participants who have heard of dengue).* ***D)*** *Determinants of willingness to be vaccinated (binary logistic model) among the participants who are unvaccinated. Models are multivariate, with variables selected as described in the Methods section. Variables without an associated OR for a given outcome were either not included as explanatory variables in the initial list of considered determinants, or were excluded during the variable selection process. Faded points represent non significant levels of included variables. All analyses are conducted on the pooled dataset including French and Italian samples. The symptoms variable is defined by the experience of at least three symptoms.*

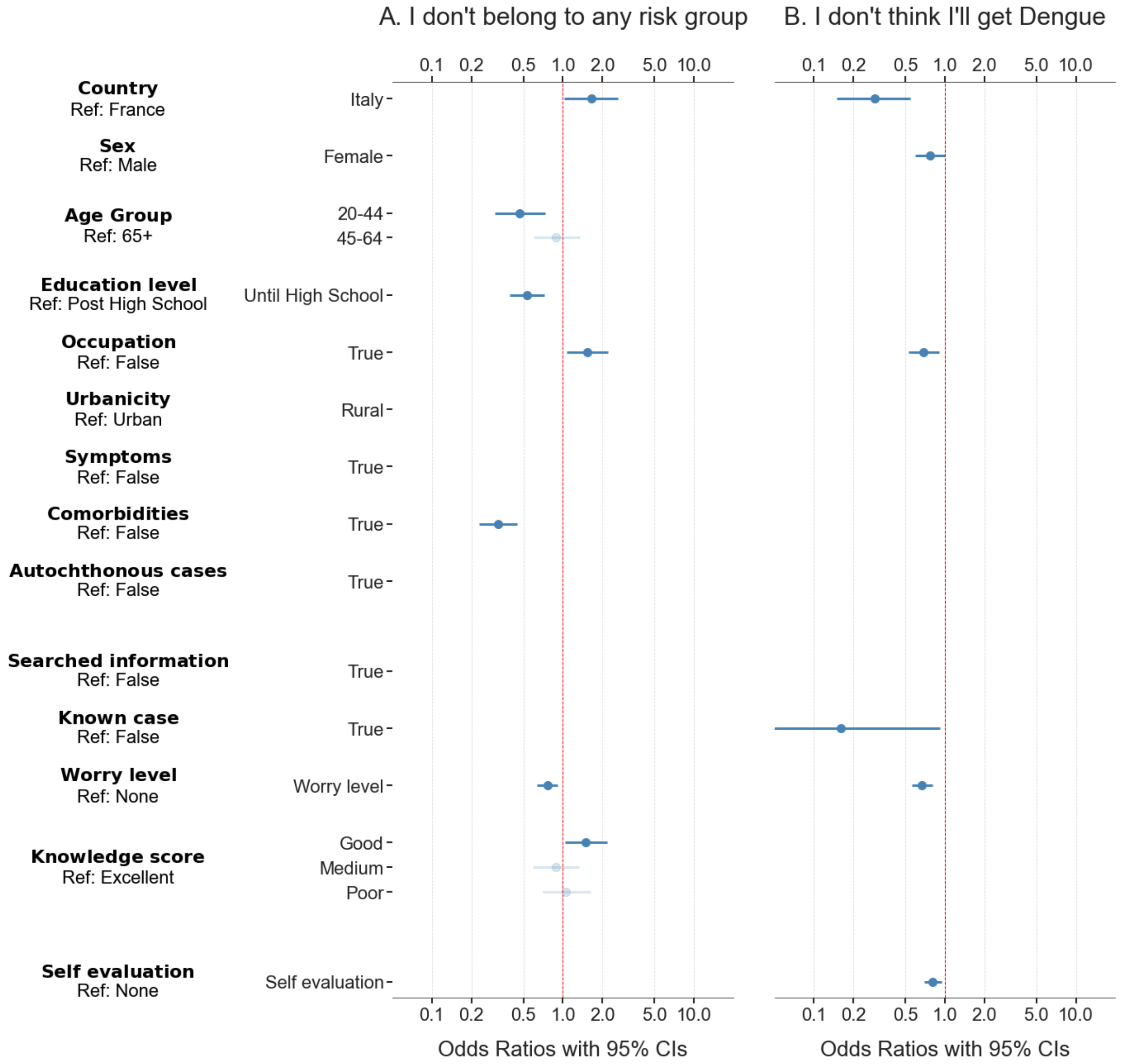

Figure M. Sensitivity on the symptoms variable definition of the determinants of the main reasons for vaccine refusal. A) *Determinants of citing "I don't belong to any risk group" in the form of odds ratios with 95% confidence intervals.* ***B)*** *Determinants of citing "I don't think I'll get dengue" in the form of odds ratios with 95% confidence intervals. Odds ratios were obtained from a multivariate, bias adjusted logistic regression model applied to the participants who are unwilling to be vaccinated. Variables without an associated OR were excluded during the variable selection process. Faded points represent non significant levels of included variables. All analyses are conducted on the pooled dataset including French and Italian samples. The symptoms variable is defined by the experience of at least three symptoms.*

Additional tables

We present in the following two tables (**Table H** and **Table I**) the data shown in Figures 4 and 5 of the main article.

Categorical variable	Variable	Reference	OR (95% CI) awareness	OR (95% CI) knowledge	OR (95% CI) adoption	OR (95% CI) willingness
Country (Ref:France)	Italy	France	0.46 (0.34-0.64)	Excluded in VS	Excluded in VS	1.69 (1.28-2.24)
Sex (Ref:Male)	Female	Male	0.76 (0.58-0.99)	Excluded in VS	1.43 (1.23-1.66)	0.59 (0.50-0.70)
Age Group (Ref: 65+)	20-44	65+	Excluded in VS	0.64 (0.54-0.77)	Excluded in VS	1.70 (1.30-2.23)
	45-64	65+	Excluded in VS	0.92 (0.77-1.10)	Excluded in VS	1.26 (0.97-1.64)
Education level	Until High School	Post High School	0.49 (0.35-0.69)	Excluded in VS	Excluded in VS	0.74 (0.61-0.91)
Occupation	True	False	Excluded in VS	Excluded in VS	0.63 (0.54-0.74)	0.76 (0.60-0.96)
Urbanicity	Rural	Urban	0.65 (0.49-0.86)	0.64 (0.54-0.75)	1.36 (1.15-1.61)	0.62 (0.51-0.75)
Symptoms	True	False	6.29 (1.41-28.00)	Excluded in VS	2.25 (1.59-3.20)	Excluded in VS
Comorbidities	True	False	Excluded in VS	0.62 (0.53-0.72)	1.52 (1.28-1.80)	Excluded in VS
Autochthonous cases	True	False	Excluded in VS	Excluded in VS	2.13 (1.69-2.68)	1.46 (1.10-1.94)
Vaccination status	True	False	NA	7.40 (3.41-16.03)	3.74 (2.05-6.83)	NA
Searched information	True	False	NA	3.17 (2.59-3.87)	2.33 (1.88-2.88)	1.90 (1.43-2.53)
Known case	True	False	NA	Excluded in VS	Excluded in VS	0.28 (0.13-0.64)
Worry level	Worry level	None	NA	1.21 (1.12-1.32)	1.63 (1.50-1.78)	2.23 (1.99-2.49)
Knowledge	Good	Excellent	NA	NA	0.78 (0.64-0.96)	0.71 (0.56-0.91)
Knowledge	Medium	Excellent	NA	NA	0.61 (0.50-0.75)	1.05 (0.82-1.35)
Knowledge	Poor	Excellent	NA	NA	0.55 (0.43-0.69)	1.03 (0.78-1.35)
Efficacy score	Efficacy score	None	NA	NA	1.36 (1.22-1.53)	NA
Self evaluation	Self evaluation	None	NA	NA	NA	Excluded in VS

Table H. Determinants of knowledge of dengue, its characteristics, adoption of preventive measures, and vaccination willingness. *Determinants of the awareness of dengue identified with a logistic regression model, knowledge of dengue characteristics, obtained with an ordinal regression model applied to the answers of the participants who have heard of dengue, adoption of preventive numbers (obtained from an ordinal regression model, among participants who have heard of dengue) and of willingness to be vaccinated (binary logistic model) among the participants who are unvaccinated are shown in the forms of OR and 95% CIs. Models are multivariate, with variables selected as described in the main manuscript. All analyses are conducted on the pooled dataset including French and Italian samples.*

Categorical variable	Variable	Reference	OR (95% CI) I don't belong to any risk group	OR (95% CI) I don't think I'll get dengue
Country	Italy	France	1.64 (1.02-2.63)	0.34 (0.18-0.64)
Sex	Female	Male	Excluded in VS	Excluded in VS
Age Group	20-44	65+	0.47 (0.30-0.73)	Excluded in VS
Age Group	45-64	65+	0.89 (0.60-1.34)	Excluded in VS
Education level	Until High School	Post High School	0.53 (0.39-0.72)	Excluded in VS
Occupation	True	False	1.54 (1.07-2.22)	0.61 (0.47-0.80)
Urbanicity	Rural	Urban	Excluded in VS	1.36 (1.02-1.80)
Symptoms	True	False	Excluded in VS	12.89 (5.58-29.80)
Comorbidities	True	False	0.32 (0.23-0.45)	Excluded in VS
Autochthonous cases	True	False	Excluded in VS	Excluded in VS
Vaccination status	True	False	NA	NA
Searched information	True	False	Excluded in VS	Excluded in VS
Known case	True	False	Excluded in VS	Excluded in VS
Worry level	Worry level	None	0.76 (0.63-0.91)	0.65 (0.54-0.78)
Knowledge	Good	Excellent	1.50 (1.03-2.19)	Excluded in VS
Knowledge	Medium	Excellent	0.88 (0.59-1.32)	Excluded in VS
Knowledge	Poor	Excellent	1.06 (0.70-1.62)	Excluded in VS
Efficacy score	Efficacy score	None	NA	NA
Self evaluation	Self evaluation	None	Excluded in VS	0.73 (0.62-0.85)

Table I. Determinants of the main reasons for vaccine refusal. *Determinants of citing "I don't belong to any risk group" and "I don't think I'll get dengue" are shown in the form of odds ratios with 95% confidence intervals. Odds ratios were obtained from a multivariate, bias adjusted logistic regression model applied to the participants who are unwilling to be vaccinated. All analyses are conducted on the pooled dataset including French and Italian samples.*

References

1. Liddell TM, Kruschke JK. Analyzing ordinal data with metric models: What could possibly go wrong? J Exp Soc Psychol. 2018 Nov 1;79:328–48.

2. Rodríguez G. Chapter 6: Multinomial Response Models [Internet]. 2007. Available from: https://grodri.github.io/glms/notes/

3. How do I interpret the coefficients in an ordinal logistic regression in R? | R FAQ UCLA: Statistical Consulting Group. [Internet]. [cited 2025 Mar 14]. Available from: https://stats.oarc.ucla.edu/r/faq/ologit-coefficients/

4. Bürkner PC, Vuorre M. Ordinal Regression Models in Psychology: A Tutorial. Adv Methods Pract Psychol Sci. 2019 Mar 1;2(1):77–101.

5. Ordinal Logistic Regression| R data analysis examples. UCLA: Statistical Consulting Group. [Internet]. [cited 2025 Mar 14]. Available from: https://stats.oarc.ucla.edu/r/dae/ordinal-logistic-regression/

6. Harrell , FE. Regression Modeling Strategies: With Applications to Linear Models, Logistic and Ordinal Regression, and Survival Analysis [Internet]. Cham: Springer International Publishing; 2015 [cited 2025 Mar 14]. (Springer Series in Statistics). Available from: https://link.springer.com/10.1007/978-3-319-19425-7
