# Supplementary material for: Assessing dengue knowledge, attitudes and preventive practices in France and Italy: A cross-sectional study using participatory surveillance cohorts
Source: PLOS Glob Public Health. 2026 Sep 23;6(9):e0007162. doi: 10.1371/journal.pgph.0007162 (PMC13600492; doi:10.1371/journal.pgph.0007162)
Supplement: Supplementary file 2 [file pgph.0007162.s003.pdf]

Lucille Calmon et al., in their manuscript intitled “***Assessing dengue knowledge, attitudes and preventive practices: a multicountry digital cohorts study***”, leveraged French and Italian participatory surveillance cohorts from the European network *InfluenzaNet* to investigate emerging threats unrelated to respiratory viruses. They collected data on knowledge, attitudes, and preventive practices related to dengue in France and Italy, where dengue is an emerging threat. Their findings revealed high awareness, moderate levels of concern, and moderate willingness to be vaccinated, highlighting the urgent need for communication campaigns to strengthen knowledge and awareness of these lesser-known aspects.

This study is dense, highly relevant, and of significant public health importance, as it allowed the authors to analyze participants’ knowledge, attitudes, behaviors, and practices related to dengue in a non-endemic context such as Europe.

I believe this study is suitable for consideration by *PLOS Global Public Health* for publication.

The manuscript is well written and easy to read. However, I have a few comments that could help the authors further improve this important work:

Specific Comments

Line 21: Please add *“worldwide”* at the end of the sentence.

Lines 66–67: Add *“the”* before *assessment* in the sentence *“In 2025, guidance from the ECDC on assessment…”* and delete the second *“of Aedes-borne”*.

Line 68: Replace *“faces high numbers of importations of dengue cases in”* with *“reports large numbers of imported dengue cases into”*.

Line 71: Replace *“dengue importations”* with *“imported dengue cases”* and *“came from”* with *“originated from”*.

Line 72: Elaborate further (why) on the statement *“the dengue vaccine Qdenga was approved in Europe in 2022, but is not recommended in mainland France.”*

Line 81: Replace *“benefits gained from the”* with *“perceived benefits of”*.

Lines 93–96: Specify the country/countries where these observations were made.

Lines 107–111: The sentence is too long. Consider splitting it into two and replacing *“but”* with *“However”*. Also revise as follows:

- *“in a sample randomly selected in colonised departments”* to *“in a randomly selected sample from colonized departments”*
- *“found an increasing awareness of the mode of transmission of dengue”* to *“revealed increasing awareness of dengue transmission”*
- *“knowledge of other mosquito-borne viruses characteristics”* to *“knowledge of the characteristics of other mosquito-borne viruses”*

Line 159: Replace:

- *“between”* by *“from”*
- *“The data was”* by *“Data were”*
- *“collected to capture”* by *“intended to capture”*
- *“responses filled out until”* by *“responses submitted until”*

Statistical analysis:

- Rephrase *“ordinality of the different levels of knowledge and measure adoption”* as *“the ordered nature of knowledge levels and preventive measure adoption”*.
- Avoid repeating *“analyses were conducted”*.
- Change *“called in python with r2py”* to *“accessed in Python via rpy2”*.

Explanatory variables:

- Avoid repetition of *participants’ intake survey*.
- Change *“education attainment level”* to *“educational attainment”*.
- Change *“the presence of at least one comorbidity”* to *“presence of at least one comorbidity”*.

Line 277: Clarify what other commonly reported reasons for refusing vaccination were. It is known that in France, “historical mistrust” and “fear of side effects” are among the leading reasons of refusing vaccination…

Ethics approval and consent to participate:

- *“In France, the Grippenet/Covidnet study was approved, and in Italy, the influweb.it study was conducted in accordance with the Italian Data Protection Authority. However, as these approvals were initially not intended for research on dengue or other diseases, it is expected that an approved addendum should be provided by the authors, stipulating that the authorized cohorts may also be used for additional studies.”*

Results:

- Change *“while the French sample was biased towards females”* to *“whereas the French sample was skewed toward females”*.

Fear and vaccination:

- It would be useful to know the other most frequently cited reasons for refusing vaccination if offered.

Discussion (Lines 699–701):

- I do not agree with the authors’ assumption that future vaccines against dengue would be well received in France and Italy based the refusal reasons I cited above. Si please elaborate further on this.
