# Supplementary material for: Assessing dengue knowledge, attitudes and preventive practices in France and Italy: A cross-sectional study using participatory surveillance cohorts
Source: PLOS Glob Public Health. 2026 Sep 23;6(9):e0007162. doi: 10.1371/journal.pgph.0007162 (PMC13600492; doi:10.1371/journal.pgph.0007162)
Supplement: Supplementary file 3 [file pgph.0007162.s005.pdf]

Assessing dengue knowledge, attitudes and preventive practices in France and Italy: a cross-sectional study using participatory surveillance cohorts R1, by Lucille Calmon et al.

I would like to thank the authors for their detailed and thoughtful responses to my comments and to other reviewers' comments. I appreciate the clarifications provided, particularly regarding the recommendations for the Qdenga vaccine in mainland France, the specification of refusal reasons for vaccination, and the ethical approval framework in France and Italy.

The revisions made to the abstract, introduction, methodology, and results sections have significantly improved the clarity and accuracy of the manuscript. I also acknowledge the careful rephrasing of several sentences, which has enhanced readability and scientific precision.

Overall, I am satisfied with the authors' responses and the changes implemented. I agree that the manuscript has been strengthened and is now more suitable for publication in PLOS Global Public Health.
