# Supplementary material for: Assessing dengue knowledge, attitudes and preventive practices in France and Italy: A cross-sectional study using participatory surveillance cohorts
Source: PLOS Glob Public Health. 2026 Sep 23;6(9):e0007162. doi: 10.1371/journal.pgph.0007162 (PMC13600492; doi:10.1371/journal.pgph.0007162)
Supplement: Supplementary file 4 [file pgph.0007162.s007.pdf]

Reviewer #1:

Overall Impression

The manuscript "Assessing dengue knowledge, attitudes and preventive practices: a multicountry digital cohorts study" addresses a timely and important public health question: how populations in mainland France and Italy perceive dengue risk and adopt preventive behaviours as Aedes vectors expand across Europe. The study leverages established participatory surveillance cohorts (InfluenzaNet) and applies bias-adjusted regression models to a large pooled sample (2,904 participants) to identify determinants of awareness, knowledge, adoption of preventive behaviour, and vaccine willingness. The work is relevant to preparedness and risk communication strategies in non-endemic settings and offers useful empirical evidence for public health planning.

Strengths

• Timeliness and relevance. The topic is highly relevant given the documented expansion of Aedes mosquitoes and recent autochthonous dengue cases in Europe.

• Use of existing participatory cohorts. Leveraging InfluenzaNet cohorts is an efficient approach that enables rapid data collection and links to rich participant profiles.

• Large pooled sample. The combined sample size (France + Italy) provides statistical power to detect associations across multiple outcomes.

• Appropriate analytic approach. The use of bias-adjusted logistic and ordinal regression models, post-stratification by age/sex/education, and assessment of proportional odds assumptions are appropriate for the outcomes described.

• Policy oriented outcomes. The manuscript examines actionable endpoints (knowledge, preventive practices, and vaccine willingness) that can inform communication and intervention strategies.

• Clear presentation of variables. Table 1 and the methods section clearly list explanatory and outcome variables, facilitating reproducibility.

We thank the reviewer for the time they dedicated to the evaluation of our manuscript, and their positive feedback, as well as their numerous comments. We have taken them into careful consideration in order to improve our manuscript.

Areas for Improvement

1. Sampling, Representativeness and Generalisability

• 1.1 Clarify recruitment and response rates. The manuscript should report invitation numbers, response rates, and any differences between respondents and non respondents to assess selection bias.

We thank the reviewer for raising this point. Computing recruitment and response rates is not straightforward due to the inherent specificities of participatory surveillance. We hereby provide details, and proxy estimates.

Recruitment to the platforms Grippenet/Covidnet and InfluWeb are driven by several processes including word of mouth, communication campaigns, newspaper and radio interviews as well as social-media promotion. Registration to the platforms is entirely voluntary, as is participation in regular and punctual surveys. The invitation process depended on the platforms. All registered participants in Italy were sent monthly invitations to fill in the questionnaire studied in this survey, between May 13, 2024 and October 27,

2024. In France all registered participants were able to fill in the survey upon logging in, and the participants signed up to the platforms' newsletter were invited to participate once, on the 29th of July.

By design of the participatory surveillance systems, which are not based on active invitation, invitation numbers and recruitment rates are not appropriate metrics. However, we obtained a response rate to the questionnaire by comparing the samples of non-respondents and respondents among the registered participants. Overall, 6252 and 994 registered users respectively for the Grippenet/Covidnet and InfluWeb platforms could fill in the questionnaire, leading to a crude response rate of 43% for both platforms. However, note that registered participants may have registered years before and may not be actively responding to the platform in recent seasons, hence the numbers of non-respondents include registered participants no longer active on the platform. A more informative statistic is obtained by reporting the response rates based on the active participants during the prior Influenza season, in order to compare those users who are typically active on the platforms to those that filled in this questionnaire. In the year prior to the survey (corresponding to the 2023-2024 Influenza season), a total of 4856 and 585 users were respectively active on the platforms Grippenet/Covidnet and InfluWeb. This translates into 56% and 69% response rates for the two countries, respectively.

We have reported these details and statistics in the Results section and have provided in the Supplementary Materials descriptive statistics (Table S1) of the active users in the Influenza season of 2023-2024 who did not fill in the Dengue survey. This analysis demonstrates that respondents and active non-respondents differ in age and occupation distributions, with a smaller proportion of the respondents to the survey having an occupation, a larger proportion of the respondents being in older age groups compared to the active users. This is a consequence of the fact that older and unemployed individuals are more likely to engage with the platforms, and participate more frequently.

• 1.2 Limitations of participatory cohorts. InfluenzaNet participants are known to be older and more educated; while post stratification is used, discuss residual bias and how it might affect estimates of knowledge, worry, and behaviour.

We thank the reviewer for this comment. Indeed, participatory surveillance platforms are highly convenient and cost effective, but resulting samples are known to be biased towards more educated and older participants. Using post-stratification weights partly addresses this bias by recovering a sample representative of the census population in terms of age-sex-education distributions. The reviewer is however right that residual bias remain, primarily due to the fact that the participants are self-selected.

As participation is completely voluntary, participants may have a high interest in matters related to health, and be more likely to consider the adoption of protective measures (e.g. vaccination) positively. As detailed in the manuscript, previous studies have shown that "*For example, influenza vaccination coverage in InfluenzaNet cohorts is slightly higher than in the general population(79,80) while incidence trends of influenza-like illnesses obtained from InfluenzaNet cohorts are consistent with ECDC trends in terms of timing and magnitude(79). Individual risk factors for influenza-like illnesses obtained from InfluenzaNet cohorts have*

also been demonstrated to be consistent with the literature(79).” In line with this understanding, the self selection bias of participatory surveillance may lead to raised knowledge levels, and slightly higher adoption rates of preventive behaviours. Precise estimates of the impacts of residual bias on knowledge, worry, and behaviours is beyond the scope of the study, as it would require comparison with unbiased samples, e.g. via active recruitment strategies, which are highly costly to obtain.

We have now expanded the limitations of the manuscript to make this point precise.

• 1.3 Pooling rationale. Provide more detail and justification for pooling France and Italy (e.g., tests of homogeneity, country interaction terms). If important country-level differences exist, consider presenting stratified results or country-by-predictor interactions.

We thank the reviewer for raising this point. As observed by the reviewer, the pooling of the two datasets allows us to gain a higher statistical power to detect significant associations across the population groups. This is extremely important, especially considering the lower sample sizes of Italy. In the Results section, subsection *3.2.6 Interactions between the country of residence and the identified determinants*, we discuss the inclusion of interaction terms in the models to account for country-specific effects and report the few significant differences between the two countries. “*In the majority of the determinants considered, interaction terms with the country of residence were not significant, indicating that the determinant's effects apply independently of countries. Significant interactions were identified in a few cases, highlighting few cross country differences (see Supplementary Materials in S1 Text). Among Italian participants, those with an occupation were less likely to know a higher number of characteristics than those without, whereas in France employment status did not correlate with knowledge. While the main analysis shows that urbanicity was negatively associated with adopted measures, this reversed in Italy. A similar effect was measured in the case of the association between areas where autochthonous cases had been reported, and willingness to be vaccinated. The first effect may reflect structural differences in the rural–urban context between the two countries, while the second could be due to the small number of distinct areas with autochthonous cases in Italy in 2023. Finally, while sex was not associated with “I don’t think I'll get dengue” in the joint analysis, female participants were less likely than males to report this reason in France, but instead more likely in Italy.*”

2. Measurement and Construct Validity
2.1 Knowledge and behaviour scales. Provide psychometric information (e.g., internal consistency, item response distributions) for the Knowledge score, Efficacy score, and Worry level. Report how missing items were handled.

We thank the reviewer for this constructive methodological point. To align with our primary regression analysis, we evaluated the internal consistency of our composite scales using the weighted Cronbach's alpha. The 'Worry level' and 'Efficacy score' demonstrated excellent and good internal consistency (weighted alpha = 0.915 and 0.814, respectively). Furthermore, while the 'Knowledge score' is primarily a formative cumulative index, it also demonstrated acceptable internal consistency (weighted alpha = 0.773). These details have been added in the manuscript in the *Explanatory variables* subsection 2.5 of the methods.

Regarding the item-level response distributions, this data is detailed within our Results section, where we analyze the specific frequencies for individual worries, efficacy measures, and knowledge items. A review of these distributions, together with the text of the survey (available in the Supplementary Materials) demonstrates a lack of response set bias; for instance, the lowest Likert point was highly selected for Worry items (question qd5) but not for Efficacy items (question qd7). Moreover, the second most recognized dengue characteristic was presented fifth in the survey list (question qd4), confirming participants actively evaluated each item rather than defaulting to top-of-list options. Finally, there were no missing data for these specific items, as responses to these questions were mandatory for participants to progress in the digital survey.

2.2 Outcome definitions. The binary Symptoms variable is defined by a set of rare symptoms (e.g., blood in feces/vomit). Explain the rationale for this definition and report its prevalence; consider sensitivity analyses using alternative symptom definitions.

We thank the reviewer for the comment. Our rationale for this specific definition was driven by the need to maximize epidemiological specificity, in order to assess the impact of experiencing dengue-specific symptoms on knowledge, attitudes and practices. Utilizing broad, non-specific symptoms (e.g., fever, headache, cough) would obscure the findings due to the prevalence of other circulating respiratory and gastrointestinal viruses manifesting these symptoms. Therefore, we restricted our definition of symptom history to symptoms that are highly linked to dengue fever (such as retro-orbital eye pain and severe bone pain) and signs of severe dengue (hemorrhagic symptoms). This resulted in a multiple choice list of five symptoms (*eye pain, blood in the feces, blood in the vomit, bloody gums, and bone pain*). The most commonly reported symptom was eye pain (4.93% of the participants who filled the survey), followed by bone pain (4.35%), bloody gums (3.27%), blood in the feces (1.75%) and blood in the vomit (0.19%). Overall, 10.19% of the participants reported experiencing one or more symptoms in the past thirty days, 4.11% reported at least two symptoms, and 0.19% reported experiencing three or more symptoms.

The binary variable *Symptoms* was then defined by requiring the presence of at least two of these five symptoms to further avoid misclassification from isolated symptoms. We have now considered for sensitivity two alternative definitions of the variable *Symptoms*, requiring the presence of at least one symptom, and at least three symptoms respectively. Using these alternative variable definitions instead for our regression analyses, the statistical significance of the variable *Symptoms* changes in some of the outcomes: in particular, in our main analysis, the variable *Symptoms* (based on >=2 symptoms) is significant for the outcomes *Awareness of dengue*, *Adoption of measures* and *I don't think I will get dengu*e as vaccine refusal reason. With the definition based on >=1 symptoms, the variable becomes non-significant in the model for *Awareness of dengue* and *Adoption of measures*, but becomes significant for the outcome *Knowledge of dengue characteristics*. When using the definition based on >=3 symptoms, the symptom history becomes non-significant in all instances. The remainder of the findings are robust to these changes, with the exception of changes in statistical significance for determinants close to the significance threshold of the outcome *I don't think I'll get Dengue*.

We have now explicitly clarified our rationale for the definition of the *Symptoms* variable in the Methods section. We moreover reported the exact prevalence of each symptom, as well as the prevalence of one or more, two or more, and three or more symptoms in the Results

section. We included the alternative regression models obtained with the variable symptoms defined from at least one symptom in the Supplementary Materials (Figures S9-S10). In the results section, we have also now detailed the sensitivity of the results on the changes in symptom history definition. Finally, we have expanded the limitations of the manuscript to highlight these aspects.

2.3 Self reported vaccination. Given the low reported vaccination prevalence (0.4%), discuss potential misclassification (recall, misunderstanding of vaccine type) and whether respondents were given vaccine brand examples.

We thank the reviewer for raising this point. In the survey, respondents were asked '*Did you get the vaccine against Dengue? (Yes/No)*' without the provision of specific vaccine brand names (e.g., Qdenga). While we acknowledge the theoretical possibility of misclassification, the dengue vaccine is a highly specific and non-routine vaccine in France and Italy that typically requires a dedicated medical consultation and expense. In particular, as detailed in the manuscript, the vaccine is currently not available in mainland France. *"In the French overseas departments where dengue is circulating, it is recommended to children with a previous infection, and vulnerable adults. In Italy, it is recommended to travelers to endemic areas, for long (at least three weeks) or repeated trips, especially if the traveler has been previously infected with dengue."* Consequently, individuals who received the vaccine would have received it in a highly specific context, and are likely to recall it accurately without needing brand examples. The low reported vaccination prevalence is therefore in line with the broader context.

3. Statistical Analysis and Reporting
3.1 Model selection transparency. The manuscript mentions backward stepwise selection; report the selection criteria (p value thresholds, information criteria) and whether results are robust to alternative model specifications (e.g., full models, LASSO).

We thank the reviewer for highlighting the importance of transparency in model selection. These details are central to our methodology and are explicitly detailed in the manuscript. Specifically, the '*Variable selection*' section outlines our criteria: variables were initially screened via univariate analysis retaining those with $p < 0.2$, followed by an iterative backward elimination until all remaining predictors achieved $p \leq 0.05$. We evaluated the robustness of the variable selection method by considering all possible multivariate models, and selecting the one with the highest adjusted pseudo R^2, as detailed in the same section and the Supplementary Materials.

We additionally considered full models for each outcome, i.e. binomial/ordinal regression models including all explanatory variables (no variable selection at all). The model results are reported in the Supplementary Materials (Figures S1 and S2), and show overall good agreement with the models presented in the main manuscript. In particular, the variables that were discarded in our main models in the variable selection process are not statistically significant, with the following few exceptions:

- For *Awareness of Dengue*, the variable *sex* is non-significant in the full model, while it is in our main model (with variable selection).

- For *Knowledge of Characteristics*, the variable *Country* is significant in the full model, but is removed by the backward stepwise selection.
- For *I don't belong to any risk group*, the country variable is not significant while in our main model it is (although the significance is close to the threshold).
- For *I don't think I'll get Dengue*, the variables *education level* and the lowest knowledge score (poor) are significant while they weren't in our main model.

We also considered alternative model specifications by including a Ridge regularisation term, and comparing the resulting effects observed.
Models obtained for the outcomes *Awareness of dengue*, *Willingness to vaccinate*, and the two refusal reasons *I don't think I'll get dengue* and *I don't belong to any risk groups* with Ridge regularisation are compared in the Supplementary Materials to the models presented in the study (Tables S2-S5). In all instances, the effects observed and discussed in the main text are robust to alternative model specification with Ridge regularisation.

The remaining two outcomes (*Knowledge of dengue characteristics* and *adoption of measures)* are inherently ordinal, and were modelled with an ordinal regression model including post-stratification weights to recover age-sex-education representativeness. Implementations of ordinal regression models accounting for post-stratification weights do not support regularisation penalties. Similarly, existing implementations of ordinal regression models with regularisation penalties cannot take into account post-stratification weights in the effects estimates. In order to test the robustness of our results obtained from ordinal regression models to alternative models with regularisation, we considered ordinal models for the outcomes *Knowledge of dengue characteristics* and *adoption of measures* with a Ridge regularisation term. These models do not account for the post stratification weights, but despite this, the results obtained are compatible with the findings presented in the main manuscript. The results of these models are shown below. For comparison, we also show the results of (unregularised) ordinal regression models for these two outcomes without post stratification-weights. This strategy ensures that the absence of weights does not confound or mask the isolated effect of the regularisation penalty. The results of this comparison show agreement across all identified effects, demonstrating that our findings remain robust.

Categorical Variable	Variable	Reference	OR regularised (λ=0.0622)	OR unregularised	P-values unregularised	Agreement of direction	Shrinkage
Age Group	20-44	65+	0.78	0.68	0.001	True	0.67
Age Group	45-64	65+	0.93	0.85	0.029	True	0.49
Urbanicity	Rural	Urban	0.93	0.91	0.260	True	0.85
Comorbidities	True	False	0.99	0.92	0.292	True	0.19
Vaccination status	True	False	1.50	1.59	0.408	True	0.84
Searched information	True	False	3.10	3.82	0.000	True	0.74

Worry level	Worry level	None	1.07	1.06	0.180	True	1.19

Regularised ordinal regression sensitivity analysis for Knowledge of characteristics. *Comparison between odds ratios (ORs) from an unweighted refit of the main ordinal regression model (proportional odds, same predictors as the main analysis) and from a Ridge-penalised ordinal regression (ordinalNet, cumulative logit link, α = 0, λ =* 0.0622*) fitted on the same predictors without survey weights. Since both models are unweighted, any differences in the estimated ORs are attributable solely to the effect of regularisation. The Shrinkage column reports the ratio of the ordinalNet OR minus 1 to the unweighted polr OR minus 1, indicating how much the Ridge penalty pulls each association toward the null. Direction agreement between the two models supports the robustness of the associations reported in the main analysis.*

Categorical Variable	Variable	Reference	OR regularised (λ=0.0140)	OR unregularised	P-values unregularised	Agreement of direction	Shrinkage
Sex	Female	Male	1.22	1.22	0.006	True	0.97
Occupation	True	False	0.70	0.63	0.000	True	0.80
Urbanicity	Rural	Urban	1.17	1.18	0.046	True	0.94
Symptoms	True	False	1.44	1.46	0.094	True	0.96
Comorbidities	True	False	1.10	1.12	0.163	True	0.83
Autochthonous cases	True	False	1.52	1.55	0.000	True	0.94
Vaccination status	True	False	0.90	0.88	0.829	True	0.80
Searched information	True	False	1.95	2.02	0.000	True	0.93
Worry level	Worry level	False	1.63	1.67	0.000	True	0.94
Knowledge score	Poor	Excellent	0.63	0.61	0.000	True	0.94
Knowledge score	Medium	Excellent	0.74	0.72	0.001	True	0.91
Knowledge score	Good	Excellent	1.00	0.98	0.812	True	0.18
Efficacy score	Efficacy score	False	1.61	1.64	0.000	True	0.95

Regularised ordinal regression sensitivity analysis for Adoption of measures. *Comparison between odds ratios (ORs) from an unweighted refit of the main ordinal regression model (proportional odds, same predictors as the main analysis) and from a Ridge-penalised ordinal regression (ordinalNet, cumulative logit link, α = 0, λ =* 0.0622*) fitted on the same predictors without survey weights. Since both models are unweighted, any differences in the estimated ORs are attributable solely to the effect of regularisation. The Shrinkage column reports the ratio of the ordinalNet OR minus 1 to the unweighted polr OR minus 1, indicating how much the Ridge penalty pulls each association toward the null. Direction agreement between the two models supports the robustness of the associations reported in the main analysis.*

3.2 Effect size presentation. For key predictors, present adjusted effect sizes (odds ratios or proportional odds ratios) with 95% confidence intervals and p-values in the main tables; highlight the most policy-relevant associations in the text.

We agree with the reviewer regarding the importance of clearly presenting adjusted effect sizes and their confidence intervals. To maximize readability and allow for an immediate visual comparison of effect magnitudes and precision across multiple predictors, we chose to present all adjusted Odds Ratios (ORs) and their 95% Confidence Intervals (CIs) in the manuscript's figures rather than in main text tables. This visual approach is standard practice for regression outputs in epidemiological literature. We confirm that all relevant CIs are provided in the figures. We did not include the CIs in the text in order to ease readability, and to avoid too much redundancy. Additionally, the most policy-relevant associations are thoroughly highlighted and contextualized in the Results and Discussion sections. We have now as well added in the supplementary materials tables reproducing the data from the figures shown in the main text (Tables S9 and S10).

3.3 Handling of weights. Describe how post stratification weights were incorporated into regression models (e.g., survey weighted regression, robust standard errors) and whether variance estimation accounted for weighting.

The post-stratification weights were included in the regression model as case weights, modifying the weight provided to each observation in the estimation of the likelihood function. The variance estimations accounted for this weighting. This has now been specified in the methods section.

3.4 Multiple comparisons. Given many outcomes and predictors, discuss the approach to multiple testing or emphasize effect sizes and consistency rather than isolated p values.

We thank the reviewer for raising this important point. In our analysis, we used a backward stepwise regression to select covariates based on p-values during the model-building phase. In line with the literature, this approach aligns with our exploratory objectives and the need to identify the most parsimonious set of predictors. However, for the final model, we explicitly shifted our focus to effect sizes (e.g., adjusted odds ratios) and their 95% confidence intervals, rather than isolated p-values, to assess the magnitude and relevance of the associations. This approach allows us to prioritize the strength and direction of the relationships between predictors and outcomes, which we believe is more informative for interpreting the results in the context of our study. In the Results section, our model interpretation is based on effect sizes, not statistical significance alone. This has now been specified in the methods section.

4. Contextual and Temporal Factors

4.1 Timing of data collection. Data were collected May-October 2024; discuss how seasonal variation and contemporaneous outbreaks (e.g., the Italy 2024 outbreak) may have influenced awareness and behaviours.
We thank the reviewer for their suggestion. As indicated in the methods, longitudinal data was collected in Italy for the purpose of assessing the impact of a potential summer 2024

outbreak on knowledge, awareness, and behaviours. However, this analysis goes beyond the scope of this paper, and will be the focus of further work. Nonetheless, as the reviewer suggests, contemporaneous outbreaks may have had an impact on the responses. In France, where the majority of the responses were gathered, participants included filled in a questionnaire in a short time span, between July 29th and August 6th. It is highly unlikely that responses would have been affected by the development of the outbreak, which took place on a much slower scale. Responses obtained in Italy were instead gathered over a longer timespan, between May and October 2024. While it is possible that contemporaneous outbreaks may have slightly affected the observed levels of awareness, worriness, and knowledge, more than 85% of the responses were gathered before the end of June 2024, prior to the first local transmissions detected in July 2024 [source: https://www.epicentro.iss.it/arbovirosi/dashboard-2024]. Moreover, 93% of the participants in Italy replied to the questionnaire before the 11th of September 2024, when the biggest outbreak in 2024 was initially detected in Italy in Fanno [Sacco et al. Eurosurveillance 2024] and started to receive significant media attention. The limitations of the manuscript were updated to reflect this.

4.2 Information environment. The manuscript notes that 16% sought information; expand on sources of information (media, health authorities, social networks) and whether source type modifies knowledge or vaccine willingness.

We thank the reviewer for the comment. 14.8% of participants indeed reported having sought information. For those participants, they could select among a list of options which information sources they had sought, from the list: *“Scientific information and dissemination web sites; Foreign newspapers; National newspapers; Local newspapers; Blogs (Reddit, etc.); Social media (TikTok, Instagram, Facebook, X (Twitter), etc); Your doctor; Other (Please, specify where else you looked for information)”*. The resulting distribution of information sources consulted by participants is visually presented in Figure 1B, and the corresponding descriptive statistics are reported in the Results section (*subsection 3.1.2. - Awareness of dengue, and knowledge of its characteristics*), where we note that 69.0% utilized scientific websites and 32.8% consulted national newspapers. Participants were also able to enter information in a text box if they felt their information sources didn’t match the list. This was done by 12.55% of the participants that declared having sought information. A detailed analysis of these answers is beyond the scope of this work.

We thank the reviewer for their suggestion to evaluate whether the type of source modifies knowledge or vaccine willingness, and agree that this is a highly interesting hypothesis. However, only a small minority of the total sample (14.8%) actively sought information. As a result, stratifying this subset further by specific information sources would result in sparse data and underpowered estimates within our multivariate models. Consequently, we can not include specific source types as explanatory variables in our regression models.

5. Ethics, Data Availability, and Reproducibility

5.1 Data sharing constraints. The Data Availability Statement indicates that raw data cannot be shared due to privacy concerns. Provide a clear pathway for data access (e.g., controlled

access, data use agreements) and describe what deidentified aggregate data or code will be made available.

We thank the reviewer for raising this important point. As indicated in the submitted material, the raw data cannot be shared, as the informed consent provided by the participant doesn't allow for the data to be shared at the aggregation level required to reproduce the findings. Access to the data for reasonable scientific requests, under a formal data sharing agreement may be discussed by email with the corresponding author Daniela Paolotti.
The data availability statement has been updated to reflect this:

"*The informed consent provided by the platform users doesn't allow for the publication of the data at the aggregation level needed to reproduce the findings of the study. Data sharing requests under formal data sharing agreement may be addressed to contact@grippenet.fr and support@influweb.org for the Grippenet/Covidnet and the Influweb samples respectively."*

5.2 Ethics approval and consent. Explicitly state the ethics committee approvals, informed consent procedures, and data protection measures used for the InfluenzaNet cohorts and the dengue survey.
Ethics approval and consent is of the utmost importance, and we direct the reviewer to the *section 2.9 Ethics approval and consent to participate* in the manuscript, where the relevant details are presented. These are reproduced below for the reviewer's convenience:

"This study was conducted in agreement with country-specific regulations on privacy and data collection and treatment. It involved exclusively adult participants. Informed written consent was obtained from all participants enabling the collection, storage, and treatment of data, and their publication in anonymized, processed, and aggregated forms for scientific purposes. In addition, approvals by Ethical Review Boards or Committees were obtained, where needed according to country-specific regulations. The authors coordinated the data collection via the Grippenet/Covidnet and Influweb platforms. Data collection occurred between May 13, 2024, and October 27, 2024, in Italy and between July 29, 2024, and August 26, 2024, in France. The authors accessed the pseudonymized dataset for analysis during this same period.
In France, the Grippenet/Covidnet study was approved by the Comité consultatif sur le traitement de l'information en matière de recherche (CCTIRS, Advisory committee on information processing for research, authorization 11.565) and by the Commission Nationale de l'Informatique et des Libertés (CNIL, French Data Protection Authority, authorization DR-2012-024). This authorization included the possibility to add non-identifying questions or questionnaires, in line with the research objective (surveillance of diseases in the general population). The present study falls under the scope of this authorisation.
For influweb.it the research was conducted in accordance with Italian Data Protection Authority (Garante per la protezione dei dati personali) regulations on privacy, data collection and treatment, reported here https://www.garanteprivacy.it/web/garante-privacy-en. The Italian Data Protection Authority adheres to the norms set by the European Union's General Data Protection Regulation (GDPR). Influweb's data collection process was reviewed and approved by the institutional review board of the ISI Foundation which waived the need for ethics approval as the following applies: all use takes place in compliance with the rules contained in the GDPR; informed consent was obtained online from all participants of the platform at enrollment according to regulations, enabling the collection, storage, and treatment of data, and their publication in anonymized, processed, and aggregated forms for scientific purposes; the website has a "Privacy Statement" section in which the users who decide to enroll in the study can find all the information on who is responsible for the data

acquisition and processing in each country. Informed consent was obtained online from all participants enabling the collection, storage, and treatment of data, and their publication in anonymized, processed, and aggregated for scientific purposes. The Influweb website (https://influweb.org/) has a "Privacy Policy" section in which the users who decide to enroll in the study can find all the information on who is responsible for the data acquisition and processing. To ensure privacy and data security, all collected data is pseudonymized—participants' personal identifiers (e.g., email addresses) are stored separately and are inaccessible to researchers. Survey responses are linked only via an anonymized ID, preserving full privacy. Influweb operates on Google Cloud Platform (GCP) infrastructure in Europe, maintaining GDPR compliance, with continuous backups. Additionally, security measures to protect participant data include hashed passwords, two-factor authentication (2FA) and protection of data in transit by Transport Layer Security v1.3 ("TLSv1.3"). The additional questions that were added to the survey for the purpose of this study are fully consistent with the purposes previously communicated to the participants. Consequently, it was not deemed necessary to issue a new privacy notice and/or obtain additional consent from the participants nor obtain further ethical approval. We emphasize that responding to the new questions is also optional, and the publication of the survey results does not include personal data, but only aggregated and therefore anonymous data."

5.3 Supplementary materials. Ensure the full questionnaire (S1 Text) and code for analyses are provided as supplementary files or via a repository to enable reproducibility.

We confirm that the full questionnaire is provided in the S1 Text. As detailed for point 5.1, the data cannot be shared. To ensure reproducibility, the methods in the manuscript were described with a level of precision allowing full re-implementation of the method.

6. Interpretation and Causal Language

6.1 Avoid causal claims. The cross sectional design limits causal inference. Rephrase language that implies causality (e.g., "X led to Y") and emphasize associations and potential confounding.

We thank the reviewer for raising this important point. Indeed, we completely agree that associations identified are not causation, and we avoid making such claims. We have carefully checked the language used in order to remove any remaining causal claims from the text. To this effect, we changed "affect" with "correlate with" in the sentence *"Among Italian participants, those with an occupation were less likely to know a higher number of characteristics than those without, whereas in France employment status did not correlate with knowledge.*" In the sentence "*Our findings on the negative impact of the knowledge of a case on vaccination willingness is unexpected*." was also changed to "*Our findings on the negative association of the knowledge of a case on vaccination willingness is unexpected.*" In the sentence "*Knowledge and attitudes, socio-economic factors and the context explained the adoption of preventive measures, suggesting targeted approaches to increase adoption.*", we replaced "*explained*" with *"were associated with".*

6.2 Heterogeneity and equity. Expand the discussion of rural vs. urban findings and their implications for equity. Why might rural residents show lower knowledge but higher adoption of measures?

We thank the reviewer for raising this interesting point. The interplay between knowledge, adoption of measures and urbanicity observed in our study stems from different effects. As discussed in the manuscript, the association between rurality and adoption of preventive

measures is partially due to the nature of the preventive measures considered: while some of them are relevant in any context, removing stagnant water from gardens or avoiding swampy areas are more applicable in rural areas. Instead, while knowledge was found to be associated with higher adoption of preventive measures in all contexts, our analysis shows that rural residence was associated with lower knowledge levels. This is well documented in the literature [Aljessim et al. Patient Education and Counseling, 2020]. As a result, while participants living in urban areas may show higher health literacy than their rural counterparts, the latter may be more prone to adopt preventive measures that typically do not apply in cities. The discussion section of the manuscript has been enriched to precise these elements. The interplay between urbanicity, knowledge and adoption of preventive measures demonstrated in our study is a highly interesting effect, but future work is necessary to disentangle the different interactions at play, and derive robust implications for equity.

7. Clarity and Structure

7.1 Concise Results. Some result descriptions are dense; use summary tables and figures (e.g., forest plots) to make key associations more accessible.

We thank the reviewer for this point. We refer the reviewer to the manuscript, where all findings are supported by figures. In particular, all modelled determinants of knowledge, attitudes and preventive practices are already presented using forest plots, as suggested by the reviewer (see Figures 4 & 5). As explicitly stated in the journal guidelines, figures were submitted separately to the main text document. Additional tables reproducing the effects shown in the existing forest plots are now also provided in the Supplementary Information (Tables S9 and S10), in order to provide additional visualisations options. We report below Figures 4 & 5 which makes the key associations highly accessible.

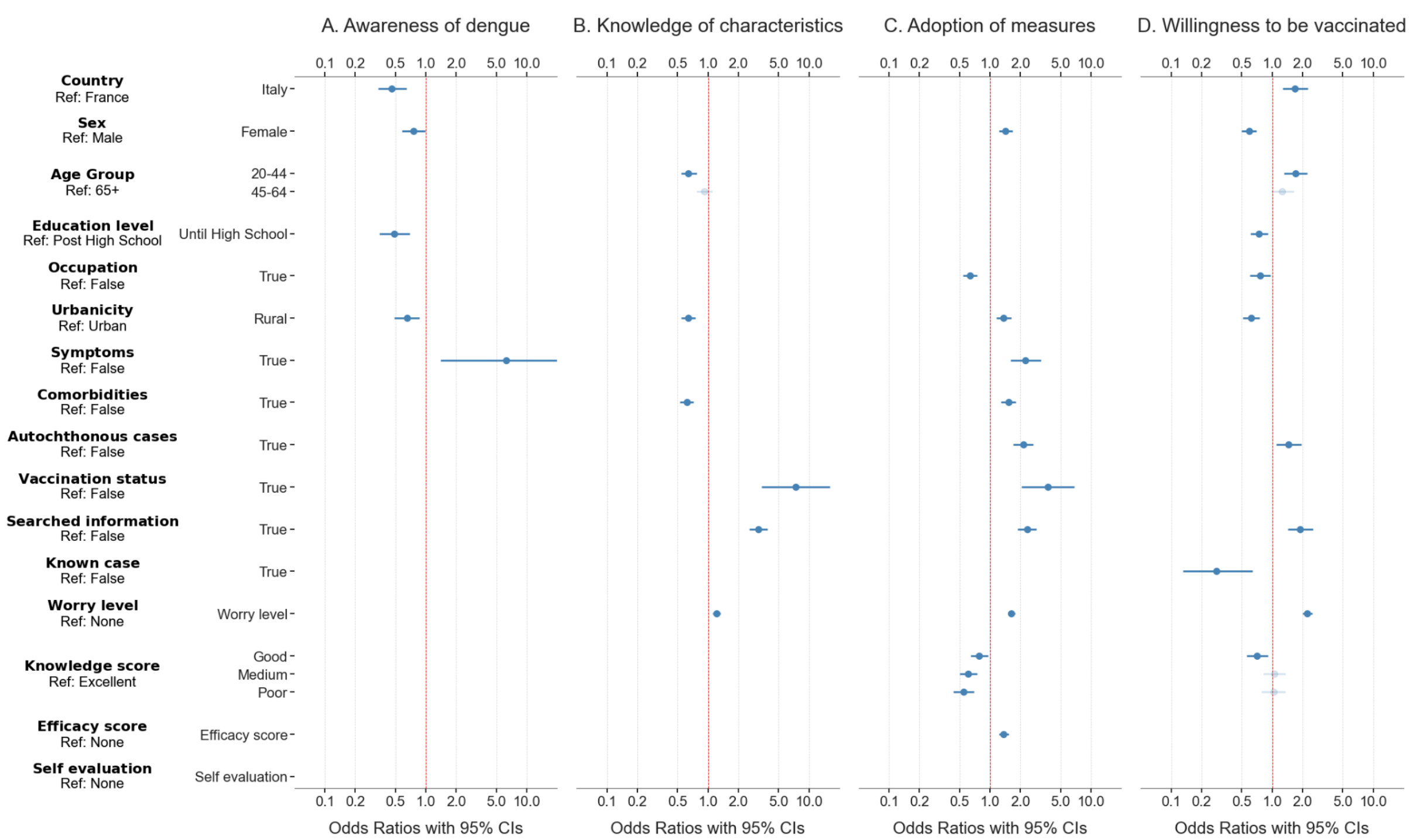

Fig 4. Determinants of knowledge of dengue, its characteristics, adoption of preventive measures, and vaccination willingness. A) *Determinants of the awareness of dengue identified with a logistic regression model in the form of odds ratios with 95% confidence intervals.* ***B)*** *Determinants of the knowledge of dengue*

characteristics, obtained with an ordinal regression model applied to the answers of the participants who have heard of dengue. ***C)*** *Determinants associated with adoption of preventive numbers (obtained from an ordinal regression model, among participants who have heard of dengue).* ***D)*** *Determinants of willingness to be vaccinated (binary logistic model) among the participants who are unvaccinated. Models are multivariate, with variables selected as described in the Methods section. Variables without an associated OR for a given outcome were either not included as explanatory variables in the initial list of considered determinants, or were excluded during the variable selection process. Faded points represent non significant levels of included variables. All analyses are conducted on the pooled dataset including French and Italian samples.*

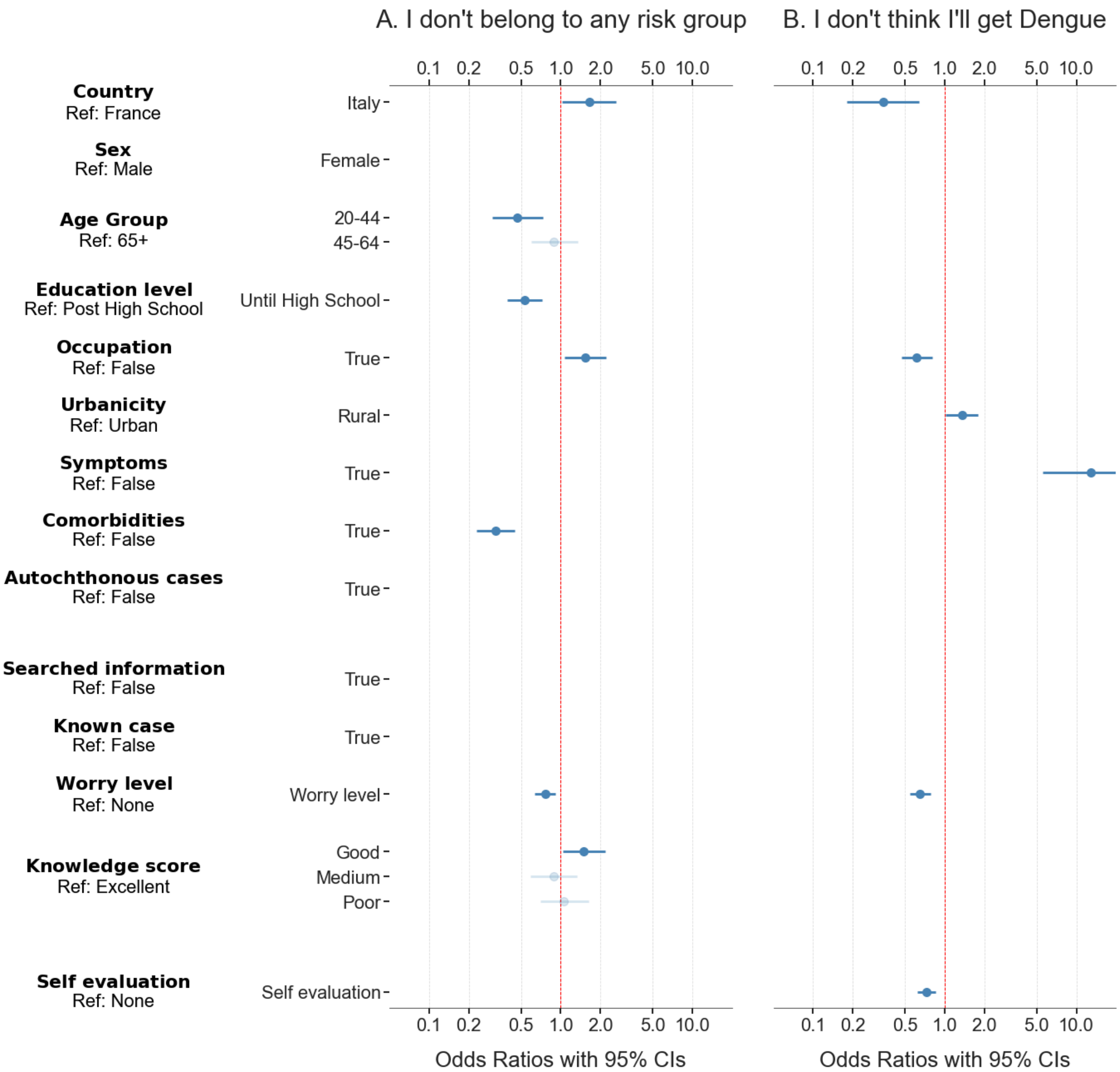

Fig 5. Drivers of the main reasons for vaccine refusal. A) *Determinants of citing "I don't belong to any risk group" in the form of odds ratios with 95% confidence intervals.* ***B)*** *Determinants of citing "I don't think I'll get dengue" in the form of odds ratios with 95% confidence intervals. Odds ratios were obtained from a multivariate, bias adjusted logistic regression model applied to the participants who are unwilling to be vaccinated. Variables without an associated OR were excluded during the variable selection process. Faded points represent non significant levels of included variables. All analyses are conducted on the pooled dataset including French and Italian samples.*

7.2 Typographical and formatting issues. Address minor formatting artifacts (line breaks, numbering) present in the draft to improve readability.

We thank the reviewer for this point, and have checked the draft again for such formatting issues.

Policy Implications

7.3 Targeted risk communication. The finding that seeking information and worry levels are strong determinants of knowledge and preventive behaviours suggests public health campaigns should prioritize accessible, locally relevant information and risk communication to stimulate appropriate concern without causing undue alarm.

We thank the reviewer for noting this point and recognising the value of our findings for informing public health campaigns. We added more precise information on how such campaigns should be tailored.

7.4 Vaccination strategy messaging. With 54.6% willingness to accept a vaccine if offered but low current uptake, policymakers should prepare clear guidance on vaccine eligibility, safety, and target groups, and address the dominant reason for refusal (low perceived risk) through tailored messaging.

We thank the reviewer for noting this point, and highlighting the policy implications of our findings regarding vaccination. As detailed previously in this document, our observation of low vaccine uptake is a direct consequence of the current dengue vaccine eligibility in France and Italy, which is itself a reflection of the low circulation levels of dengue in European countries. Our study suggests that, in the future, a potential vaccine may be well received if offered, especially if accompanied by effective communications highlighting the risk of dengue infection and severe outcomes for the population. This aspect has been precised in the discussion.

7.5 Rural outreach. The observed pattern of lower knowledge and vaccine willingness but higher preventive measure adoption in rural areas indicates that interventions should be context-sensitive: leverage existing preventive behaviours while improving factual knowledge and vaccine confidence.

We thank the reviewer for noting this point, and for recognising the importance of focusing on the local context (especially rural areas) when designing campaigns to raise knowledge and encourage vaccination uptake, in the case of a hypothetical future roll out, and change in eligibility. We have added elements in the discussion in order to highlight the need for designing context dependent interventions, and the implications for rural areas.

7.6 Surveillance and preparedness. Participatory platforms like InfluenzaNet can be integrated into early warning and community engagement strategies for Aedes borne disease preparedness, but authorities should account for representativeness limitations when using such data for policy decisions.

We thank the reviewer for noting this important point, and recognising the relevance of participatory platforms for early warning and community engagement related to mosquito borne diseases. We have now included this aspect in the discussion.

Overall Recommendations for Revision
Major revisions required before acceptance

1. Strengthen sampling and representativeness reporting.

1.1 Provide invitation and response rates, compare respondents vs. non-respondents, and expand the discussion of residual selection bias after weighting.
Done, see point by point reply above.

1.2 Justify pooling of countries and present sensitivity or stratified analyses if heterogeneity exists.
Done, see point by point reply above.

2. Enhance measurement validity and transparency.
2.1 Report psychometric properties of composite scores, handling of missing items, and rationale for symptom definitions.
Done, see point by point reply above

2.2 Clarify how vaccination status was ascertained and consider sensitivity checks.
Done, see point by point reply above.

3. Clarify statistical methods and present fuller results.
3.1 Describe model selection criteria, how weights were used in regression, and present full adjusted estimates with confidence intervals in the main tables.
Done, see point by point reply above.

3.2 Address multiple testing concerns or contextualize findings accordingly.
Done, see point by point reply above.

4. Improve reproducibility and ethics reporting.
4.1 Provide ethics approval details, consent procedures, and a clear, practical data access statement (controlled access or aggregated datasets and analysis code).
Done, see point by point reply above.

4.2 Make the questionnaire and analysis code available as supplementary materials or via a repository.
Done, see point by point reply above.

5. Refine interpretation and discussion.
5.1 Avoid causal language; discuss alternative explanations and potential confounding.
Done, see point by point reply above.

5.2 Expand on implications for targeted communication, rural outreach, and integration with public health surveillance.
Done, see point by point reply above.

Minor revisions
• Clean up formatting and typographical issues.
Done, see point by point reply above.

• Add concise visual summaries (figures) for main associations.
Done, see point by point reply above.

• Expand the brief discussion on the role of information sources and seasonal/contextual influences.
Done, see point by point reply above.

Conclusive Impression
The manuscript addresses an important and timely public health issue and has strong potential to inform preparedness and communication strategies in Europe. However, major revisions are necessary to improve transparency on sampling and weighting, strengthen measurement validity, clarify statistical methods and reporting, and ensure reproducibility and ethical clarity. After these revisions and a clearer presentation of the adjusted effect estimates, the manuscript would be suitable for publication in a public health journal focused on infectious disease preparedness and health communication.

Reviewer #2:

Review Summary: The manuscript "Assessing dengue knowledge, attitudes and preventive practices: a multicountry digital cohorts study" addresses an important public health issue, given the rising presence of Aedes mosquitoes and recent dengue outbreaks in Europe. The study leverages participatory surveillance cohorts in France and Italy to explore knowledge, attitudes, preventive practices, and vaccination willingness. While the topic is timely and relevant, several aspects of the title, objectives, methodology, and sampling warrant closer attention.

We thank the reviewer for the time they dedicated to the evaluation of our manuscript, and for raising several points that needed improving. We have taken the reviewer's comments into careful consideration in order to improve our manuscript, in particular by modifying the title, stating study aims more clearly, and expanding the limitations of the manuscript.

Title: The current title is misleading. Although the data were collected from digital surveillance cohorts, the analysis is cross-sectional rather than longitudinal. Referring to it as a "cohort study" may confuse readers. A more accurate formulation would be "cross-sectional survey using digital cohorts" or "participatory surveillance-based survey in France and Italy."

We thank the reviewer for their constructive suggestion. We have now changed the title to *"Assessing dengue knowledge, attitudes and preventive practices in France and Italy: a cross-sectional study using participatory surveillance cohorts"*

Objectives and Rationale: The objectives are present but not sharply defined. It is unclear whether the study aims primarily to describe KAP levels, compare France and Italy, or identify determinants for policy guidance. The rationale, while relevant, is only moderately

strong: it cites climate change and outbreaks but does not fully articulate why this survey is uniquely necessary now or how findings will directly inform interventions. Both objectives and rationale would benefit from clearer focus and stronger justification.

We thank the reviewer for their constructive feedback. The aims of our study were two-fold: in a first step, describe current KAP levels following recent autochthonous outbreaks in mainland Europe and in a second step, identify possible determinants for policy guidance. The study was moreover designed to include two countries in order for the analysis to capture different social contexts. The rationale behind the study was as follows: while a few studies have investigated some of these aspects (primarily in France), their findings are now outdated. Indeed, local outbreaks of dengue have been increasingly reported in France and Italy, and we expect that these have affected the populations' KAP. Moreover, since the threat posed by dengue is expected to increase, it is of timely importance to understand KAP levels in these newly exposed populations, and derive determinants for policy guidance. Given that dengue prevention largely relies on the adoption of preventive measures against mosquito bites and proliferation, engaging the local population is crucial in controlling future outbreaks. Identifying these determinants can therefore inform the designs of campaigns aimed at increasing preventive measures adoption in order to curb the local spread of dengue. We have now modified the introduction to present our aims and rationale more clearly. Implications of our findings for intervention designs are reported in the discussion.

Methodology: The use of InfluenzaNet is innovative and cost-effective, and the statistical methods (logistic and ordinal regression) are appropriate. Post-stratification by age, sex, and education is a reasonable attempt to correct representativeness bias. However, the methodology has limitations: The design is cross-sectional, not cohort-based. It no where mentioned about nature of study in the article. Pooling France and Italy may obscure country-specific differences. Multicountry word is overrated. Reliance on self-reported data introduces recall and social desirability bias. The definition of "symptoms" is restrictive and may under-detect possible dengue-like illness. These limitations should be acknowledged more explicitly.

We thank the reviewer for their feedback. Indeed, the design of the survey as analysed is cross-sectional. However, there are two relevant points to note: (i) the study leverages existing participatory longitudinal cohorts, by sampling participants from these cohorts; and (ii) in the case of the Italian sample, the study design was inherently longitudinal, and participants filled in the same survey several times over the study period. For the present analysis, a cross-sectional subset of the Italian sample was considered by considering only the first reply of each participant, which allowed the evaluation of KAPs unaffected by previous exposure to the questionnaire. In order to clarify this aspect in the manuscript, we have now specified the study type being "cross-sectional" wherever relevant, including in the title, abstract and methods and conclusions.

We agree with the reviewer that pooling both samples may obscure differences, should there be some. For this purpose, we have conducted an analysis of the homogeneity of the two samples, and have reported in the results the key output of this analysis, with details left to the Supplementary Materials. In particular, these findings showed that *"In the majority of the determinants considered, interaction terms with the country of residence were not significant, indicating that the determinant's effects apply independently of countries."* Still, some differences between countries were observed, and these were detailed in the results section

of the manuscript. We have now additionally added a sentence in the limitations to specify that pooling the two samples may indeed obscure differences beyond what we observe.

In the new title of the manuscript, the “multi-country” term does not appear anymore. This term did not appear anywhere in the text.

The reviewer is right that self-reported data may be affected by recall bias, which may affect the findings. We have now included elements pertaining to this in the limitations.

Regarding the definition of symptoms, our rationale for this specific definition was driven by the need to maximize epidemiological specificity, in order to assess the impact of dengue-specific symptom history on KAP. Our aim was not to detect possible cases, as this would be highly difficult in a non-endemic setting like mainland France and Italy via participatory systems. Our aim was to account for the effect of dengue-specific symptoms on participants’ behavior and information seeking, which might eventually alter their adoption of preventive practices. Whereas utilizing broad, non-specific symptoms (e.g., fever, headache, cough) would result in a high rate of false positives due to the prevalence of other circulating respiratory and gastrointestinal viruses. Therefore, we restricted our definition of symptom history to symptoms that are highly linked to dengue fever (such as retro-orbital eye pain and severe bone pain) and signs of severe dengue (hemorrhagic symptoms). This resulted in a multiple choice list of five symptoms (*eye pain, blood in the feces, blood in the vomit, bloody gums, and bone pain*). The binary variable Symptoms was then defined by requiring the presence of at least two of these five symptoms to further avoid misclassification from isolated symptoms. For sensitivity, we considered alternative definitions based on the presence of one or more symptoms, and three or more symptoms. Using these alternative variable definitions instead for our regression analyses, the statistical significance of the variable symptoms changes in some of the outcomes: in particular, in our main analysis, the variable symptoms (based on >=2 symptoms) is significant for the outcome “Awareness of dengue”, “Adoption of measures” and “I don’t think I will get dengue” as vaccine refusal reason. With the definition based on >=1 symptoms, the variable becomes non-significant in the model for “Awareness of dengue” and “Adoption of measures”, but becomes significant for the outcome “Knowledge of dengue characteristics”. When using the definition based on >=3 symptoms, symptom history becomes non-significant in all instances. The remainder of the findings are robust to these changes, with the exception of a few changes in statistical significance for determinants of the outcome “I don’t think I’ll get Dengue” which are close to the significance threshold.

We have now explicitly clarified our rationale for the definition of the symptoms variable in the Methods section. We moreover reported the exact prevalence of each symptom, as well as the prevalence of one or more, two or more, and three or more symptoms in the Results section. We included the alternative regression models obtained with the variable symptoms defined from at least one symptom in the Supplementary Materials. In the results section, we have also now detailed the sensitivity of the results on the changes in symptom history definition. Finally, we have expanded the limitations of the manuscript to highlight these aspects.

Sampling Frame and Sample Size: The sampling frame (volunteer participants in InfluenzaNet) is a convenience sample, not population-based. While post-stratification improves representativeness, it cannot fully correct for volunteer bias. The French sample (~2,500) is adequate, but the Italian sample (~404) is relatively small, limiting statistical power. Also population bellow 20 is absent in the study, though they are not immune from dengue. Overall, the sample size is sufficient for exploratory cross-sectional analysis, but generalizability remains limited.

We thank the reviewer for their comment, and we agree with the reviewer's assessment. Indeed, the sample does not include any participants below 18 years old for ethical reasons. The few participants between 18 and 20 years old were excluded to align with census data on age-sex and education level. As pointed out by the reviewer, this is a limitation of the sample, which we have now included in the limitations.

We agree with the reviewer on the difficulty to generalise findings to the general population. Where possible, we have edited the manuscript to remove unintentional generalising claims; and have added details in the limitations on this.

Overall Assessment: This study provides useful exploratory insights into dengue-related knowledge, attitudes, and practices in non-endemic European contexts. However, the title should be revised to avoid methodological misclassification, and the objectives and rationale need sharpening to clarify the study's purpose. The methodology is not sound, constrained by sampling limitations and biases inherent to participatory surveillance. It must be reconsidered. With these refinements, the manuscript could make a valuable contribution to preparedness strategies for emerging mosquito-borne threats in Europe.

We thank the reviewer for raising all these points which have now improved the readability and contextualization of our work. We hope that our manuscript can now be considered suitable for publication.

Reviewer #3:

Lucille Calmon et al., in their manuscript intitled "Assessing dengue knowledge, attitudes and preventive practices: a multicountry digital cohorts study", leveraged French and Italian participatory surveillance cohorts from the European network InfluenzaNet to investigate emerging threats unrelated to respiratory viruses. They collected data on knowledge, attitudes, and preventive practices related to dengue in France and Italy, where dengue is an emerging threat. Their findings revealed high awareness, moderate levels of concern, and moderate willingness to be vaccinated, highlighting the urgent need for communication campaigns to strengthen knowledge and awareness of these lesser-known aspects.

This study is dense, highly relevant, and of significant public health importance, as it allowed the authors to analyze participants' knowledge, attitudes, behaviors, and practices related to dengue in a non-endemic context such as Europe.

I believe this study is suitable for consideration by PLOS Global Public Health for publication.

The manuscript is well written and easy to read. However, I have a few comments that could help the authors further improve this important work:

We thank the reviewer for the time they dedicated to the evaluation of our manuscript, and for their positive feedback. We are glad that the reviewer found our study of interest, and high relevance. We are particularly grateful for the reviewer' many comments which allowed us to improve the readability of the draft in several locations.

Specific Comments

Line 21: Please add "worldwide" at the end of the sentence.

Done.

Lines 66–67: Add "the" before assessment in the sentence "In 2025, guidance from the ECDC on assessment…" and delete the second "of Aedes-borne".

Done.

Line 68: Replace "faces high numbers of importations of dengue cases in" with "reports large numbers of imported dengue cases into".

Done.

Line 71: Replace "dengue importations" with "imported dengue cases" and "came from" with "originated from".

Done.

Line 72: Elaborate further (why) on the statement "the dengue vaccine Qdenga was approved in Europe in 2022, but is not recommended in mainland France."

We thank the reviewer for their comment. The vaccine is generally not recommended to individuals who have not been previously infected, as this may put them at a higher risk of developing a severe form of dengue if they were to become infected. In mainland France, given the currently low prevalence of previous infection with dengue, and the low risk of becoming infected, authorities do not recommend the vaccine. However, in 2025, the recommendations were updated to allow vaccination of travellers on a case-by-case basis, similar to what is done in Italy. This is an important point, which has now been clarified in the introduction.

Line 81: Replace "benefits gained from the" with "perceived benefits of".

Done.

Lines 93–96: Specify the country/countries where these observations were made.

Done.

Lines 107–111: The sentence is too long. Consider splitting it into two and replacing "but" with "However". Also revise as follows:

- “in a sample randomly selected in colonised departments” to “in a randomly selected sample from colonized departments”

- “found an increasing awareness of the mode of transmission of dengue” to “revealed increasing awareness of dengue transmission”

- “knowledge of other mosquito-borne viruses characteristics” to “knowledge of the characteristics of other mosquito-borne viruses”

We thank the reviewer for their detailed suggestions, the above suggestions for lines 107-111 have been implemented.

Line 159: Replace:

- “between” by “from”

Done.

- “The data was” by “Data were”

Done.

- “collected to capture” by “intended to capture”

Done.

- “responses filled out until” by “responses submitted until”

Done.

Statistical analysis:

- Rephrase “ordinality of the different levels of knowledge and measure adoption” as “the ordered nature of knowledge levels and preventive measure adoption”.

Done.

- Avoid repeating “analyses were conducted”.

Done, the second instance was replaced with *“The statistical analysis was implemented”.*

- Change “called in python with r2py” to “accessed in Python via rpy2”.

Done.

Explanatory variables:

- Avoid repetition of participants’ intake survey.

Done.

- Change “education attainment level” to “educational attainment”.

Done.

- Change “the presence of at least one comorbidity” to “presence of at least one comorbidity”.

Done.

Line 277: Clarify what other commonly reported reasons for refusing vaccination were. It is known that in France, “historical mistrust” and “fear of side effects” are among the leading reasons of refusing vaccination…

We thank the reviewer for pointing this out. Participants were offered a selection of possible reasons for vaccination refusal, in line with known commonly cited reasons. The complete list was as follows, including mistrust and fears of side effects: *“I don't belong to any risk group; It is better to build your immune defences naturally; I have doubts about the effectiveness of the vaccine; I don't think I'll get dengue; I am concerned that the vaccine is unsafe or may cause illness or side effects; I don't like receiving vaccinations; The vaccine is currently too expensive; My doctor advised me against getting vaccinated; None of the above (Please, specify the reason)”*. We have now updated the Methods section to explicitly list all the options provided in the survey, which indeed included concerns about vaccine safety, side effects, and effectiveness. The adjusted proportion of participants selecting each reason was already shown in our Results section (Figure 3) for all participants. In particular, while mistrust and fear of side effects are known to be commonly reported in France, in our specific study, “*I am concerned that the vaccine is unsafe or may cause illness or side effects*” and “*I don't like receiving vaccinations*” were reported as refusal reason by only 16.9% and 12.5% of the French respondents (after adjustment) unwilling to be vaccinated; risk perception was instead a more common reason cited, with 39.5% of the French participants reporting the reason “*I don't think I'll get dengue*”, and 36.2% reporting *“I don't belong to any risk group”.*

In order to maintain statistical power in our regression models, we restricted our modeling to the two most prevalent answers (>35%). Beyond the few proposed options, participants were also able to enter other reasons in a text box. This was done by 8.20 % of participants who would refuse a vaccine, and a manual evaluation of these answers is consistent with the overview obtained by the offered categories. The vaccination reasons for refusal are not a focus of the study, a more consistent analysis of these answers would be needed to characterize vaccine refusal but this goes beyond the scope of this study.

Ethics approval and consent to participate:

- “In France, the Grippenet/Covidnet study was approved, and in Italy, the influweb.it study was conducted in accordance with the Italian Data Protection Authority. However, as these approvals were initially not intended for research on dengue or other diseases, it is expected that an approved addendum should be provided by the authors, stipulating that the authorized cohorts may also be used for additional studies.”

In France, the CNIL authorization obtained during the implementation of Grippenet/Covidnet included the option to add non-identifying questions or questionnaires, in line with the research objective (surveillance of diseases in the general population). The present study falls under the scope of this authorisation. A copy of the authorisation is available in the

ethics documentation provided to the journal (authorisation request n 911510V1, decision n DR-2012-204 on the 20/04/2012). The additional questions that were added to the survey are fully consistent with the purposes previously communicated to the participants in Italy. Consequently, it was not deemed necessary to issue a new privacy notice and/or obtain additional consent from the participants nor obtain further ethical approval. We emphasize that responding to the new questions is also optional, and the publication of the survey results does not include personal data, but only aggregated and therefore anonymous data.

The ethics statement has been updated to precise these points, and we are now additionally providing the authorisation request n 911510V1, for which we had provided the approval DR-2012-204 previously.

Results:

- Change “while the French sample was biased towards females” to “whereas the French sample was skewed toward females”.

We thank the reviewer for their suggestions.

Fear and vaccination:

- It would be useful to know the other most frequently cited reasons for refusing vaccination if offered.

We refer the reviewer to our Results section (Figure 3), where we show the adjusted proportions for each possible reason, and the text of the section *“ 3.1.4 Fears and vaccination”* where this is discussed. As highlighted in response to the reviewers’ previous comment, the analysis of vaccination reasons for refusal not being a focus of the study, a rigorous analysis of the minority of answers provided in a free-text box is beyond the scope of this study.

Discussion (Lines 699–701):

- I do not agree with the authors’ assumption that future vaccines against dengue would be well received in France and Italy based the refusal reasons I cited above. Si please elaborate further on this.

We thank the reviewer for their question. Our results show that more than 54% of the participants (after post-stratification) would be willing to receive a vaccine, if offered. On this basis, a vaccine is considered positively by a large fraction of the sample. This is a surprising observation given the moderate concern levels observed in the samples. These findings must however be taken with caution, in light of the inherent bias of the sample. As highlighted in the manuscript, the vaccination willingness and uptake in participatory surveillance cohorts is known to be higher than in the general population. We have now replaced the statement *“However, our analysis suggests that future vaccines against dengue would be well received in France and Italy.”* with *“Still, our results suggest that, in the future, a potential vaccine may be well received if offered, especially if accompanied by effective communications highlighting the risk of dengue infection and severe outcomes for the population.”*
