# Supplementary material for: Assessing dengue knowledge, attitudes and preventive practices in France and Italy: A cross-sectional study using participatory surveillance cohorts
Source: PLOS Glob Public Health. 2026 Sep 23;6(9):e0007162. doi: 10.1371/journal.pgph.0007162 (PMC13600492; doi:10.1371/journal.pgph.0007162)
Supplement: Supplementary file 5 [file pgph.0007162.s008.pdf]

Reviewer #1:

The revised manuscript has made significant progress in clarifying methodology and enhancing transparency. Major improvements include clearer reporting of post-stratification and weighting, explicit definitions of explanatory variables, more transparent regression methods, and a more cautious interpretation of associations. The ethics and data availability statements are also improved, with controlled access pathways now described. However, several important gaps remain. Recruitment and response rates are not reported, which limits the assessment of selection bias. Psychometric validation of composite scores and handling of missing data are not addressed. Vaccination status ascertainment remains unclear, and model selection criteria are not specified. Adjusted effect sizes are not highlighted in the main text, and ethics committee approvals are not explicitly mentioned. Finally, contextual factors (such as seasonality and information sources) and equity implications (urban versus rural differences) require deeper discussion.

While the manuscript is substantially stronger, further revisions are necessary to fully meet the standards of transparency, validity, reproducibility, and clarity. Addressing these remaining issues will make the study suitable for publication and maximize its policy relevance.

We thank the reviewer for the time they dedicated to the evaluation of our manuscript. We address carefully the remaining points raised by the reviewer below, and in the resubmitted version of the manuscript.

Recruitment and response rates are not reported, which limits the assessment of selection bias.

We would like to mention that recruitment and response rates were reported in the resubmitted version in the manuscript, lines 424 - 428, (of the hereby resubmitted version) and in the response to the reviewer. As indicated in the results, *“All users registered in the Grippenet/Covidnet and InfluWeb platforms at the time of the study could fill the survey (respectively 6252 and 994 users in Grippenet/Covidnet and InfluWeb). Of the users active in the year prior to the study (4856 and 585 users in each platform), a total of 2719 and 436 users responded to the survey in France and Italy respectively, with a response rate of 56% and 69% (based on active users in the year prior to the study).”* Details on modality of recruitment to the platform and invitation are also provided in the methods. *“Recruitment to the platforms Grippenet/Covidnet and InfluWeb are driven by several processes including word of mouth, communication campaigns, newspaper and radio interviews as well as social-media promotion.”* (lines 170-172) and “*The invitation process depended on the platforms. In Italy, all registered participants to the platforms were invited by email to complete the questionnaire monthly from May 13, 2024, to October 27, 2024. [...] In France, the questionnaire was available to all registered users from July 29, 2024, and registered participants to the platform's newsletter were invited to participate by email once, on the 29th of July 2024.*” (lines 186-194).

Psychometric validation of composite scores and handling of missing data are not addressed.

We would like to point the reviewer to lines 286-288 of the manuscript where psychometric validation of composite scores were reported: “*Internal consistency for the reflective*

composite scales was assessed using weighted Cronbach's alpha, yielding coefficients of 0.915 for the Worry level and 0.814 for the Efficacy score. Although the Knowledge score was designed as a formative cumulative index, we also evaluated its internal consistency, which yielded an acceptable weighted alpha of 0.773. "

All questions were mandatory, with some questions only asked to a relevant subset of the participants. The resulting variables were only analysed for the subset of the participants to whom the questions were asked, such that no missing data was handled. This is now indicated in the manuscript, lines 179-183 *"All questions were mandatory, with some questions only asked to a relevant subset of the participants (i.e. knowledge of dengue characteristics were only asked to the participants who declared having heard of dengue before). The resulting variables were only analysed for the subset of the participants to whom the questions were asked, such that no missing data was handled."* Moreover, additional text was also added lines 290-293 to precise this point in the context of the definition of the composite variables: *"These composite variables were only defined for participants who had heard of dengue. All questions used to build the composite scores were mandatory for these participants. These variables entered the analysis as outcome variables and/or explanatory variables in regression models restricted to the sample of participants who had heard of dengue."*

Vaccination status ascertainment remains unclear,

Vaccination status was ascertained with a dedicated question in the questionnaire "*Did you get the vaccine against Dengue?*". In the resubmitted manuscript, this has been clarified with the following text *"The Vaccination status was ascertained through a dedicated question in the survey "Did you get the vaccine against Dengue?". As the dengue vaccine is highly specific and non-routine in France and Italy, individuals who received the vaccine would have received it in a highly specific context, and are likely to recall it accurately."* (lines 260-264).

and model selection criteria are not specified.

We would like to point the reviewer to the dedicated section of the manuscript where criteria for model selection were described (section *2.7. Variable selection*). As described there "*Each variable was initially tested in a univariate analysis, and those with a p-value below 0.2 were retained for the multivariate analysis. The least significant variable (highest p-value) was iteratively removed until all remaining predictors had a p-value ≤ 0.05. For categorical variables, the selection was based on the smallest p-value among its levels. This approach, based on p-values, allows us to identify the most parsimonious set of predictors. In the final models, the strength and direction of the relationships between predictors and outcomes are instead assessed, and our model interpretations are based on both statistical significance and effect sizes/directionality."* (lines 343-350).

Adjusted effect sizes are not highlighted in the main text,

We refer the reviewer to our results section (lines 527-667), where we include in the text adjusted effect sizes in the form of adjusted odds ratios for all selected variables and all outcomes, and where we include in the figures adjusted odds ratios and confidence intervals for all outcomes considered. A sentence has now been added to highlight that the effect sizes presented are indeed adjusted: *"We consider adjusted odds ratios (ORs) obtained using multivariate logistic or ordinal regression models fitted to the joint samples, with*

post-stratification (case weights) adjusting for age, sex and education distributions." (lines 526-529).

and ethics committee approvals are not explicitly mentioned.

We would like to point the reviewer to section *2.9 Ethics Approval and Consent to Participate* of our manuscript which describes the ethics approval granted for this study (lines 369-415). The corresponding text is reproduced below, where we also indicate in bold face the relevant sections explicitly mentioning the ethics committee approvals. "*This study was conducted in agreement with country-specific regulations on privacy and data collection and treatment. It involved exclusively adult participants. Informed written consent was obtained from all participants enabling the collection, storage, and treatment of data, and their publication in anonymized, processed, and aggregated forms for scientific purposes.* ***In addition, approvals by Ethical Review Boards or Committees were obtained, where needed according to country-specific regulations.*** *The authors coordinated the data collection via the Grippenet/Covidnet and Influweb platforms. Data collection occurred between May 13, 2024, and October 27, 2024, in Italy and between July 29, 2024, and August 26, 2024, in France. The authors accessed the pseudonymized dataset for analysis during this same period.*

In France, the Grippenet/Covidnet study was approved by the Comité consultatif sur le traitement de l'information en matière de recherche (CCTIRS, Advisory committee on information processing for research, authorization 11.565) and by the Commission Nationale de l'Informatique et des Libertés (CNIL, French Data Protection Authority, authorization DR-2012-024). This authorization included the possibility to add non-identifying questions or questionnaires, in line with the research objective (surveillance of diseases in the general population). The present study falls under the scope of this authorisation.

For influweb.it the research was conducted in accordance with Italian Data Protection Authority (Garante per la protezione dei dati personali) regulations on privacy, data collection and treatment, reported here https://www.garanteprivacy.it/web/garante-privacy-en. The Italian Data Protection Authority adheres to the norms set by the European Union's General Data Protection Regulation (GDPR). ***Influweb's data collection process was reviewed and approved by the institutional review board of the ISI Foundation which waived the need for ethics approval as the following applies: all use takes place in compliance with the rules contained in the GDPR; informed consent was obtained online from all participants of the platform at enrollment according to regulations, enabling the collection, storage, and treatment of data, and their publication in anonymized, processed, and aggregated forms for scientific purposes; the website has a "Privacy Statement" section in which the users who decide to enroll in the study can find all the information on who is responsible for the data acquisition and processing in each country. Informed consent was obtained online from all participants enabling the collection, storage, and treatment of data, and their publication in anonymized, processed, and aggregated for scientific purposes.*** *The Influweb website (https://influweb.org/) has a "Privacy Policy" section in which the users who decide to enroll in the study can find all the information on who is responsible for the data acquisition and processing. To ensure privacy and data security, all collected data is pseudonymized—participants' personal identifiers (e.g., email addresses) are stored separately and are inaccessible to researchers. Survey responses are linked only via an*

anonymized ID, preserving full privacy. Influweb operates on Google Cloud Platform (GCP) infrastructure in Europe, maintaining GDPR compliance, with continuous backups. Additionally, security measures to protect participant data include hashed passwords, two-factor authentication (2FA) and protection of data in transit by Transport Layer Security v1.3 ("TLSv1.3"). The additional questions that were added to the survey for the purpose of this study are fully consistent with the purposes previously communicated to the participants. Consequently, it was not deemed necessary to issue a new privacy notice and/or obtain additional consent from the participants nor obtain further ethical approval. We emphasize that responding to the new questions is also optional, and the publication of the survey results does not include personal data, but only aggregated and therefore anonymous data."

We also refer the reviewer to the documentation provided with the manuscript ("Ethics approval documentation") where the relevant approval documentation is provided.

Finally, contextual factors (such as seasonality and information sources) and equity implications (urban versus rural differences) require deeper discussion.

The discussion section in our resubmitted manuscript has been expanded to discuss these aspects more deeply. In detail:

Role of seasonality:

This is an interesting aspect and we would like to thank the reviewer for pointing this out. As previously discussed in the limitations, it is unlikely that contemporaneous outbreaks affect the answers collected: *"Autochthonous dengue outbreaks occurred in France and Italy during the data collection, and it is possible that these have impacted the levels of knowledge, concerns and adoption of preventive measures reported by participants replying later to the questionnaire. However, more than 85% of the responses considered in the Italian sample were gathered prior to the detection of the first autochthonous outbreaks in Italy in 2024. Moreover, the French sample was collected over a timespan of one week between July 29th and August 6th 2024. It is therefore highly unlikely that the results of this study were significantly affected by the development of autochthonous outbreaks concurrent to the data collection."*

Similarly, seasonal variations and their impact on dengue knowledge, attitudes and preventive measures is an interesting question. In the present study, the majority of the responses were gathered over a timespan shorter than seasonality variations. Seasonal variations during the data collection therefore do not have a measurable impact on our results. However, it is true that results obtained in the summer may be different from those obtained at other timepoints in the year. Further research focussed on this aspect would be needed to elucidate this, with a longitudinal data collection designed primarily for this purpose. These aspects have been included to the manuscript as follows:

- We have revised the resubmitted manuscript to include in the results (lines 427-431) additional details pertaining to when answers were collected: *"In France, included participants filled in the questionnaire between July 29th and August 6th; with a majority (57%) filled in the first 4 days of data collection (until August 1st). Responses obtained in Italy were instead gathered over a longer timespan, between May and*

October 2024. The majority of the responses included (85%) were received before the end of June 2024."

- The role of seasonality when comparing adoption of measures from different surveys has been highlighted, together with the need for additional research to reveal the impact of seasonality and local outbreaks on adoption of preventive measures and concern levels. In particular, we added the text: *"This increasing trend may also partly result from seasonal effects: our survey was conducted during the peak of vector activity in Europe, similarly to(37), while results presented in(40) were obtained from answers collected over half a year, and the survey from(38) was conducted during winter months. Our sample was collected over a short time span, compared to outbreak development and seasonal variations, during peak mosquito activity. This may have heightened participants' perceived risk and motivated preventive actions, particularly for measures like repellent use or eliminating standing water. Future research and dedicated data collection are needed to explicitly reveal the impact of seasonality and concurrent outbreaks on the adoption of preventive behaviors." (*lines 764-772) and the text *"Further research leveraging these platforms could moreover investigate the evolution of knowledge, attitudes and practices regarding dengue, as well as other vector borne diseases over longer timespans. This would provide necessary data to reveal the impact of concurrent outbreaks on knowledge, attitude and behaviours, as well as the role of concurrent outbreaks in shaping concern levels and preventive behaviours."* (lines 900-904).
- We have added additional elements in the limitations to discuss precisely seasonality: *"Similarly, seasonal variations during the data collection are unlikely to affect the results as the majority of the answers were collected over a timespan shorter than typical seasonal variations. But the timing of the data collection may have itself led to higher rates of preventive behaviours and higher concern levels than would have been observed at other timepoints during the year. Future longitudinal data collection would be needed to elucidate this aspect."* (lines 973-978).

Information source:

Our survey provides the proportion of participants who had actively searched for information, and the sources consulted by these individuals. Our statistical analysis further provided an overview of the associations of information search with knowledge, adoption of preventive measures and attitudes towards vaccination. As highlighted in the previous reply to the reviewer, only a minority of the total sample (14.8%) actively sought information. As a result, stratifying this subset further by specific information sources would result in sparse data and underpowered estimates within our statistical models. Consequently, further research and data collection is needed to clarify the role of specific source types. Still, we have now expanded the discussion of our manuscript in order to provide additional elements pertaining to this:

- The discussion of information search behaviours identified with our survey has now been expanded, with the following dedicated paragraph (lines 734-749): *"Understanding information source preferences can suggest specific communication channels that can be used to raise awareness, improve knowledge and encourage the adoption of preventive practices. Our survey identified sources used by the*

14.6% of sampled individuals actively searching for information on the circulation of dengue. Using these identified channels (scientific websites and national newspapers) provides a way to further inform those engaged individuals. However, future information campaigns would have the most impact when targeting individuals who do not look for information proactively, since information search was associated with higher knowledge, adoption of preventive behaviours, and willingness to vaccinate. In Spain and the Netherlands, 93.7% and 81.6% of participants were interested in receiving information on mosquitoes and mosquito-borne viruses(41). Preferred sources for receiving this information however varied between countries, with Spanish participants preferring health professional information sources, and Dutch participants preferring government sources and websites(41). Further research must be dedicated to identify targeted communication channels in the European context in order to ensure equitable reach of information campaigns across different sociodemographic groups (e.g. age, education level) and local contexts (e.g. country, urbanicity)."

- The discussion of the determinants of adoption of preventive measures was expanded to include elements pertaining to the role of information search with the following text: *"Individuals who had searched for information actively (most commonly from scientific websites and national newspapers) were also adopting a higher number of measures. The sample size did not allow for the investigation of the role of the specific information sources consulted in shaping measure adoption, an important aspect which should be the focus of future work. "* (lines 794-797).

Equity implications and urban rural differences:

The discussion section of our resubmitted manuscript was expanded in order to include aspects related to rural-urban differences and implications for equity in several places.

- We have now included a paragraph expanding on the role of employment and rural-urban differences in the adoption of preventive measures, with the following text (lines 798-820): *"Being employed was associated negatively with the adoption of preventive measures, which may stem from differences in time use and opportunities for measure adoptions. Future research in this direction would be needed to disentangle differences in exposure and opportunities between these populations, in order to design targeted campaigns aimed at increasing measure adoption in the most vulnerable subgroups. Urbanicity was negatively associated with the adoption of preventive measures. This is a concerning finding, as A. albopictus mosquitoes adapted to thrive in urban environments where they face fewer predators, have access to a high density of human hosts, and many man-made structures where they can breed(66,67). Future campaigns to encourage the uptake of preventive measures should therefore aim to raise knowledge, efficacy perceptions, inform on dengue severity and susceptibility, and should focus in particular on informing urban residents on the actions they can take to protect themselves against dengue. In contrast, information campaigns in rural areas — associated with higher adoption of preventive measures — would need to focus on raising awareness of dengue, improving knowledge of its characteristics, and promoting vaccination willingness, should a vaccine become available. Still, care must be taken to tailor these*

interventions to the local context, and current adoption of preventive measures. Indeed, our analysis revealed that, the association between urbanicity and measure adoption was country dependent, with rural residence instead being negatively associated with the adoption of preventive measures in Italy. Intervention campaigns should carefully account for these differences, and be tailored to the local context in order to be equitable. This may be achieved by working directly with local organisations and community groups to identify the specific needs of the targeted population and bridge gaps in measure adoption."

- In the context of raising knowledge of dengue awareness and characteristics, additional text was added to provide examples of tailored intervention campaigns: *"In particular, efforts should focus on the younger population, rural communities, and less educated groups, which were less likely to have heard of the disease, or know its characteristics. In the rural setting, where dengue literacy is lower, but preventive measures are more likely to be adopted, targeted information campaigns should leverage local organisations and communication channels to raise awareness and further enhance measure adoption. For example, interventions in local schools could help bridge this gap, by raising knowledge in younger individuals. Similarly, visual information campaigns in frequently visited locations (supermarket, health clinics, post office, public transportation hub) and audio-visual campaigns in local newspapers, radio and TV channels would help achieve an equitable reach across different groups. Moreover, the lower knowledge of characteristics among those with comorbidities suggests that information campaigns in medical establishments, often attended by this subpopulation, could be beneficial. Increasing knowledge in the population is particularly important, as higher knowledge is known to be associated with the adoption of preventive measures(32,35,41)."* (lines 717-730).

- In the discussion of information sources, we have highlighted the need to take these aspects into account when identifying optimal communication channels for an equitable reach of the population: "*Preferred sources for receiving this information however varied between countries, with Spanish participants preferring health professional information sources, and Dutch participants preferring government sources and websites(41). Further research must be dedicated to identify targeted communication channels in the European context in order to ensure equitable reach of information campaigns across different sociodemographic groups (e.g. age, education level) and local contexts (e.g. country, urbanicity)."* (lines 743-749).

Reviewer #3:

Assessing dengue knowledge, attitudes and preventive practices in France and Italy: a cross-sectional study using participatory surveillance cohorts R1, by Lucille Calmon et al.

I would like to thank the authors for their detailed and thoughtful responses to my comments and to other reviewers' comments. I appreciate the clarifications provided, particularly regarding the recommendations for the Qdenga vaccine in mainland France, the specification of refusal reasons for vaccination, and the ethical approval framework in France and Italy.

The revisions made to the abstract, introduction, methodology, and results sections have significantly improved the clarity and accuracy of the manuscript. I also acknowledge the careful rephrasing of several sentences, which has enhanced readability and scientific precision. Overall, I am satisfied with the authors' responses and the changes implemented. I agree that the manuscript has been strengthened and is now more suitable for publication in PLOS Global Public Health.

We thank the reviewer for the time they have dedicated in this second round to the evaluation of our manuscript, and for their positive judgment for publication.
