# Supplementary material for: Assessing dengue knowledge, attitudes and preventive practices in France and Italy: A cross-sectional study using participatory surveillance cohorts
Source: PLOS Glob Public Health. 2026 Sep 23;6(9):e0007162. doi: 10.1371/journal.pgph.0007162 (PMC13600492; doi:10.1371/journal.pgph.0007162)
Supplement: Supplementary file 6 [file pgph.0007162.s002.docx]

Review Summary

The manuscript “Assessing dengue knowledge, attitudes and preventive practices: a multicountry digital cohorts study” addresses an important public health issue, given the rising presence of Aedes mosquitoes and recent dengue outbreaks in Europe. The study leverages participatory surveillance cohorts in France and Italy to explore knowledge, attitudes, preventive practices, and vaccination willingness. While the topic is timely and relevant, several aspects of the title, objectives, methodology, and sampling warrant closer attention.

Title

The current title is misleading. Although the data were collected from digital surveillance cohorts, the analysis is cross-sectional rather than longitudinal. Referring to it as a “cohort study” may confuse readers. A more accurate formulation would be “cross-sectional survey using digital cohorts” or “participatory surveillance-based survey in France and Italy.”

Objectives and Rationale

The objectives are present but not sharply defined. It is unclear whether the study aims primarily to describe KAP levels, compare France and Italy, or identify determinants for policy guidance. The rationale, while relevant, is only moderately strong: it cites climate change and outbreaks but does not fully articulate why this survey is uniquely necessary now or how findings will directly inform interventions. Both objectives and rationale would benefit from clearer focus and stronger justification.

Methodology

The use of InfluenzaNet is innovative and cost-effective, and the statistical methods (logistic and ordinal regression) are appropriate. Post-stratification by age, sex, and education is a reasonable attempt to correct representativeness bias. However, the methodology has limitations:

- The design is cross-sectional, not cohort-based. It no where mentioned about nature of study in the article.
- Pooling France and Italy may obscure country-specific differences. Multicountry word is overrated.
- Reliance on self-reported data introduces recall and social desirability bias.
- The definition of “symptoms” is restrictive and may under-detect possible dengue-like illness.

These limitations should be acknowledged more explicitly.

Sampling Frame and Sample Size

The sampling frame (volunteer participants in InfluenzaNet) is a convenience sample, not population-based. While post-stratification improves representativeness, it cannot fully correct for volunteer bias. The French sample (~2,500) is adequate, but the Italian sample (~404) is relatively small, limiting statistical power. Also population bellow 20 is absent in the study, though they are not immune from dengue. Overall, the sample size is sufficient for exploratory cross-sectional analysis, but generalizability remains limited.

Overall Assessment

This study provides useful exploratory insights into dengue-related knowledge, attitudes, and practices in non-endemic European contexts. However, the title should be revised to avoid methodological misclassification, and the objectives and rationale need sharpening to clarify the study’s purpose. The methodology is not sound, constrained by sampling limitations and biases inherent to participatory surveillance. It must be reconsidered. With these refinements, the manuscript could make a valuable contribution to preparedness strategies for emerging mosquito-borne threats in Europe.
