# Supplementary material for: Assessing dengue knowledge, attitudes and preventive practices in France and Italy: A cross-sectional study using participatory surveillance cohorts
Source: PLOS Glob Public Health. 2026 Sep 23;6(9):e0007162. doi: 10.1371/journal.pgph.0007162 (PMC13600492; doi:10.1371/journal.pgph.0007162)
Supplement: Supplementary file 7 [file pgph.0007162.s006.docx]

The revised manuscript has made significant progress in clarifying methodology and enhancing transparency. Major improvements include clearer reporting of post-stratification and weighting, explicit definitions of explanatory variables, more transparent regression methods, and a more cautious interpretation of associations. The ethics and data availability statements are also improved, with controlled access pathways now described. However, several important gaps remain. Recruitment and response rates are not reported, which limits the assessment of selection bias. Psychometric validation of composite scores and handling of missing data are not addressed. Vaccination status ascertainment remains unclear, and model selection criteria are not specified. Adjusted effect sizes are not highlighted in the main text, and ethics committee approvals are not explicitly mentioned. Finally, contextual factors (such as seasonality and information sources) and equity implications (urban versus rural differences) require deeper discussion.

While the manuscript is substantially stronger, further revisions are necessary to fully meet the standards of transparency, validity, reproducibility, and clarity. Addressing these remaining issues will make the study suitable for publication and maximize its policy relevance.
