# Supplementary material for: Assessing dengue knowledge, attitudes and preventive practices in France and Italy: A cross-sectional study using participatory surveillance cohorts
Source: PLOS Glob Public Health. 2026 Sep 23;6(9):e0007162. doi: 10.1371/journal.pgph.0007162 (PMC13600492; doi:10.1371/journal.pgph.0007162)
Supplement: Peer review history

# Author comment: Author response to Decision Letter 0

**DOI:** 10.1371/journal.pgph.0007162.r001

**Transfer Alert**

This paper was transferred from another journal. As a result, its full editorial history (including decision letters, peer reviews and author responses) may not be present.

18 Feb 2026

# Peer review: Decision Letter 0

**DOI:** 10.1371/journal.pgph.0007162.r002

**Authors:** Muhammad Asaduzzaman

29 Apr 2026

PGPH-D-26-00275

Assessing dengue knowledge, attitudes and preventive practices: a multicountry digital cohorts study

PLOS Global Public Health

Dear Dr. Alessandro De Gaetano,

Thank you for submitting your manuscript to PLOS Global Public Health. After careful consideration, we feel that it has merit but does not fully meet PLOS Global Public Health’s publication criteria as it currently stands. Therefore, we invite you to submit a revised version of the manuscript that addresses the points raised during the review process.

Please submit your revised manuscript by May 30, 2026. If you will need more time than this to complete your revisions, please reply to this message or contact the journal office at globalpubhealth@plos.org. When you're ready to submit your revision, log on to https://www.editorialmanager.com/pgph/ and select the 'Submissions Needing Revision' folder to locate your manuscript file.

Please include the following items when submitting your revised manuscript:

- A letter that responds to each point raised by the editor and reviewer(s). You should upload this letter as a separate file labeled 'Response to Reviewers'.
- A marked-up copy of your manuscript that highlights changes made to the original version. You should upload this as a separate file labeled 'Revised Manuscript with Track Changes'.
- An unmarked version of your revised paper without tracked changes. You should upload this as a separate file labeled 'Manuscript'.

Guidelines for resubmitting your figure files are available below the reviewer comments at the end of this letter.

As the corresponding author, your ORCID iD is verified in the submission system and will appear in the published article. PLOS supports the use of ORCID, and we encourage all coauthors to register for an ORCID iD and use it as well. Please encourage your coauthors to verify their ORCID iD within the submission system before final acceptance, as unverified ORCID iDs will not appear in the published article. *Only*  the individual author can complete the verification step; PLOS staff *cannot*  verify ORCID iDs on behalf of authors.

We look forward to receiving your revised manuscript.

Kind regards,

Muhammad Asaduzzaman, MD MPH MPhil

Academic Editor

PLOS Global Public Health

Journal Requirements:

1. We ask that a manuscript source file is provided at Revision. Please upload your manuscript file as a .doc, .docx, .rtf or .tex.

2. We have noticed that you have uploaded Supporting Information files, but you have not included a list of legends. Please add a full list of legends for your Supporting Information files after the references list.

3. We note that you have indicated that there are restrictions to data sharing for this study. For studies involving human research participant data or other sensitive data, we encourage authors to share de-identified or anonymized data. However, when data cannot be publicly shared for ethical reasons, we allow authors to make their data sets available upon request. For information on unacceptable data access restrictions, please see http://journals.plos.org/plosone/s/data-availability#loc-unacceptable-data-access-restrictions.

Before we proceed with your manuscript, please address the following prompts:

a) If there are ethical or legal restrictions on sharing a de-identified data set, please explain them in detail (e.g., data contain potentially identifying or sensitive patient information, data are owned by a third-party organization, etc.) and who has imposed them (e.g., a Research Ethics Committee or Institutional Review Board, etc.). Please also provide contact information for a data access committee, ethics committee, or other institutional body to which data requests may be sent.

b) If there are no restrictions, please upload the minimal anonymized data set necessary to replicate your study findings to a stable, public repository and provide us with the relevant URLs, DOIs, or accession numbers. Please see http://www.bmj.com/content/340/bmj.c181.long for guidelines on how to de-identify and prepare clinical data for publication. For a list of recommended repositories, please see https://journals.plos.org/plosone/s/recommended-repositories. You also have the option of uploading the data as Supporting Information files, but we would recommend depositing data directly to a data repository if possible.

Please update your Data Availability statement in the submission form accordingly.

If the reviewer comments include a recommendation to cite specific previously published works, please review and evaluate these publications to determine whether they are relevant and should be cited. There is no requirement to cite these works unless the editor has indicated otherwise.

Additional Editor Comments (if provided):

[Note: HTML markup is below. Please do not edit.]

Reviewers' comments:

Reviewer's Responses to Questions

**Comments to the Author**

1. Does this manuscript meet PLOS Global Public Health’s publication criteria?

Reviewer #1: Yes

Reviewer #2: Partly

Reviewer #3: Yes

**********

2. Has the statistical analysis been performed appropriately and rigorously?

Reviewer #1: Yes

Reviewer #2: Yes

Reviewer #3: Yes

**********

3. Have the authors made all data underlying the findings in their manuscript fully available (please refer to the Data Availability Statement at the start of the manuscript PDF file)?

The PLOS Data policy

Reviewer #1: No

Reviewer #2: No

Reviewer #3: Yes

**********

4. Is the manuscript presented in an intelligible fashion and written in standard English?

Reviewer #1: Yes

Reviewer #2: Yes

Reviewer #3: Yes

**********

Reviewer #1: Overall Impression

The manuscript “Assessing dengue knowledge, attitudes and preventive practices: a multicountry digital cohorts study” addresses a timely and important public health question: how populations in mainland France and Italy perceive dengue risk and adopt preventive behaviours as Aedes vectors expand across Europe. The study leverages established participatory surveillance cohorts (InfluenzaNet) and applies bias-adjusted regression models to a large pooled sample (2,904 participants) to identify determinants of awareness, knowledge, adoption of preventive behaviour, and vaccine willingness. The work is relevant to preparedness and risk communication strategies in non-endemic settings and offers useful empirical evidence for public health planning.

Strengths

• Timeliness and relevance. The topic is highly relevant given the documented expansion of Aedes mosquitoes and recent autochthonous dengue cases in Europe.

• Use of existing participatory cohorts. Leveraging InfluenzaNet cohorts is an efficient approach that enables rapid data collection and links to rich participant profiles.

• Large pooled sample. The combined sample size (France + Italy) provides statistical power to detect associations across multiple outcomes.

• Appropriate analytic approach. The use of bias-adjusted logistic and ordinal regression models, post-stratification by age/sex/education, and assessment of proportional odds assumptions are appropriate for the outcomes described.

• Policy oriented outcomes. The manuscript examines actionable endpoints (knowledge, preventive practices, and vaccine willingness) that can inform communication and intervention strategies.

• Clear presentation of variables. Table 1 and the methods section clearly list explanatory and outcome variables, facilitating reproducibility.

Areas for Improvement

1. Sampling, Representativeness and Generalisability

• Clarify recruitment and response rates. The manuscript should report invitation numbers, response rates, and any differences between respondents and non respondents to assess selection bias.

• Limitations of participatory cohorts. InfluenzaNet participants are known to be older and more educated; while post stratification is used, discuss residual bias and how it might affect estimates of knowledge, worry, and behaviour.

• Pooling rationale. Provide more detail and justification for pooling France and Italy (e.g., tests of homogeneity, country interaction terms). If important country-level differences exist, consider presenting stratified results or country-by-predictor interactions.

2. Measurement and Construct Validity

• Knowledge and behaviour scales. Provide psychometric information (e.g., internal consistency, item response distributions) for the Knowledge score, Efficacy score, and Worry level. Report how missing items were handled.

• Outcome definitions. The binary Symptoms variable is defined by a set of rare symptoms (e.g., blood in feces/vomit). Explain the rationale for this definition and report its prevalence; consider sensitivity analyses using alternative symptom definitions.

• Self reported vaccination. Given the low reported vaccination prevalence (0.4%), discuss potential misclassification (recall, misunderstanding of vaccine type) and whether respondents were given vaccine brand examples.

3. Statistical Analysis and Reporting

• Model selection transparency. The manuscript mentions backward stepwise selection; report the selection criteria (p value thresholds, information criteria) and whether results are robust to alternative model specifications (e.g., full models, LASSO).

• Effect size presentation. For key predictors, present adjusted effect sizes (odds ratios or proportional odds ratios) with 95% confidence intervals and p-values in the main tables; highlight the most policy-relevant associations in the text.

• Handling of weights. Describe how post stratification weights were incorporated into regression models (e.g., survey weighted regression, robust standard errors) and whether variance estimation accounted for weighting.

• Multiple comparisons. Given many outcomes and predictors, discuss the approach to multiple testing or emphasize effect sizes and consistency rather than isolated p values.

4. Contextual and Temporal Factors

• Timing of data collection. Data were collected May-October 2024; discuss how seasonal variation and contemporaneous outbreaks (e.g., the Italy 2024 outbreak) may have influenced awareness and behaviours.

• Information environment. The manuscript notes that 16% sought information; expand on sources of information (media, health authorities, social networks) and whether source type modifies knowledge or vaccine willingness.

5. Ethics, Data Availability, and Reproducibility

• Data sharing constraints. The Data Availability Statement indicates that raw data cannot be shared due to privacy concerns. Provide a clear pathway for data access (e.g., controlled access, data use agreements) and describe what de identified aggregate data or code will be made available.

• Ethics approval and consent. Explicitly state the ethics committee approvals, informed consent procedures, and data protection measures used for the InfluenzaNet cohorts and the dengue survey.

• Supplementary materials. Ensure the full questionnaire (S1 Text) and code for analyses are provided as supplementary files or via a repository to enable reproducibility.

6. Interpretation and Causal Language

• Avoid causal claims. The cross sectional design limits causal inference. Rephrase language that implies causality (e.g., “X led to Y”) and emphasize associations and potential confounding.

• Heterogeneity and equity. Expand the discussion of rural vs. urban findings and their implications for equity. Why might rural residents show lower knowledge but higher adoption of measures?

7. Clarity and Structure

• Concise Results. Some result descriptions are dense; use summary tables and figures (e.g., forest plots) to make key associations more accessible.

• Typographical and formatting issues. Address minor formatting artifacts (line breaks, numbering) present in the draft to improve readability.

Policy Implications

• Targeted risk communication. The finding that seeking information and worry levels are strong determinants of knowledge and preventive behaviours suggests public health campaigns should prioritize accessible, locally relevant information and risk communication to stimulate appropriate concern without causing undue alarm.

• Vaccination strategy messaging. With 54.6% willingness to accept a vaccine if offered but low current uptake, policymakers should prepare clear guidance on vaccine eligibility, safety, and target groups, and address the dominant reason for refusal (low perceived risk) through tailored messaging.

• Rural outreach. The observed pattern of lower knowledge and vaccine willingness but higher preventive measure adoption in rural areas indicates that interventions should be context-sensitive: leverage existing preventive behaviours while improving factual knowledge and vaccine confidence.

• Surveillance and preparedness. Participatory platforms like InfluenzaNet can be integrated into early warning and community engagement strategies for Aedes borne disease preparedness, but authorities should account for representativeness limitations when using such data for policy decisions.

Overall Recommendations for Revision

Major revisions required before acceptance

1. Strengthen sampling and representativeness reporting.

• Provide invitation and response rates, compare respondents vs. non-respondents, and expand the discussion of residual selection bias after weighting.

• Justify pooling of countries and present sensitivity or stratified analyses if heterogeneity exists.

2. Enhance measurement validity and transparency.

• Report psychometric properties of composite scores, handling of missing items, and rationale for symptom definitions.

• Clarify how vaccination status was ascertained and consider sensitivity checks.

3. Clarify statistical methods and present fuller results.

• Describe model selection criteria, how weights were used in regression, and present full adjusted estimates with confidence intervals in the main tables.

• Address multiple testing concerns or contextualize findings accordingly.

4. Improve reproducibility and ethics reporting.

• Provide ethics approval details, consent procedures, and a clear, practical data access statement (controlled access or aggregated datasets and analysis code).

• Make the questionnaire and analysis code available as supplementary materials or via a repository.

5. Refine interpretation and discussion.

• Avoid causal language; discuss alternative explanations and potential confounding.

• Expand on implications for targeted communication, rural outreach, and integration with public health surveillance.

Minor revisions

• Clean up formatting and typographical issues.

• Add concise visual summaries (figures) for main associations.

• Expand the brief discussion on the role of information sources and seasonal/contextual influences.

Conclusive Impression

The manuscript addresses an important and timely public health issue and has strong potential to inform preparedness and communication strategies in Europe. However, major revisions are necessary to improve transparency on sampling and weighting, strengthen measurement validity, clarify statistical methods and reporting, and ensure reproducibility and ethical clarity. After these revisions and a clearer presentation of the adjusted effect estimates, the manuscript would be suitable for publication in a public health journal focused on infectious disease preparedness and health communication.

Reviewer #2: Title to be reconsidered

Objectives(S) to be reframed

Methodology to be changed aligning cros-sectional study with proper sampling methods

Statistical analysis is as per standard.

Limitation and biases to be addressed more rigorously.

Reviewer #3: Lucille Calmon et al., in their manuscript intitled “Assessing dengue knowledge, attitudes and preventive practices: a multicountry digital cohorts study”, leveraged French and Italian participatory surveillance cohorts from the European network InfluenzaNet to investigate emerging threats unrelated to respiratory viruses. They collected data on knowledge, attitudes, and preventive practices related to dengue in France and Italy, where dengue is an emerging threat. Their findings revealed high awareness, moderate levels of concern, and moderate willingness to be vaccinated, highlighting the urgent need for communication campaigns to strengthen knowledge and awareness of these lesser-known aspects.

This study is dense, highly relevant, and of significant public health importance, as it allowed the authors to analyze participants’ knowledge, attitudes, behaviors, and practices related to dengue in a non-endemic context such as Europe.

I believe this study is suitable for consideration by PLOS Global Public Health for publication.

The manuscript is well written and easy to read. However, I have a few comments that could help the authors further improve this important work:

Specific Comments

Line 21: Please add “worldwide” at the end of the sentence.

Lines 66–67: Add “the” before assessment in the sentence “In 2025, guidance from the ECDC on assessment…” and delete the second “of Aedes-borne”.

Line 68: Replace “faces high numbers of importations of dengue cases in” with “reports large numbers of imported dengue cases into”.

Line 71: Replace “dengue importations” with “imported dengue cases” and “came from” with “originated from”.

Line 72: Elaborate further (why) on the statement “the dengue vaccine Qdenga was approved in Europe in 2022, but is not recommended in mainland France.”

Line 81: Replace “benefits gained from the” with “perceived benefits of”.

Lines 93–96: Specify the country/countries where these observations were made.

Lines 107–111: The sentence is too long. Consider splitting it into two and replacing “but” with “However”. Also revise as follows:

- “in a sample randomly selected in colonised departments” to “in a randomly selected sample from colonized departments”

- “found an increasing awareness of the mode of transmission of dengue” to “revealed increasing awareness of dengue transmission”

- “knowledge of other mosquito-borne viruses characteristics” to “knowledge of the characteristics of other mosquito-borne viruses”

Line 159: Replace:

- “between” by “from”

- “The data was” by “Data were”

- “collected to capture” by “intended to capture”

- “responses filled out until” by “responses submitted until”

Statistical analysis:

- Rephrase “ordinality of the different levels of knowledge and measure adoption” as “the ordered nature of knowledge levels and preventive measure adoption”.

- Avoid repeating “analyses were conducted”.

- Change “called in python with r2py” to “accessed in Python via rpy2”.

Explanatory variables:

- Avoid repetition of participants’ intake survey.

- Change “education attainment level” to “educational attainment”.

- Change “the presence of at least one comorbidity” to “presence of at least one comorbidity”.

Line 277: Clarify what other commonly reported reasons for refusing vaccination were. It is known that in France, “historical mistrust” and “fear of side effects” are among the leading reasons of refusing vaccination…

Ethics approval and consent to participate:

- “In France, the Grippenet/Covidnet study was approved, and in Italy, the influweb.it study was conducted in accordance with the Italian Data Protection Authority. However, as these approvals were initially not intended for research on dengue or other diseases, it is expected that an approved addendum should be provided by the authors, stipulating that the authorized cohorts may also be used for additional studies.”

Results:

- Change “while the French sample was biased towards females” to “whereas the French sample was skewed toward females”.

Fear and vaccination:

- It would be useful to know the other most frequently cited reasons for refusing vaccination if offered.

Discussion (Lines 699–701):

- I do not agree with the authors’ assumption that future vaccines against dengue would be well received in France and Italy based the refusal reasons I cited above. Si please elaborate further on this.

**********

what does this mean?). If published, this will include your full peer review and any attached files.

**Do you want your identity to be public for this peer review?**  If you choose “no”, your identity will remain anonymous but your review may still be made public.

For information about this choice, including consent withdrawal, please see our Privacy Policy

Reviewer #1: **Yes:**  Awah Kum Tchouaffi

Reviewer #2: **Yes:**  Dr Murari Mohan Mandal, MD PhD

Reviewer #3: **Yes:**  Maurice Demanou

**********

[NOTE: If reviewer comments were submitted as an attachment file, they will be attached to this email and accessible via the submission site. Please log into your account, locate the manuscript record, and check for the action link "View Attachments". If this link does not appear, there are no attachment files.]

Attachment Submitted filename: Review Summary_PLOS_20April2026.docx

Attachment Submitted filename: PGPH-D-26-00275 MD Comments.pdf

# Author comment: Author response to Decision Letter 1

**DOI:** 10.1371/journal.pgph.0007162.r003

2 Jun 2026

Attachment Submitted filename: response_to_reviewers.pdf

# Peer review: Decision Letter 1

**DOI:** 10.1371/journal.pgph.0007162.r004

**Authors:** Muhammad Asaduzzaman

17 Jun 2026

PGPH-D-26-00275R1

Assessing dengue knowledge, attitudes and preventive practices in France and Italy: a cross-sectional study using participatory surveillance cohorts

PLOS Global Public Health

Dear Dr. Lucille Calmon,

Thank you for submitting your manuscript to PLOS Global Public Health. After careful consideration, we feel that it has merit but does not fully meet PLOS Global Public Health’s publication criteria as it currently stands. Therefore, we invite you to submit a revised version of the manuscript that addresses the points raised during the review process.

Please submit your revised manuscript by July 05, 2026. If you will need more time than this to complete your revisions, please reply to this message or contact the journal office at globalpubhealth@plos.org. When you're ready to submit your revision, log on to https://www.editorialmanager.com/pgph/ and select the 'Submissions Needing Revision' folder to locate your manuscript file.

Please include the following items when submitting your revised manuscript:

- A letter that responds to each point raised by the editor and reviewer(s). You should upload this letter as a separate file labeled 'Response to Reviewers'.
- A marked-up copy of your manuscript that highlights changes made to the original version. You should upload this as a separate file labeled 'Revised Manuscript with Track Changes'.
- An unmarked version of your revised paper without tracked changes. You should upload this as a separate file labeled 'Manuscript'.

Guidelines for resubmitting your figure files are available below the reviewer comments at the end of this letter.

As the corresponding author, your ORCID iD is verified in the submission system and will appear in the published article. PLOS supports the use of ORCID, and we encourage all coauthors to register for an ORCID iD and use it as well. Please encourage your coauthors to verify their ORCID iD within the submission system before final acceptance, as unverified ORCID iDs will not appear in the published article. *Only*  the individual author can complete the verification step; PLOS staff *cannot*  verify ORCID iDs on behalf of authors.

We look forward to receiving your revised manuscript.

Kind regards,

Muhammad Asaduzzaman, MD MPH MPhil

Academic Editor

PLOS Global Public Health

Journal Requirements:

If the reviewer comments include a recommendation to cite specific previously published works, please review and evaluate these publications to determine whether they are relevant and should be cited. There is no requirement to cite these works unless the editor has indicated otherwise.

Please review your reference list to ensure that it is complete and correct. If you have cited papers that have been retracted, please include the rationale for doing so in the manuscript text, or remove these references and replace them with relevant current references. Any changes to the reference list should be mentioned in the rebuttal letter that accompanies your revised manuscript. If you need to cite a retracted article, indicate the article’s retracted status in the References list and also include a citation and full reference for the retraction notice.

Additional Editor Comments (if provided):

[Note: HTML markup is below. Please do not edit.]

Reviewers' comments:

Reviewer's Responses to Questions

**Comments to the Author**

Reviewer #1: All comments have been addressed

Reviewer #3: All comments have been addressed

**********

publication criteria?

Reviewer #1: Yes

Reviewer #3: Yes

**********

3. Has the statistical analysis been performed appropriately and rigorously?

Reviewer #1: No

Reviewer #3: Yes

**********

4. Have the authors made all data underlying the findings in their manuscript fully available (please refer to the Data Availability Statement at the start of the manuscript PDF file)?

The PLOS Data policy

Reviewer #1: No

Reviewer #3: Yes

**********

5. Is the manuscript presented in an intelligible fashion and written in standard English?

Reviewer #1: Yes

Reviewer #3: Yes

**********

Reviewer #1: The revised manuscript has made significant progress in clarifying methodology and enhancing transparency. Major improvements include clearer reporting of post-stratification and weighting, explicit definitions of explanatory variables, more transparent regression methods, and a more cautious interpretation of associations. The ethics and data availability statements are also improved, with controlled access pathways now described. However, several important gaps remain. Recruitment and response rates are not reported, which limits the assessment of selection bias. Psychometric validation of composite scores and handling of missing data are not addressed. Vaccination status ascertainment remains unclear, and model selection criteria are not specified. Adjusted effect sizes are not highlighted in the main text, and ethics committee approvals are not explicitly mentioned. Finally, contextual factors (such as seasonality and information sources) and equity implications (urban versus rural differences) require deeper discussion.

While the manuscript is substantially stronger, further revisions are necessary to fully meet the standards of transparency, validity, reproducibility, and clarity. Addressing these remaining issues will make the study suitable for publication and maximize its policy relevance.

Reviewer #3: Assessing dengue knowledge, attitudes and preventive practices in France and Italy: a cross-sectional study using participatory surveillance cohorts R1, by Lucille Calmon et al.

I would like to thank the authors for their detailed and thoughtful responses to my comments and to other reviewers’ comments. I appreciate the clarifications provided, particularly regarding the recommendations for the Qdenga vaccine in mainland France, the specification of refusal reasons for vaccination, and the ethical approval framework in France and Italy.

The revisions made to the abstract, introduction, methodology, and results sections have significantly improved the clarity and accuracy of the manuscript. I also acknowledge the careful rephrasing of several sentences, which has enhanced readability and scientific precision.

Overall, I am satisfied with the authors’ responses and the changes implemented. I agree that the manuscript has been strengthened and is now more suitable for publication in PLOS Global Public Health.

**********

what does this mean?). If published, this will include your full peer review and any attached files.

**Do you want your identity to be public for this peer review?**  If you choose “no”, your identity will remain anonymous but your review may still be made public.

For information about this choice, including consent withdrawal, please see our Privacy Policy

Reviewer #1: **Yes:**  Awah Kum Tchouaffi

Reviewer #3: **Yes:**  Maurice Demanou

**********

[NOTE: If reviewer comments were submitted as an attachment file, they will be attached to this email and accessible via the submission site. Please log into your account, locate the manuscript record, and check for the action link "View Attachments". If this link does not appear, there are no attachment files.]

Attachment Submitted filename: PGPH-D-26-00275 R1 MD Comments.pdf

Attachment Submitted filename: Review Statement Manuscript Number PGPH-D-26-00275R1.docx

# Author comment: Author response to Decision Letter 2

**DOI:** 10.1371/journal.pgph.0007162.r005

4 Jul 2026

Attachment Submitted filename: Response to reviewers.pdf

# Peer review: Decision Letter 2

**DOI:** 10.1371/journal.pgph.0007162.r006

**Authors:** Muhammad Asaduzzaman

6 Aug 2026

Assessing dengue knowledge, attitudes and preventive practices in France and Italy: a cross-sectional study using participatory surveillance cohorts

PGPH-D-26-00275R2

Dear Lucille Calmon,

We are pleased to inform you that your manuscript 'Assessing dengue knowledge, attitudes and preventive practices in France and Italy: a cross-sectional study using participatory surveillance cohorts' has been provisionally accepted for publication in PLOS Global Public Health.

Before your manuscript can be formally accepted you will need to complete some formatting changes, which you will receive in a follow up email. A member of our team will be in touch with a set of requests.

Please note that your manuscript will not be scheduled for publication until you have made the required changes, so a swift response is appreciated.

IMPORTANT: The editorial review process is now complete. PLOS will only permit corrections to spelling, formatting or significant scientific errors from this point onwards. Requests for major changes, or any which affect the scientific understanding of your work, will cause delays to the publication date of your manuscript.

If your institution or institutions have a press office, please notify them about your upcoming paper to help maximize its impact. If they'll be preparing press materials, please inform our press team as soon as possible -- no later than 48 hours after receiving the formal acceptance. Your manuscript will remain under strict press embargo until 2 pm Eastern Time on the date of publication. For more information, please contact globalpubhealth@plos.org.

Thank you again for supporting Open Access publishing; we are looking forward to publishing your work in PLOS Global Public Health.

Best regards,

Muhammad Asaduzzaman, MD MPH MPhil

Academic Editor

PLOS Global Public Health

***********************************************************

Reviewer Comments (if any, and for reference):

Reviewer's Responses to Questions

**Comments to the Author**

Reviewer #1: All comments have been addressed

**********

publication criteria?

Reviewer #1: Yes

**********

3. Has the statistical analysis been performed appropriately and rigorously?

Reviewer #1: Yes

**********

4. Have the authors made all data underlying the findings in their manuscript fully available (please refer to the Data Availability Statement at the start of the manuscript PDF file)?

The PLOS Data policy

Reviewer #1: Yes

**********

5. Is the manuscript presented in an intelligible fashion and written in standard English?

Reviewer #1: Yes

**********

Reviewer #1: In the previous rounds of review, I raised several methodological, reporting, and clarity-related concerns. The authors have systematically responded to each point, revising the manuscript accordingly. Key improvements include clearer methodological descriptions, transparent data handling, and strengthened contextual discussion. No outstanding issues remain, and the revisions demonstrate responsiveness and commitment to quality. This satisfies the requirement for adequate revision.

The manuscript aligns with the journal’s scope and standards. It contributes to global public health knowledge by presenting evidence that is relevant, timely, and policy-informing. The study design is technically sound, and the conclusions are supported by the data presented. The work demonstrates originality, methodological rigor, and practical relevance, meeting the publication criteria.

The statistical methods employed are appropriate for the research questions and data type. The authors have clarified their regression models, explained variable selection, and reported effect sizes with transparency. Assumptions underlying the analyses appear to have been respected, and the reporting is sufficiently detailed to allow reproducibility. The robustness of the statistical approach supports the validity of the findings.

The authors have made all underlying data accessible in accordance with PLOS policies. Data availability statements are clear, and pathways for controlled access are described where necessary. This ensures transparency, reproducibility, and compliance with open science standards. The availability of data strengthens the credibility of the manuscript and allows independent verification.

The manuscript is presented in a clear, logical, and intelligible manner. The narrative flows coherently, with well-structured sections that guide the reader through the study. The English language usage meets academic standards, with precise terminology and minimal grammatical errors. The clarity of writing enhances accessibility for a broad readership, including policymakers and practitioners.

After multiple rounds of review and careful consideration of the revisions, I am satisfied that the manuscript now meets the standards of technical soundness, transparency, and clarity required for publication in PLOS Global Public Health. All reviewer comments have been addressed, the data support the conclusions, and the work is presented in a professional and intelligible manner.

**********

what does this mean?). If published, this will include your full peer review and any attached files.

**Do you want your identity to be public for this peer review?**  If you choose “no”, your identity will remain anonymous but your review may still be made public.

For information about this choice, including consent withdrawal, please see our Privacy Policy

Reviewer #1: **Yes:**  Awah Kum Tchouaffi

**********
